# Phylogenetic relationship of dengue virus type 3 isolated in Brazil and Paraguay and global evolutionary divergence dynamics

**DOI:** 10.1186/1743-422X-9-124

**Published:** 2012-06-20

**Authors:** Helda Liz Alfonso, Alberto Anastacio Amarilla, Paula Fernanda Gonçalves, Matheus Takatuba Barros, Flavia Tremeschin de Almeida, Telma R Silva, Eliana V da Silva, Marcio T Nunes, Pedro F C Vasconcelos, Deusilene S Vieira, Weber Cheli Batista, Maria Liz Bobadilla, Cynthia Vazquez, Mirian Moran, Luiz Tadeu Moraes Figueiredo, Victor Hugo Aquino

**Affiliations:** 1 Departamento de Análises Clínicas, Toxicológicas e Bromatológicas, Faculdade de Ciências Farmacêuticas de Ribeirão Preto, Universidade de São Paulo, Av. do Café s/n., 14040-903, Ribeirão Preto, São Paulo, Brazil; 2 Departamento de Arbovirologia e Febres Hemorrágicas, Instituto Evandro Chagas, Rodovia BR-316, km-7, 67030-000, Ananindeua, PA, Brazil; 3 Fundação Oswaldo Cruz – Noroeste, Instituto de Pesquisas em Patologias Tropicais de Rondônia, BR 364, km 9.5, 76800-000 , Porto Velho, RO, Brazil; 4 Departamento de Virología, Laboratorio Central de Salud Pública, Ministerio de Salud y Bienestar Social, Av. Venezuela, s/n , Asunción, Paraguay; 5 Centro de Pesquisa em Virologia, Faculdade de Medicina de Ribeirão Preto, Universidade de São Paulo, Av. Bandeirantes, 3900, Ribeirão Preto, São Paulo, 14049-900 , Brazil

## Abstract

**Background:**

Dengue is the most important mosquito-borne viral disease worldwide. Dengue virus comprises four antigenically related viruses named dengue virus type 1 to 4 (DENV1-4). DENV-3 was re-introduced into the Americas in 1994 causing outbreaks in Nicaragua and Panama. DENV-3 was introduced in Brazil in 2000 and then spread to most of the Brazilian States, reaching the neighboring country, Paraguay in 2002. In this study, we have analyzed the phylogenetic relationship of DENV-3 isolated in Brazil and Paraguay with viruses isolated worldwide. We have also analyzed the evolutionary divergence dynamics of DENV-3 viruses.

**Results:**

The entire open reading frame (ORF) of thirteen DENV-3 isolated in Brazil (n = 9) and Paraguay (n = 4) were sequenced for phylogenetic analysis. DENV-3 grouped into three main genotypes (I, II and III). Several internal clades were found within each genotype that we called lineage and sub-lineage. Viruses included in this study belong to genotype III and grouped together with viruses isolated in the Americas within the lineage III. The Brazilian viruses were further segregated into two different sub-lineage, A and B, and the Paraguayan into the sub-lineage B. All three genotypes showed internal grouping. The nucleotide divergence was in average 6.7% for genotypes, 2.7% for lineages and 1.5% for sub-lineages. Phylogenetic trees constructed with any of the protein gene sequences showed the same segregation of the DENV-3 in three genotypes.

**Conclusion:**

Our results showed that two groups of DENV-3 genotypes III circulated in Brazil during 2002–2009, suggesting different events of introduction of the virus through different regions of the country. In Paraguay, only one group DENV-3 genotype III is circulating that is very closely related to the Brazilian viruses of sub-lineage B. Different degree of grouping can be observed for DENV-3 and each group showed a characteristic evolutionary divergence. Finally, we have observed that any protein gene sequence can be used to identify the virus genotype.

## Background

Dengue is the most important mosquito-borne viral disease in tropical and subtropical regions. An estimated 50 million dengue infections occur annually and approximately 2.5 billion people live in dengue endemic countries [1]. Dengue virus (DENV) infection can be assyntomatic or lead to a wide spectrum of clinical manifestation, ranging from an undifferentiated fever, the self-limiting non-severe dengue fever (DF), to the severe dengue haemorrhagic fever (DHF), sometimes with fatal outcomes.

DENV is an enveloped virus and comprise four distinct serotypes (DENV1-4) that belong to the genus *Flavivirus*, family *Flaviviridae*. The viral genome is constituted by a single copy of a single-stranded, positive-sense RNA of approximately 11 kb in size, containing a single open reading frame (ORF), flanked by untranslated regions (5′UTR and 3′UTR) [2]. The ORF encodes a single polyprotein, which is co- and pos-translationally cleaved into 3 structural (C, prM and E) and 7 nonstructural proteins (NS1-NS2A-NS2B-NS3-NS4A-NS4B-NS5) [3].

DENV-3 was re-introduced into the Americas in 1994, specifically in Nicaragua and Panama, and then spread to other Central American countries, Mexico, the Caribbean countries, and finally South America [4-10]. In 2001/2002, a large outbreak of DENV-3 occurred in Rio de Janeiro [9,11]. DENV-3 demonstrated its greatest epidemic potential, spreading into most of the Brazilian States and, by March 2002, into the neighboring country, Paraguay [9,12]. Our previous phylogenetic study based on the E protein gene and the 3′UTR has suggested that DENV-3 was introduced into Brazil through Rio de Janeiro as well as by the Northern Region, at least in three different occasions and subsequently has spread to Paraguay [12]. Several phylogenetic studies using partial genomic sequences of DENV were carried out to analyze its molecular epidemiology [13-16]. However, it is believed that a better picture of the dynamics of viral populations could be analyzed by sequencing the entire viral genome. Recently, complete genome analyses have been performed to study DENV phylodinamics in Singapore and India [17,18]. In the present study, we have analyzed the phylogenetic relationship of 13 DENV-3 isolated in Brazil and Paraguay with viruses isolated worldwide, using their entire RNA genome sequences.

## Results

### Genome sequencing and phylogenetic analysis of viruses isolated in Brazil and Paraguay

Six fragments with overlapping regions amplified by RT-PCR were subjected to direct nucleotide sequencing to obtain the full-length genome sequence of the 13 viruses included in this study (Table 1). The assembled sequences showed that most of the viruses have a genome of 10,707 nucleotides, with the exception of D3BR/MR9/2003 isolate, which showed a deletion of 8 nucleotides between positions 10,276 and 10,284 at the 3′UTR of the viral genome. The same deletion was also observed in sequences retrieved from the GenBank for 7 viruses isolated in Brazil, 1 in Puerto Rico, and 25 in Vietnam (Additional file 1).

**Table 1:** DENV-3 from Brazil and Paraguay used in this study

**Virus**	**Passage (No.)**	**City/State/Region**	**Country/year of isolation**
D3BR/SL3/02	2	São Luis/Maranhão/Northeast	Brazil/2002
D3BR/BV4/02	2	Boa Vista/Roraima/North	Brazil/2002
D3BR/CU6/02	2	Cuiabá/Mato Grosso/Midwest	Brazil/2002
D3BR/RP1/03	2	Ribeirão Preto/São Paulo/Southeast	Brazil/2003
D3BR/PV1/03	2	Porto Velho/Rondônia/North	Brazil/2003
D3BR/MR9/03	2	Marituba/Pará/North	Brazil/2003
D3BR/BR8/04	2	Bragança/Pará/North	Brazil/2004
D3BR/ACN/2007	2	Ribeirão Preto/São Paulo/Southeast	Brazil/2007
D3BR/AL95/2009	2	Ribeirão Preto/São Paulo/Southeast	Brazil/2009
D3PY/AS12/02	2	Asunción/Central/Eastern	Paraguay/2002
D3PY/PJ4/03	2	Pedro Juan Caballero/Amambay/Eastern	Paraguay/2003
D3PY/AS10/03	2	Asunción/Central/Eastern	Paraguay/2003
D3PY/SUS/2003	2	Asunción/Central/Eastern	Paraguay/2003

**Figure 1:**
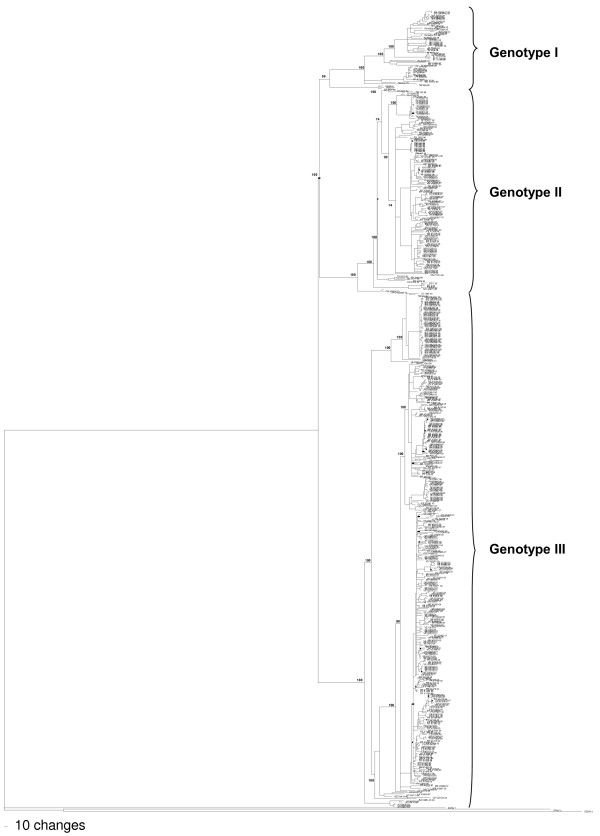
**Neighbor-joining phylogenetic trees based on the entire ORFs derived from 542 global samples of the DENV-3 inferred with PAUP program. ** The bootstrap are indicated at important nodes. The best-fit model of nucleotide substitution for phylogenetic reconstruction used was TrN + I + G model with gamma-distributed rate variation (G = 0.9908) and a proportion of invariable sites (I = 0.3678). Branch lengths are proportional to percentage of divergence.

For phylogenetic analysis, we retrieved from the GenBank the so-called complete DENV-3 sequences. However, several sequences lack 5′ and 3′ ends or had the UTRs not validated. Therefore, in the alignment we included only the open reading frame sequences (10,168 nucleotides long). Thus, the alignment included our 13 sequences and 527 sequences of DENV-3 deposited in the GenBank. Based on this alignment, a phylogenetic tree was constructed (Figure 1), showing that DENV-3 comprises three genetic groups or genotypes (I, II and III). The 13 isolates described in this study were grouped within genotype III, together with viruses isolated in the Americas.

### Phylogenetic relationship of genotype III viruses

**Figure 2:**
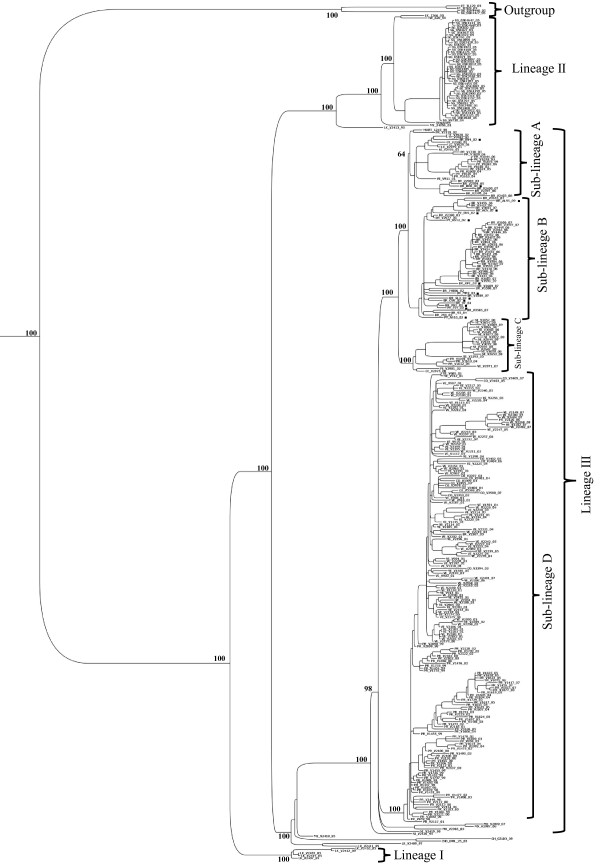
**Bayesian phylogenetic trees based on the entire ORFs derived from 347 global samples of the genotype III DENV-3 inferred with MrBayes program. ** The posterior probabilities are expressed in percent and indicated at important nodes. The best fit-model of nucleotide substitution for Bayesian phylogenetic reconstruction used was under a General Time Reversible model with gamma-distributed rate variation (G = 1.2923) and a proportion of invariable sites (I = 0.4777) (GTR + G + I).

**Figure 3:**
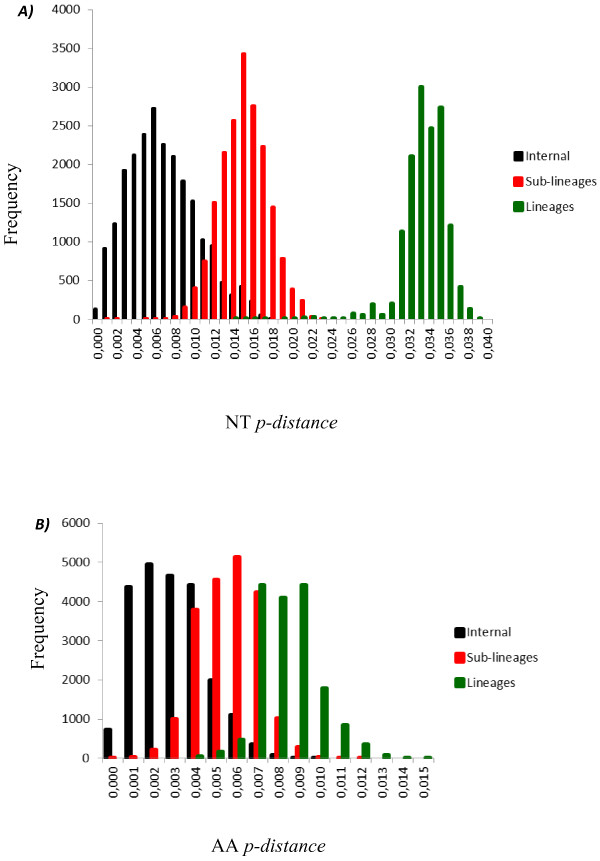
**The frequency distribution profile of pairwise distances among sequences of genotype III, based on the entire ORFs derived from 347 global samples of the genotype III DENV-3. *A ****). * The frequency distribution profile distance of nucleotide (NT). ***B****) *The frequency distribution profile distance of amino acids (AA). The NT and AA p-distance are represented on the X axis and the frequency are represented on the Y axis.

To perform a more accurate analysis of the phylogenetic relationships of viruses isolated in Brazil and Paraguay, ORF sequences of genotype III viruses (n = 347) were used to construct other phylogenetic trees using distance and Bayesian methods; these phylogenetic trees showed similar topology (Figure 2). In addition, the evolutionary divergence among sequences and the presence of amino acid motif were analyzed. Based on the topology of the tree, the frequency distribution profile of the divergence among sequences (Figure 3) and the presence of characteristic amino acid motifs (Additional file 2), genotype III viruses were clustered into three lineages (I, II and III) (Figure 2). Viruses of lineage III were further clustered into four monophyletic groups (sub-lineages A, B, C, and D) (Figure 2). The mean divergence among lineages ranged from 2.9 to 3.4% for nucleotide sequences (Table 2), and from 0.6 to 1.0% for amino acid sequences (Table 2). While the mean divergence among sub-lineages ranged from 1.0 to 1.6% for nucleotide sequences, and from 0.5 to 0.6% for amino acid sequences (Table 2). All the mean values coincided with the highest peaks in the frequency distribution profile (Figure 3). The viruses described in this study grouped together with viruses isolated in the Americas within the lineage III, distributed into two different groups, sub-lineages A and B. The Brazilian viruses isolated in the Northern Region, BR_BV4_02 from Boa Vista, Roraima, and BR_BR8_04 from Belen, Para, grouped with other Brazilian viruses isolated in the same region, with viruses isolated in the Caribbean islands (Martinique, Trinidad and Tobago, St. Lucia, Anguilla and Puerto Rico) and in the north of South America (French Guyana and Venezuela) (Figure 2, sub-lineage A). The other Brazilian isolates (BR_PV1_03, BR_CU6_02, BR_MR9_03, BR_SL3_02, BR_ACN_07, BR_AL95_09, and BR_RP1_2003) and the Paraguayan isolates (PY_AS10_03, PY_AS12_02, PY_SUS_03, PY_PJ4_03) grouped with viruses isolated in other regions of Brazil within the sub-lineage B (Figure 2, sub-lineage B). Sub-lineage C includes viruses isolated in Nicaragua, Puerto Rico, Venezuela, Peru and Ecuador. Most of the viruses isolated in Venezuela and Puerto Rico belong to the sub-lineage D, which also include viruses isolated in Colombia. Interestingly, BR_V2386_03 is the only Brazilian virus that grouped with viruses of sub-lineage D. Finally, the lineage I is composed only by old isolates of SriLanka (1983–1989) and lineage II by viruses isolated in Asia (1993–2005). In addition, viruses from Asian (two viruses from SriLanka isolated in 1989 and 1997; one virus from China isolated in 2009) and East Africa (one virus from Mozambique isolated in 1985 and one virus from India isolated in 2003) are located basally in the branch that containing the American isolates (Lineage III) and do not form a monophyletic group.

**Table 2:** Mean of the evolutionary divergence (NT and AA) from all sequence pairs between genotypes III groups

**Genotype III groups**	**NT*p-d*istance**	**%**	**AA*p-*distance**	**%**
Lineage I vs Lineage II	0.029	2.9	0.006	0.6
Lineage I vs Lineage III	0.034	3.4	0.010	1.0
Lineage II vs Lineage III	0.030	3.0	0.008	0.8
Lineage III [Sub-lineage A vs Sub-lineage B]	0.010	1.0	0.005	0.5
Lineage III [Sub-lineage A vs Sub-lineage C]	0.013	1.3	0.005	0.5
Lineage III [Sub-lineage A vs Sub-lineage D]	0.014	1.4	0.005	0.5
Lineage III [Sub-lineage B vs Sub-lineage C]	0.014	1.4	0.005	0.5
Lineage III [Sub-lineage B vs Sub-lineage D]	0.015	1.5	0.006	0.6
Lineage III [Sub-lineage C vs Sub-lineage D]	0.016	1.6	0.005	0.5

### Phylogenetic relationship among viruses from genotypes I and II

**Figure 4:**
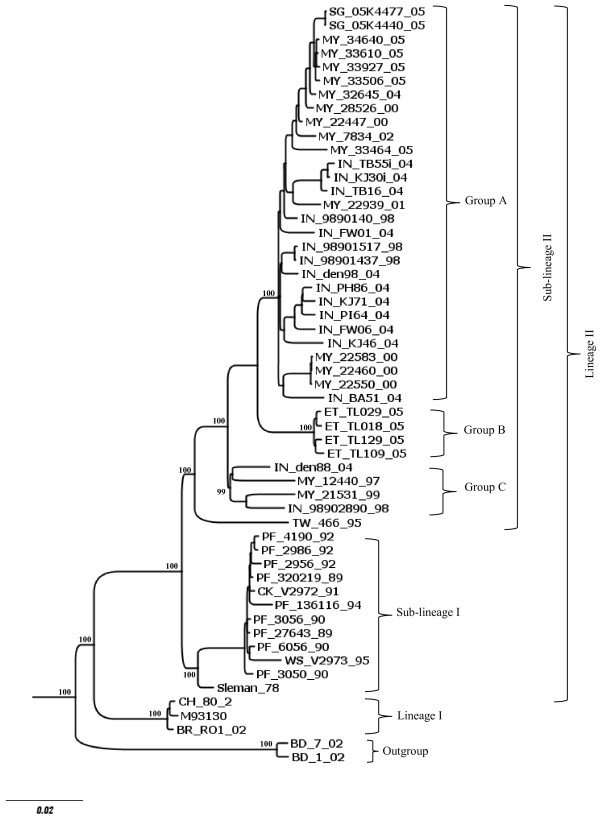
**Bayesian phylogenetic trees based on the entire ORFs derived from 53 global samples of the genotype I DENV-3 inferred with MrBayes program. ** The posterior probabilities are expressed in percent and indicated at important nodes. The best fit-model of nucleotide substitution for Bayesian phylogenetic reconstruction used was under a General Time Reversible model with gamma-distributed rate variation (G = 1.1436) and a proportion of invariable sites (I = 0.4716) (GTR + G + I).

**Figure 5:**
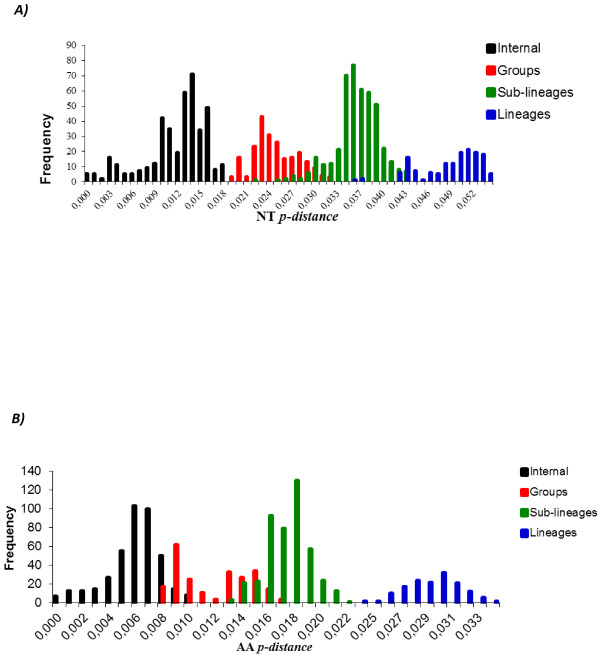
Distribution of NT (A) and AA (B) p-distances between 53 complete ORF sequences of genotype I. NT: nucleotides; AA: amino acids.

Considering that genotypes III viruses clustered into separate monophyletic groups, which we called lineages and sub-lineage, we analyzed whether a similar clustering could be observed for genotype I and II viruses. The phylogenetic relationship analysis was carried out as mentioned above for genotype III viruses. Genotype I viruses were clustered into two lineages (I and II); viruses of lineage II were segregated into two sub-lineages (I and II) and sub-lineage II includes three internal monophyletic groups (A, B and C) (Figure 4, Additional file 3). The mean nucleotide divergence between lineages I and II was 4.9% (Table 3), while the mean amino acid divergence was 1.8% (Table 3). The mean divergence among sub-lineages I and II was 3.6% for nucleotide sequences and 1.2% for amino acid sequences (Table 3). Finally, the divergence among the internal groups (A, B and C) of sub-lineage II ranged from 2.3 to 2.8% for nucleotides sequences and from 0.6 to 0.9% for amino acid sequences (Table 3). Once again, all the mean values coincided with the highest peaks in the frequency distribution profile (Figure 5).

**Table 3:** Mean of the evolutionary divergence (NT and AA) from all sequence pairs between genotypes I groups

**Genotype I groups**	**NT*p-*distance**	**%**	**AA p-distance**	**%**
Lineage I vs Lineage II	0,049	4,9	0,018	1,8
Lineage II [Sub-lineage I vs Sub-lineage II]	0,036	3,6	0,012	1,2
Sub-Lineage II [Group A vs Group B]	0,023	2,3	0,006	0,6
Sub-Lineage II [Group A vs Group C]	0,027	2,7	0,009	0,9
Sub-Lineage II [Group B vs Group C]	0,028	2,8	0,007	0,7

**Figure 6:**
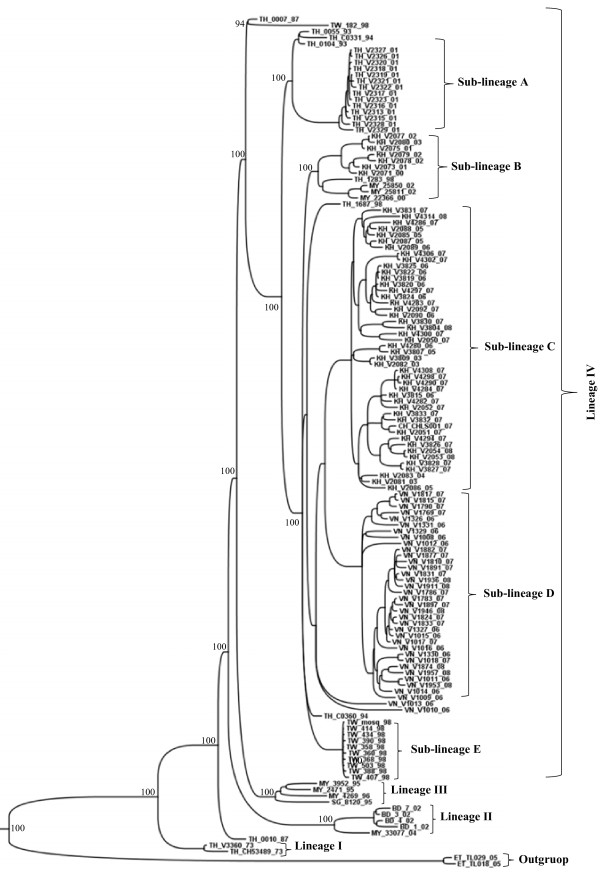
**Bayesian phylogenetic trees based on the entire ORFs derived from 137 global samples of the genotype II DENV-3 inferred with MrBayes program. ** The posterior probabilities are expressed in percent and indicated at important nodes. The best fit-model of nucleotide substitution for Bayesian phylogenetic reconstruction used was under a General Time Reversible model with gamma-distributed rate variation (G = 1.4745) and a proportion of invariable sites (I = 0.4956) (GTR + G + I). Branch lengths are proportional to percentage of divergence.

**Figure 7:**
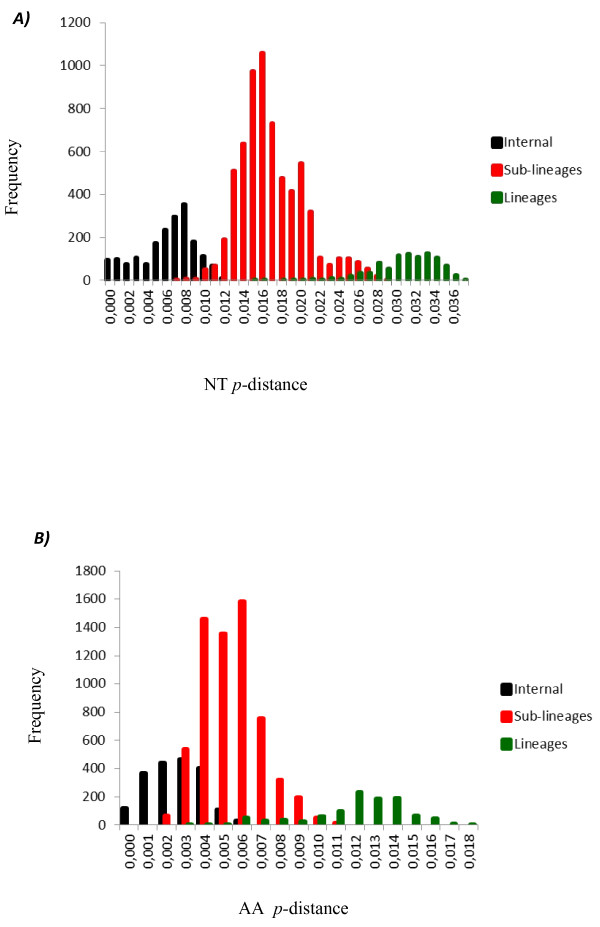
Distribution of NT (A) and AA (B) p-distances between 137 complete ORF sequences of genotype II. NT: nucleotides; AA: amino acids.

Genotype II viruses segregated into four lineages (Figures 6 and 7, Additional file 4). The lineage IV includes four sub-lineages (A, B, C and D). The mean nucleotide divergence among lineages ranged from 2.4 to 3.2% (Table 4), while the mean amino acid divergence ranged from 0.8 to 1.5% (Table 4). The mean divergence among sub-lineages ranged from 1.2 to 1.9% for nucleotide sequences and from 0.4 to 0.7% for amino acid sequences (Table 4).

**Table 4:** Mean of the evolutionary divergence (NT and AA) from all sequence pairs between genotypes II groups

**Genotype II groups**	**NT*p-*distance**	**%**	**AA*p-d*istance**	**%**
Lineage I vs Lineage II	0.031	3.1	0.015	1.5
Lineage I vs Lineage III	0.024	2.4	0.011	1.1
Lineage I vs Lineage IV	0.029	2.9	0.013	1.3
Lineage II vs Lineage III	0.027	2.7	0.012	1.2
Lineage II vs Lineage IV	0.032	3.2	0.012	1.2
Lineage III vs Lineage IV	0.024	2.4	0.008	0.8
Sub-lineage A vs Sub-lineage B	0.016	1.6	0.006	0.6
Sub-lineage A vs Sub-lineage C	0.019	1.9	0.007	0.7
Sub-lineage A vs Sub-lineage D	0.019	1.9	0.006	0.6
Sub-lineage A vs Sub-lineage E	0.015	1.5	0.006	0.6
Sub-lineage B vs Sub-lineage C	0.016	1.6	0.006	0.6
Sub-lineage B vs Sub-lineage D	0.017	1.7	0.005	0.5
Sub-lineage B vs Sub-lineage E	0.012	1.2	0.005	0.5
Sub-lineage C vs Sub-lineage D	0.015	1.5	0.004	0.4
Sub-lineage C vs Sub-lineage E	0.014	1.4	0.005	0.5
Sub-lineage D vs Sub-lineage E	0.015	1.5	0.004	0.4

### Evolutionary divergence among genotypes

**Figure 8:**
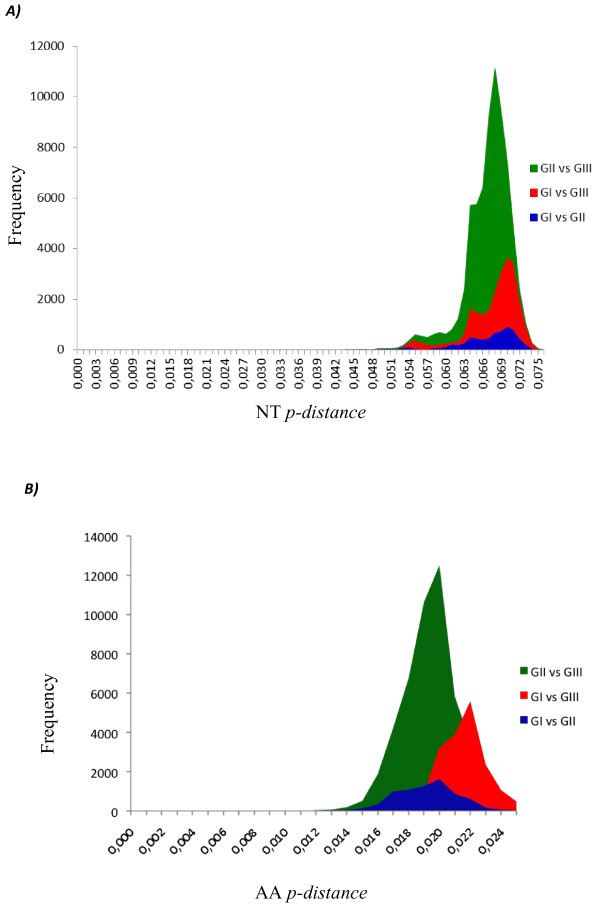
Distribution of NT (A) and AA (B) p-distances between different genotypes based on the entire ORF of 537 DENV-3. NT: nucleotides; AA: amino acids.

We have shown above the evolutionary divergence among the different monophyletic groups within each genotype. In this section, we analyzed the evolutionary divergence among genotypes using the entire ORF sequence and, individually, each protein gene sequence (Table 5 and Figure 8). The mean divergence among genotypes ranged from 6.6 to 6.8% for nucleotide and from 3.1 to 3.4% for amino acid when the entire ORF was analyzed (Figure 8). Analyzing each viral protein, the mean divergence among genotypes varied from 4.1 to 8.8% for nucleotide and from 0.5 to 5.3% for amino acid (Table 5). The lowest divergence was observed for C protein (4.1 to 4.9%) and the highest for NS2a (8.4 to 8.8%), NS4a (7.7 to 8.5) and E (7.0 to 7.6) proteins when the nucleotide sequences were analyzed (Table 4). While NS2b (0.5 to 1.0%), NS4b (0.8 to 1.1%) and NS3 (1.2 to 1.5%) proteins showed the lowest divergence and NS2a (3.0 to 3.4%) NS4a (2.8 to 3.6%) and E (2.3 to 3.0%) protein the highest divergences when the amino acid sequences were analyzed (Table 5). The means *p-*distance of the each of the genomic regions are coinciding with the highest peaks of the frequency distribution profile (Additional files 5 to 14).

**Table 5:** Mean of the evolutionary divergence from all sequence pairs between genotypes for each genomic region

**A)**											
**Genotypes**	**NT*p-*distance (%)**										
	**ORF**	**C**	**prM**	**E**	**NS1**	**NS2A**	**NS2B**	**NS3**	**NS4A**	**NS4B**	**NS5**
I vs II	6.6	4.1	6.4	7.0	6.7	8.8	6.2	6.9	8.5	5.7	6.0
I vs III	6.8	4.9	5.3	7.3	6.7	8.4	6.0	6.8	8.0	6.5	6.7
II vs III	6.7	4.5	5.6	7.6	6.4	8.5	5.9	6.7	7.7	6.6	6.3
**B)**											
**Genotypes**	**AA*p-*distance (%)**										
	**ORF**	**C**	**prM**	**E**	**NS1**	**NS2A**	**NS2B**	**NS3**	**NS4A**	**NS4B**	**NS5**
I vs II	1.9	2.8	1.8	2.5	2.4	3.4	0.5	1.5	2.8	1.0	1.6
I vs III	2.2	5.3	1.8	3.0	1.6	3.3	1.0	1.2	3.6	1.1	2.3
II vs III	1.9	2.9	1.8	2.3	1.9	3.0	0.7	1.3	3.6	0.8	2.1

### Genotypes identification

To identify which genomic region is responsible for the segregation of DENV-3 into different genotypes, several phylogenetic trees were constructed using, individually, the sequences coding for each viral protein (Additional files 15 to 24). All trees showed the same segregation of the viruses in three genotypes viruses segregated to the same genetic groups as observed when the entire ORF was used (Figure 1), except for the prM sequences, which the genotype I not formed a monophyletic group. The constructed tree based on the NS4A protein coding sequence, however, showed that CH_80_2, DENV-3 isolated from a patient in China in 1980, and, therefore, others viruses with identical sequences (M93130 and BR/RO1/02, not included in this tree), grouped with viruses from genotype II and not with those from genotype I, as observed in all other phylogenetic trees.

## Discussion

DENV-3 was introduced into the Americas via Panama and Nicaragua in 1994 [4], spreading rapidly to neighboring countries and reaching Brazil through Rio de Janeiro in December 2000 [9]. Interestingly, DENV-1 and DENV-2 were also introduced into Brazil through Rio de Janeiro [19]. Thus, it seems that the main route of entrance for DENV in Brazil was always Rio de Janeiro. Analyzing the E gene and the 3′UTR sequences, we have previously found that isolates BR/BV4/02 isolated in Boa Vista, Roraima, and BR8/04 isolated Belem, Para, in the Northern Region of the country, were phylogenetically more closely related to viruses isolated in the Caribbean islands rather than to those isolated in Rio de Janeiro, suggesting that DENV-3 was also introduced into Brazil by the northern region [12]. In the present study, now analyzing the entire ORF, the isolates BR/BV4/02 and BR/BR8/04, as well as new viruses isolated between 2006 and 2007 by other groups, were phylogenetically more closely related to viruses isolated in the Caribbean islands than to viruses circulating in Brazil, supporting the hypothesis that DENV-3 was introduced by the Norther Region of the country in addition to the well documented introduction by Rio de Janeiro. According to our phylogenetic analysis, at least two groups of DENV-3 genotype III are circulating in Brazil (Figure 2, sub-lineages A and B). A single virus (BR_V2387_03, FJ850079) isolated in the Northern Region of Brazil was more closely related to viruses isolated in Venezuela, suggesting that was an imported case. In addition to the DENV-3 genotype III, recent studies have shown that genotype I viruses are also circulating in Brazil [20-22]. The Paraguayan isolates were closely related to viruses isolated in Brazil within sub-lineage B of lineage III, suggesting that these viruses were introduced into Paraguay from Brazil as previously described [12].

A previous phylogenetic analysis of DENV-3 genotype III isolated in Sri Lanka, based on a 966 nt fragment spanning part of capsid, preM/M and part of E genes, identified the emergence, after 1989, of a new sub-type, which was correlated with severe disease epidemics that spread to Africa and then to the Americas [15]. Our phylogenetic analysis showed a similar distribution of DENV-3 genotype III isolates (Figure 2). Thus, our lineage I corresponds to DENV-3 genotype III circulating in SriLanka before 1989, which was called as sub-lineage A by Messer and colleagues (2003). On the other hand, LK_V2411_89 and LK_V2409_97 viruses, located basally in the branch of lineage III, could correspond to the new sub-type described by Messer and colleagues (2003) that they called Group B. The topology of our phylogenetic tree suggests that this last sub-type migrated to East Africa and Indian subcontinent and later to the Americas in agreement with observations made by Messer and colleagues (2003). Interestingly, phylogentic analysis of genotype III showed also that NI/V2420/1994 virus isolated in Nicaragua is located at the base of the branch containing the American isolates (Lineage III), suggesting that the introduction of genotype III in the Americas occurred through Nicaragua in 1994, in agreement with the epidemiological data [4,23].

Previous studies analyzing the C, PrM, E and NS3 genes have shown that DENV-3 segregated into four genotypes [13,14,24,25]. In this study, we have found that DENV-3 segregated in the same genotypes I, II and III as shown in the previous studies mentioned above, analyzing either the entire ORF or each protein gene sequence. Thus, DENV-3 genotype can be determined by sequencing any part of the ORF. The genotype IV was not observed in our analysis because no complete genome sequence of any of these viruses is available in the GenBank. Klungthong and colleagues (2008) have also suggested that any genomic region can be sequenced to determine the genotype [26]; however, these authors used the entire genomic sequences of only 12 isolates, while our results were supported by the analysis of more than 500 isolates.

Similar to other RNA viruses, DENV exhibit a high degree of genetic variation due to the non-proofreading activity of its RNA polymerase, the high rates of mutation, the immense population size, and the immunological pressure, leading to the emergence of new subtypes of DENV [27]. Recently, we have described the existence of various taxa or viral sub-types within DENV-3 genotypes by analyzing of the E protein gene [28]. In this study, we have found similar, and even new, internal groups within each genotype. The segregation of the viruses into genotypes, lineage, sub-lineage and groups as suggested in this study were supported by high posterior probability, by nucleotide divergence, and by the presence of characteristic amino acid motifs.

The first studies that identify different genotypes within each DENV serotypes were based on the topology of the trees, supported by bootstrap values [13,24,29-32]. The nucleotide divergence among the genotypes of DENV-1 and DENV-2 was in average 6% when the genomic sequence corresponding to the junction E/NS1 (240 bp) was analyzed [13], and 7% in mean when the E protein gene (1,485 bp) was analyzed for DENV-1 and DENV-2 [31,32]. In this study, we carried out a more detailed analysis of the nucleotide divergence among the different genetic groups of DENV-3. We have observed that nucleotide divergence varied in average 6.7% for genotypes, 2.7% for lineages and 1.5% for sub-lineages when the complete ORF of genotypes II and III was analyzed. For genotype I, a higher nucleotide divergence rate was observed among lineages and sub-lineages.

In addition, our analysis showed that the nucleotide divergence among genotypes varied depending on the genomic region, ranging from 4.1% for C protein gene to 8.8% for NS2a protein gene. These comparisons showed also that C protein gene sequence is the most conserved, and NS2a, NS4a, E protein genes the more variables. However, when the amino acid divergence was analyzed, a different picture was observed; NS2b, NS4b and NS3 were the more conserved proteins, while NS2a, NS4a, E proteins were the more variables. Knowledge of the rates of divergence among the different taxonomic levels, are an important tool for the detection of new viral groups, as well as, information about the variability of each of the genes among different viral groups, could be used to select targets for: the design of probes for diagnosis, antiviral therapies and the construction of candidate vaccines.

Recently, Wittke and colleagues have suggested the existence of an additional genotype (genotype V) within DENV-3 [33]. Our previous phylogenetic analysis based on the E protein, however, has suggested that the genotype V corresponds to a lineage within the genotype I [28]. In this study, the nucleotide divergence among lineage I (called genotype V by Wittke and colleagues) and lineage II of genotype I was 4.9% in average, lower than the 6% observed among genotypes. Therefore, we suggest the maintenance of the classification of DENV-3 into four genotypes as previously proposed [14,24].

In this work, we performed phylogenetic analysis and evolutionary divergence dynamics of DENV-3 and provided data related to the processes that control the viral evolution. These data will be useful to better characterize the DENV-3 epidemics in future and might even be used for selection of vaccine candidates.

## Methods

### Virus

Thirteen DENV-3 isolated from different regions of Brazil (n = 9) and Paraguay (n = 4) were included in this study. All viruses were isolated and passed twice in C6/36 [34]. Ten of them (D3BR/RP1/03, D3BR/PV1/03, D3BR/SL3/02, D3BR/BV4/02, D3BR/CU6/02, D3BR/BR8/04, D3BR/MR9/03, D3PY/PJ4/03, D3PY/AS10/03, D3PY/AS12/02) belong to different clusters in the phylogenetic trees constructed based on the of E gene and 3′UTR region sequences as previously described [12]. Two more recently isolates (D3BR/ACN/07 and D3BR/AL95/09) describe in other study were also included [12,35]. The Paraguayan isolate D3PY/SUS/2003 was also included because it was never characterized by sequencing.

### RNA extraction

Viral RNA was extracted from 140 μl of supernatant of infected C6/36 cells using the QIAamp Viral RNA kit (Qiagen, Germany), following the manufacturer’s recommendations. The RNA was eluted with 80 μl of DNase/RNase free water.

### RT-PCR

#### cDNA synthesis

The reaction of cDNA synthesis contained 24 μl of RNA, 200 ng of random primers (Invitrogen, USA), 0.25 mM dNTPs mix (Invitrogen, USA), 80 U of inhibitor RNAse (RNAseOUT, Invitrogen, USA), 400 U of M-MLV Reverse Transcriptase (USB, USA) and 8 μl 5X buffer (250 mM Tris–HCl [pH 8.3], 375 mM KCl, 15 mM MgCl_2_) in a final volume of 40 μl. The mixture was incubated at 25°C for 10 min, followed by incubation at 37°C for 4 h, and a final incubation of 5 min at 85°C. Subsequently, the cDNA was treated with 1 U RNase H (GIBCO, USA) at 37°C for 30 min, and stored at −20°C until use.

#### Polymerase Chain Reaction (PCR)

To amplify the entire viral genomes, the primers used in PCR reactions were designed based on the D3_BR74886_02 isolated (AY679147.1) (Additional file 25A). Six overlapping fragments representing the full viral genome were amplified. The master mix contained: 1 μl of cDNA, 0.2 mM dNTP, 0.3 mM of each primer, 1.5 U of Platinum® Taq DNA Polymerase High Fidelity (Invitrogen, USA), 5 μl of 10X buffer (600 mM Tris-SO_4_ [pH 8.9], 180 mM Ammonium Sulfate), and 2 mM of MgSO_4_ in a final volume of 50 μl. The amplification was performed using the MyCycler™ Thermal Cycler (BIO-RAD, USA). The reaction mixture was heated at 94°C for 2 min followed by 45 amplification cycles: 94°C for 10 s, 56°C for 1 min, 68°C for 5 min, and a final extension at 68°C for 7 min. The PCR products were subjected to electrophoresis in a 1% agarose gel and visualized under UV light after staining with ethidium bromide. Bands of DNA were purified from agarose gels using a QIAquick Gel Extraction Kit (Qiagen®, Germany) following the manufacturer’s specifications.

#### Nucleotide sequencing

The gel-purified DNA fragments were sequenced using an Applied Biosystems BigDye ddNTP capillary sequencer ABI 3130 (Applied Biosystems, USA) following the manufacturer’s specifications. Both strain of each DNA fragments were sequenced at least three times using walking primers (Additional file 25B). Viral genome sequences generated in this study were deposited in the GenBank, under the accession numbers: BR_BV4_02 (JF808118), BR_BR8_04 (JF808119), BR_AL95_09 (JF808120), BR_ACN_07 (JF808121), PY_SUS_02 (JF808122), PY_AS12_02 (JF808123), BR_MR9_03 (JF808124), BR_SL3_03 (JF808125), BR_PV1_03 (JF808126), BR_CU6_02 (JF808127), PY_PJ4_03 (JF808128), PY_AS10_02 (JF808129) and D3BR_RP1_2003 (EF643017).

The electropherograms were analyzed using the MEGA 5.0 program and the consensus assemble were carried out using the BioEdit v.7.0.9 program [36,37]. The 30 nucleotides at 5′ and 3′ ends of each fragment were deleted to avoid the influence of primers used for amplification. However, the sequences at 5′ and 3′ ends of the viral genome represent the sequences of the primers used to amplify these regions.

### Phylogenetic and evolutionary analysis

#### Database of DENV-3 sequences

A database containing complete genome sequences of DENV-3 retrieved from GenBank was prepared for phylogenetic analysis. Representative sequences of DENV-1 (AB074760), DENV-2 (M20558) and DENV-4 (AF326573) were also included. The database contained the following information: GenBank access number, isolated name, country and year of isolation. A total of 563 sequences of DENV-3 were retrieved from GenBank until March 27, 2010 (Additional file 26), which were indicated as complete genome. However, several of these sequences did not contain the first 39 and last 135 nucleotides of the viral genome, or did not have the UTRs sequences validated. Therefore, in order to use the largest number of sequences possible, we have included only the open reading frame (ORF). The sequences were analyzed using the program DAMBE 5.2.6 in order to identify identical sequences, which were excluded from the analysis (Additional file 27) [38]. In addition, mutants and clones were also excluded, resulting in a total of 527 sequences of DENV-3 available for phylogenetic analysis (Additional file 26).

#### Phylogenetic analysis

DENV-3 sequences were aligned using the program CLUSTAL X 5.2 [39]. The distance based Neighbor-joining (NJ) and/or the Bayesian inference (BI) methods were used to construct the phylogenetic trees. For NJ method, the sequences were first analyzed using the Modeltest 3.7.MacX program to identify the best nucleotide substitution model [40]. The best nucleotide substitution model was selected under the criterion hierarchical likelihood ratio tests (hLRT). Phylogenetic trees was constructed using the NJ method as implemented in the PAUP * 4.0 b10 program and statistically supported by bootstrap method using 1,000 replicates [41]. For BI method, the aligned sequences were first analyzed using the MrModeltest 2.3 for Mac OS X 10.4.11 program to identify the best model of nucleotide substitution [42]. The best nucleotide substitution model was selected under the criterion hierarchical likelihood ratio tests (hLRT). Five runs of 4 chains each (one cold and tree heated, temperature = 0.20), generating a total of 1.5 × 10^6^ generations (10% removed as burn-in) were done to ensure statistical convergence. The phylogenetic trees were inferred with MrBayes program and statistically supported by calculating the posterior probability, which was expressed in percentage [43].

#### Analysis of evolutionary divergence between genetic groups

The nucleotide and amino acid divergences were calculated using *p-*distance model with 1,000 replicates using the MEGA 5 program [36]. The frequency distribution profiles of divergences between populations were calculated as described previously [44]. The mean (%) *p-*distances between sequences were also calculated.

#### Identification of amino acid motif for each genetic group

The polyprotein sequences of each genetic group were aligned for the identification of the amino acid motifs, which were defined as amino acid substitutions present in at least 90% of the sequences within each genetic group.

## Competing interests

The authors declare that they have no competing interests.

## Authors’ contributions

Conceived and designed the experiments: HLA, AAA and VHA. Performed the experiments: HLA, AAA, PFG, MTB. Analyzed the data: HLA, AAA and VHA. Contributed reagents/materials/analysis tools: FTDA, TRS, EVDS, MTN, PFCV, DSV, WCB, MLB, CV, MM, LTF. Wrote the paper: HLA, AAA and VHA. All authors read and approved the final manuscript.

## Supplementary Material

Additional file 1 **Comparison of the 3′UTR variable region between the position 10,264 to 1,287 of several isolates belongs to genotypes I, II and III.** Sequence of isolates from genotypes I, II and III, which showed differences in 3′UTR region. Click here for file

Additional file 2 **Motifs of amino acids for the genotype III.** The file provides details on amino acid substitutions present within each genetic group of genotype III. Click here for file

Additional file 3 **Motifs of amino acids for the genotype I. **The file provides details on amino acid substitutions present within each genetic group of genotype I. Click here for file

Additional file 4 **Motifs of amino acids for the genotype II.** The file provides details on amino acid substitutions present within each genetic group of genotype II. Click here for file

Additional file 5 Distribution of NT (A) and AA (B) p-distances between different genotypes based on the C protein gene of 537 DENV-3. NT: nucleotides; AA: amino acids. Click here for file

Additional file 6 Distribution of NT (A) and AA (B) p-distances between different genotypes based on the prM protein gene of 537 DENV-3. NT: nucleotides; AA: amino acids. Click here for file

Additional file 7 Distribution of NT (A) and AA (B) p-distances between different genotypes based on the E protein gene of 424 DENV-3. NT: nucleotides; AA: amino acids. Click here for file

Additional file 8 Distribution of NT (A) and AA (B) p-distances between different genotypes based on the NS1 protein gene of 372 DENV-3. NT: nucleotides; AA: amino acids. Click here for file

Additional file 9 Distribution of NT (A) and AA (B) p-distances between different genotypes based on the NS2A protein gene of 328 DENV-3. NT: nucleotides; AA: amino acids. Click here for file

Additional file 10 Distribution of NT (A) and AA (B) p-distances between different genotypes based on the NS2B protein gene of 233 DENV-3. NT: nucleotides; AA: amino acids. Click here for file

Additional file 11 Distribution of NT (A) and AA (B) p-distances between different genotypes based on the NS3 protein gene of 431 DENV-3. NT: nucleotides; AA: amino acids. Click here for file

Additional file 12 Distribution of NT (A) and AA (B) p-distances between different genotypes based on the NS4A protein gene of 268 DENV-3. NT: nucleotides; AA: amino acids. Click here for file

Additional file 13 Distribution of NT (A) and AA (B) p-distances between different genotypes based on the NS4B protein gene of 323 DENV-3. NT: nucleotides; AA: amino acids. Click here for file

Additional file 14 Distribution of NT (A) and AA (B) p-distances between different genotypes based on the NS5 protein gene of 537 DENV-3. NT: nucleotides; AA: amino acids. Click here for file

Additional file 15 **Neighbor-joining phylogenetic trees based on C gene derived from 537 global samples of the DENV-3 inferred with MEGA 5 program. **The bootstrap are indicated at important nodes. The best-fit model of nucleotide substitution for phylogenetic reconstruction used was TrN + G model with gamma-distributed rate variation (G = 1.2). Branch lengths are proportional to percentage of divergence. Click here for file

Additional file 16 **Neighbor-joining phylogenetic trees based on prM gene derived from 283 global samples of the DENV-3 inferred with MEGA 5 program. **The bootstrap are indicated at important nodes. The best-fit model of nucleotide substitution for phylogenetic reconstruction used was TrN + G model with gamma-distributed rate variation (G = 1.2). Branch lengths are proportional to percentage of divergence. Click here for file

Additional file 17 **Neighbor-joining phylogenetic trees based on E gene derived from 424 global samples of the DENV-3 inferred with MEGA 5 program. **The bootstrap are indicated at important nodes. The best-fit model of nucleotide substitution for phylogenetic reconstruction used was TrN + G model with gamma-distributed rate variation (G = 1.2). Branch lengths are proportional to percentage of divergence. Click here for file

Additional file 18 **Neighbor-joining phylogenetic trees based on NS1 gene derived from 372 global samples of the DENV-3 inferred with MEGA 5 program. **The bootstrap are indicated at important nodes. The best-fit model of nucleotide substitution for phylogenetic reconstruction used was TrN + G model with gamma-distributed rate variation (G = 1.2). Branch lengths are proportional to percentage of divergence. Click here for file

Additional file 19 **Neighbor-joining phylogenetic trees based on NS2A gene derived from 328 global samples of the DENV-3 inferred with MEGA 5 program. **The bootstrap are indicated at important nodes. The best-fit model of nucleotide substitution for phylogenetic reconstruction used was TrN + G model with gamma-distributed rate variation (G = 1.2). Branch lengths are proportional to percentage of divergence. Click here for file

Additional file 20 **Neighbor-joining phylogenetic trees based on NS2B gene derived from 233 global samples of the DENV-3 inferred with MEGA 5 program. **The bootstrap are indicated at important nodes. The best-fit model of nucleotide substitution for phylogenetic reconstruction used was TrN + G model with gamma-distributed rate variation (G = 1.2). Branch lengths are proportional to percentage of divergence. Click here for file

Additional file 21 **Neighbor-joining phylogenetic trees based on NS3 gene derived from 431 global samples of the DENV-3 inferred with MEGA 5 program. **The bootstrap are indicated at important nodes. The best-fit model of nucleotide substitution for phylogenetic reconstruction used was TrN + G model with gamma-distributed rate variation (G = 1.2). Branch lengths are proportional to percentage of divergence. Click here for file

Additional file 22 **Neighbor-joining phylogenetic trees based on NS4A gene derived from 268 global samples of the DENV-3 inferred with MEGA 5 program. **The bootstrap are indicated at important nodes. The best-fit model of nucleotide substitution for phylogenetic reconstruction used was TrN + G model with gamma-distributed rate variation (G = 1.2). Branch lengths are proportional to percentage of divergence. Click here for file

Additional file 23 **Neighbor-joining phylogenetic trees based on NS4B gene derived from 323 global samples of the DENV-3 inferred with MEGA 5 program. **The bootstrap are indicated at important nodes. The best-fit model of nucleotide substitution for phylogenetic reconstruction used was TrN + G model with gamma-distributed rate variation (G = 1.2). Branch lengths are proportional to percentage of divergence. Click here for file

Additional file 24 **Neighbor-joining phylogenetic trees based on NS5 gene derived from 464 global samples of the DENV-3 inferred with MEGA 5 program.** The bootstrap are indicated at important nodes. The best-fit model of nucleotide substitution for phylogenetic reconstruction used was TrN + G model with gamma-distributed rate variation (G = 1.2). Branch lengths are proportional to percentage of divergence. Click here for file

Additional file 25 **Primers used for amplification and sequencing.** A) Primers used in the PCR of overlapping regions representing the entire genome of DENV3. B) Primers used for nucleotide sequencing of the genome. The file provides details on all the sequences of primers used for amplification and sequencing including in this study. Click here for file

Additional file 26 **The database of the all viruses analyzed. **The file provides details on all the sequences including in this study. Click here for file

Additional file 27 **Identical virus.** The virus that were excluded for each data set because they are identical. Click here for file

## References

[B1] WHO Dengue: guidelines for diagnosis, treatment, prevention and control 2009 Geneva: World Health Organization 23762963

[B2] Henchal E Putnak J The dengue viruses Clin Microbiol Rev 1990 3 376 396 2224837 10.1128/cmr.3.4.376 PMC358169

[B3] Mackenzie J Gubler D Petersen L Emerging flaviviruses: the spread and resurgence of Japanese encephalitis, West Nile and dengue viruses Nat Med 2004 10 S98 S109 10.1038/nm1144 15577938

[B4] CDC From the Centers for Disease Control and Prevention. Dengue type 3 infection--Nicaragua and Panama, October-November 1994 JAMA 1995 273 840 841 7869544

[B5] Guzmán MG Vázquez S Martínez E Alvarez M Rodríguez R Kourí G de los Reyes J Acevedo F [Dengue in Nicaragua, 1994: reintroduction of serotype 3 in the Americas] Bol Oficina Sanit Panam 1996 121 102 110 8983243

[B6] Usuku S Castillo L Sugimoto C Noguchi Y Yogo Y Kobayashi N Phylogenetic analysis of dengue-3 viruses prevalent in Guatemala during 1996–1998 Arch Virol 2001 146 1381 1390 10.1007/s007050170098 11556713

[B7] Balmaseda A Sandoval E Pérez L Gutiérrez CM Harris E Application of molecular typing techniques in the 1998 dengue epidemic in Nicaragua Am J Trop Med Hyg 1999 61 893 897 10674666 10.4269/ajtmh.1999.61.893

[B8] Peyrefitte CN Couissinier-Paris P Mercier-Perennec V Bessaud M Martial J Kenane N Durand JP Tolou HJ Genetic characterization of newly reintroduced dengue virus type 3 in Martinique (French West Indies) J Clin Microbiol 2003 41 5195 5198 10.1128/JCM.41.11.5195-5198.2003 14605161 PMC262480

[B9] Nogueira R Miagostovich M de Filippis A Pereira M Schatzmayr H Dengue virus type 3 in Rio de Janeiro, Brazil Mem Inst Oswaldo Cruz 2001 96 925 926 10.1590/S0074-02762001000700007 11685256

[B10] Uzcategui NY Comach G Camacho D Salcedo M Cabello de Quintana M Jimenez M Sierra G Cuello de Uzcategui R James WS Turner S Molecular epidemiology of dengue virus type 3 in Venezuela J Gen Virol 2003 84 1569 1575 10.1099/vir.0.18807-0 12771427

[B11] Nogueira R Schatzmayr H de Filippis A dos Santos F da Cunha R Coelho J de Souza L Guimarães F de Araújo E De Simone T Dengue virus type 3, Brazil, 2002 Emerg Infect Dis 2005 11 1376 1381 10.3201/eid1109.041043 16229765 PMC3310608

[B12] Aquino VH Anatriello E Goncalves PF da Silva EV Vasconcelos PFC Vieira DS Batista WC Bobadilla ML Vazquez C Moran M Figueiredo LTM Molecular epidemiology of dengue type 3 virus in Brazil and Paraguay, 2002–2004 Am J Trop Med Hyg 2006 75 710 715 17038699

[B13] Rico-Hesse R Molecular evolution and distribution of dengue viruses type 1 and 2 in nature Virology 1990 174 479 493 10.1016/0042-6822(90)90102-W 2129562

[B14] Chungue E Deubel V Cassar O Laille M Martin P Molecular epidemiology of dengue 3 viruses and genetic relatedness among dengue 3 strains isolated from patients with mild or severe form of dengue fever in French Polynesia J Gen Virol 1993 74 Pt 12 2765 2770 8277284 10.1099/0022-1317-74-12-2765

[B15] Messer W Gubler D Harris E Sivananthan K de Silva A Emergence and global spread of a dengue serotype 3, subtype III virus Emerg Infect Dis 2003 9 800 809 10.3201/eid0907.030038 12899133 PMC3023445

[B16] Kochel T Aguilar P Felices V Comach G Cruz C Alava A Vargas J Olson J Blair P Molecular epidemiology of dengue virus type 3 in Northern South America: 2000–2005 Infect Genet Evol 2008 8 682 688 10.1016/j.meegid.2008.06.008 18674640

[B17] Schreiber M Holmes E Ong S Soh H Liu W Tanner L Aw P Tan H Ng L Leo Y Genomic epidemiology of a dengue virus epidemic in urban Singapore J Virol 2009 83 9 4163 73 10.1128/JVI.02445-08 19211734 PMC2668455

[B18] Sharma S Dash PK Agarwal S Shukla J Parida MM Rao PV Comparative complete genome analysis of dengue virus type 3 circulating in India between 2003–2008 J Gen Virol 2011 92 1595 1600 10.1099/vir.0.030437-0 21411675

[B19] Figueiredo L The Brazilian flaviviruses Microbes Infect 2000 2 1643 1649 10.1016/S1286-4579(00)01320-4 11113383

[B20] Vilela A Figueiredo L dos Santos J Eiras A Bonjardim C Ferreira P Kroon E Dengue virus 3 genotype I in Aedes aegypti mosquitoes and eggs, Brazil, 2005–2006 Emerg Infect Dis 2010 16 989 992 10.3201/eid1606.091000 20507754 PMC3086226

[B21] Barcelos Figueiredo L Batista Cecílio A Portela Ferreira G Paiva Drumond B Germano de Oliveira J Bonjardim C Peregrino Ferreira P Kroon E Dengue virus 3 genotype 1 associated with dengue fever and dengue hemorrhagic fever, Brazil Emerg Infect Dis 2008 14 314 316 10.3201/eid1402.070278 18258129 PMC2600180

[B22] Aquino V Amarilla A Alfonso H Batista W Figueiredo L New genotype of dengue type 3 virus circulating in Brazil and Colombia showed a close relationship to old Asian viruses PLoS One 2009 4 e7299 10.1371/journal.pone.0007299 19823677 PMC2757910

[B23] Araújo J Nogueira R Schatzmayr H Zanotto P Bello G Phylogeography and evolutionary history of dengue virus type 3 Infect Genet Evol 2009 9 716 725 10.1016/j.meegid.2008.10.005 19010450

[B24] Lanciotti R Lewis J Gubler D Trent D Molecular evolution and epidemiology of dengue-3 viruses J Gen Virol 1994 75 Pt 1 65 75 8113741 10.1099/0022-1317-75-1-65

[B25] Chow V Seah C Chan Y Comparative analysis of NS3 sequences of temporally separated dengue 3 virus strains isolated from southeast Asia Intervirology 1994 37 252 258 7698880 10.1159/000150386

[B26] Klungthong C Putnak R Mammen MP Li T Zhang C Molecular genotyping of dengue viruses by phylogenetic analysis of the sequences of individual genes J Virol Methods 2008 154 175 181 10.1016/j.jviromet.2008.07.021 18778736

[B27] Twiddy S Holmes E Rambaut A Inferring the rate and time-scale of dengue virus evolution Mol Biol Evol 2003 20 122 129 10.1093/molbev/msg010 12519914

[B28] Amarilla A de Almeida F Jorge D Alfonso H de Castro-Jorge L Nogueira N Figueiredo L Aquino V Genetic diversity of the E protein of dengue type 3 virus Virol J 2009 6 113 10.1186/1743-422X-6-113 19627608 PMC2720943

[B29] Lewis J Chang G Lanciotti R Kinney R Mayer L Trent D Phylogenetic relationships of dengue-2 viruses Virology 1993 197 216 224 10.1006/viro.1993.1582 8212556

[B30] Lanciotti R Gubler D Trent D Molecular evolution and phylogeny of dengue-4 viruses J Gen Virol 1997 78 Pt 9 2279 2284 9292015 10.1099/0022-1317-78-9-2279

[B31] Goncalvez A Escalante A Pujol F Ludert J Tovar D Salas R Liprandi F Diversity and evolution of the envelope gene of dengue virus type 1 Virology 2002 303 110 119 10.1006/viro.2002.1686 12482662

[B32] Twiddy S Farrar J Vinh Chau N Wills B Gould E Gritsun T Lloyd G Holmes E Phylogenetic relationships and differential selection pressures among genotypes of dengue-2 virus Virology 2002 298 63 72 10.1006/viro.2002.1447 12093174

[B33] Wittke V Robb T Thu H Nisalak A Nimmannitya S Kalayanrooj S Vaughn D Endy T Holmes E Aaskov J Extinction and rapid emergence of strains of dengue 3 virus during an interepidemic period Virology 2002 301 148 156 10.1006/viro.2002.1549 12359455

[B34] Igarashi A Isolation of a Singh’s Aedes albopictus cell clone sensitive to Dengue and Chikungunya viruses J Gen Virol 1978 40 531 544 10.1099/0022-1317-40-3-531 690610

[B35] Poloni T Oliveira A Alfonso H Galvao L Amarilla A Poloni D Figueiredo L Aquino V Detection of dengue virus in saliva and urine by real time RT-PCR Virol J 2010 7 22 10.1186/1743-422X-7-22 20105295 PMC2835670

[B36] Tamura K Peterson D Peterson N Stecher G Nei M Kumar S MEGA5: molecular evolutionary genetics analysis using maximum likelihood, evolutionary distance, and maximum parsimony methods Mol Biol Evol 2011 10 2731 2739 21546353 10.1093/molbev/msr121 PMC3203626

[B37] Hall T BioEdit: a user-friendly biological sequence alignment editor and analysis program for Windows 95/98/NT Nucl Acids Symp Ser 1999 41 95 98

[B38] Xia X Xie Z DAMBE: software package for data analysis in molecular biology and evolution J Hered 2001 92 371 373 10.1093/jhered/92.4.371 11535656

[B39] Larkin M Blackshields G Brown N Chenna R McGettigan P McWilliam H Valentin F Wallace I Wilm A Lopez R Clustal W and Clustal X version 2.0 Bioinformatics 2007 23 2947 2948 10.1093/bioinformatics/btm404 17846036

[B40] Posada D ModelTest Server: a web-based tool for the statistical selection of models of nucleotide substitution online Nucleic Acids Res 2006 34 W700 W703 10.1093/nar/gkl042 16845102 PMC1538795

[B41] Swofford D PAUP*: phylogenetic analysis using parsimony (*and other methods). vol. Version 4.0b10a 1998 Sunderland, Mass: Sinauer Associates

[B42] Nylander JAA MrModelTest v2 2004 Uppsala, Sweden: Evolutionary Biology Center, University of Uppsala

[B43] Ronquist F Huelsenbeck J MrBayes 3: Bayesian phylogenetic inference under mixed models Bioinformatics 2003 19 1572 1574 10.1093/bioinformatics/btg180 12912839

[B44] Grard G Moureau G Charrel RN Lemasson JJ Gonzalez JP Gallian P Gritsun TS Holmes EC Gould EA de Lamballerie X Genetic characterization of tick-borne flaviviruses: new insights into evolution, pathogenetic determinants and taxonomy Virology 2007 361 80 92 10.1016/j.virol.2006.09.015 17169393

