# Supplementary figures and images for: Phylogenetic relationship of dengue virus type 3 isolated in Brazil and Paraguay and global evolutionary divergence dynamics
Source: Virol J. 2012 Jun 20;9:124. doi: 10.1186/1743-422X-9-124 (PMC3494512; doi:10.1186/1743-422X-9-124)

## Slide 1
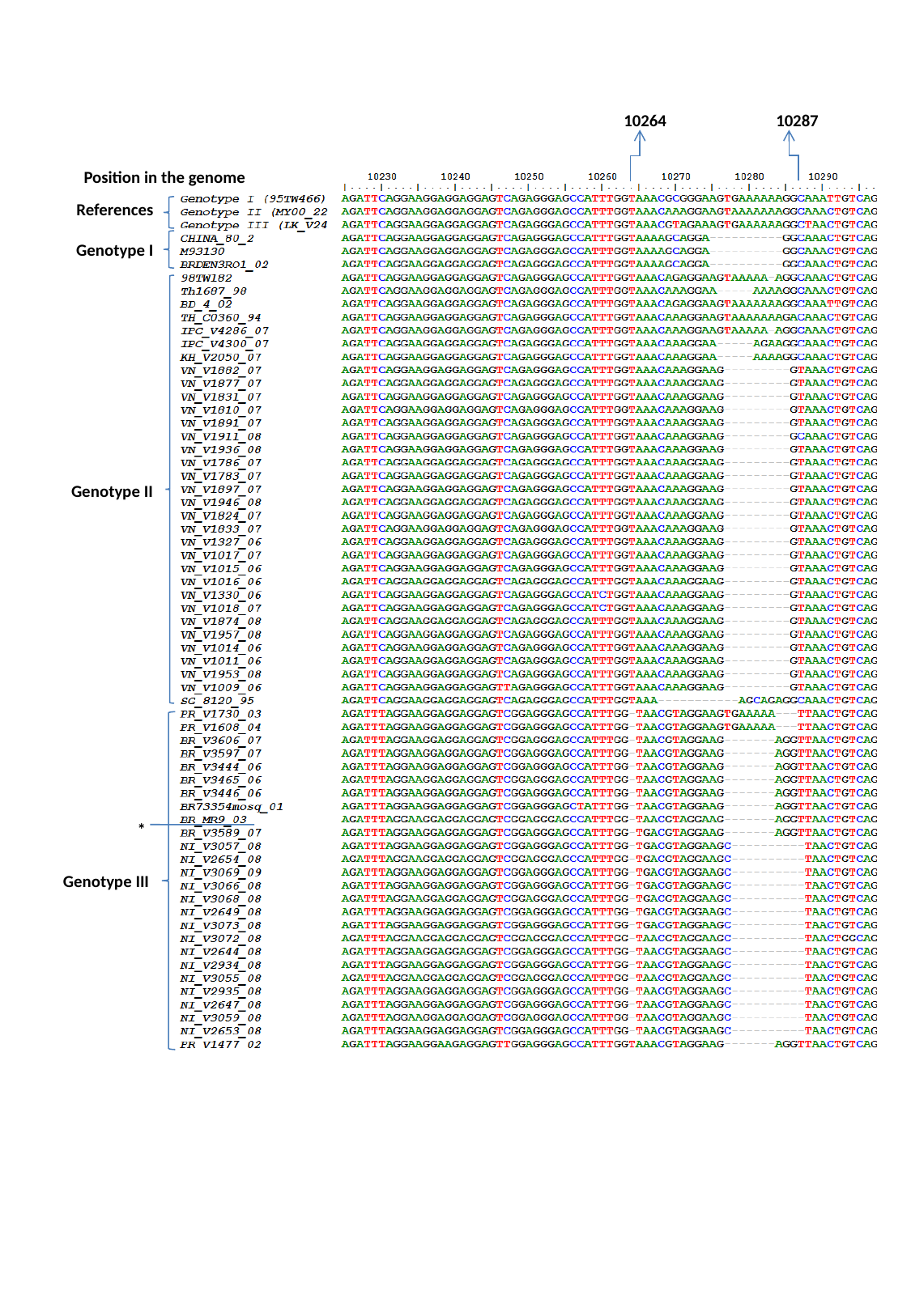

10264
10287
Position in the genome
References
Genotype I
Genotype II
*
Genotype III

Supplement: Additional file 1 — Comparison of the 3′UTR variable region between the position 10,264 to 1,287 of several isolates belongs to genotypes I, II and III. Sequence of isolates from genotypes I, II and III, which showed differences in 3′UTR region. [file 1743-422X-9-124-S1.ppt]

## Slide 1
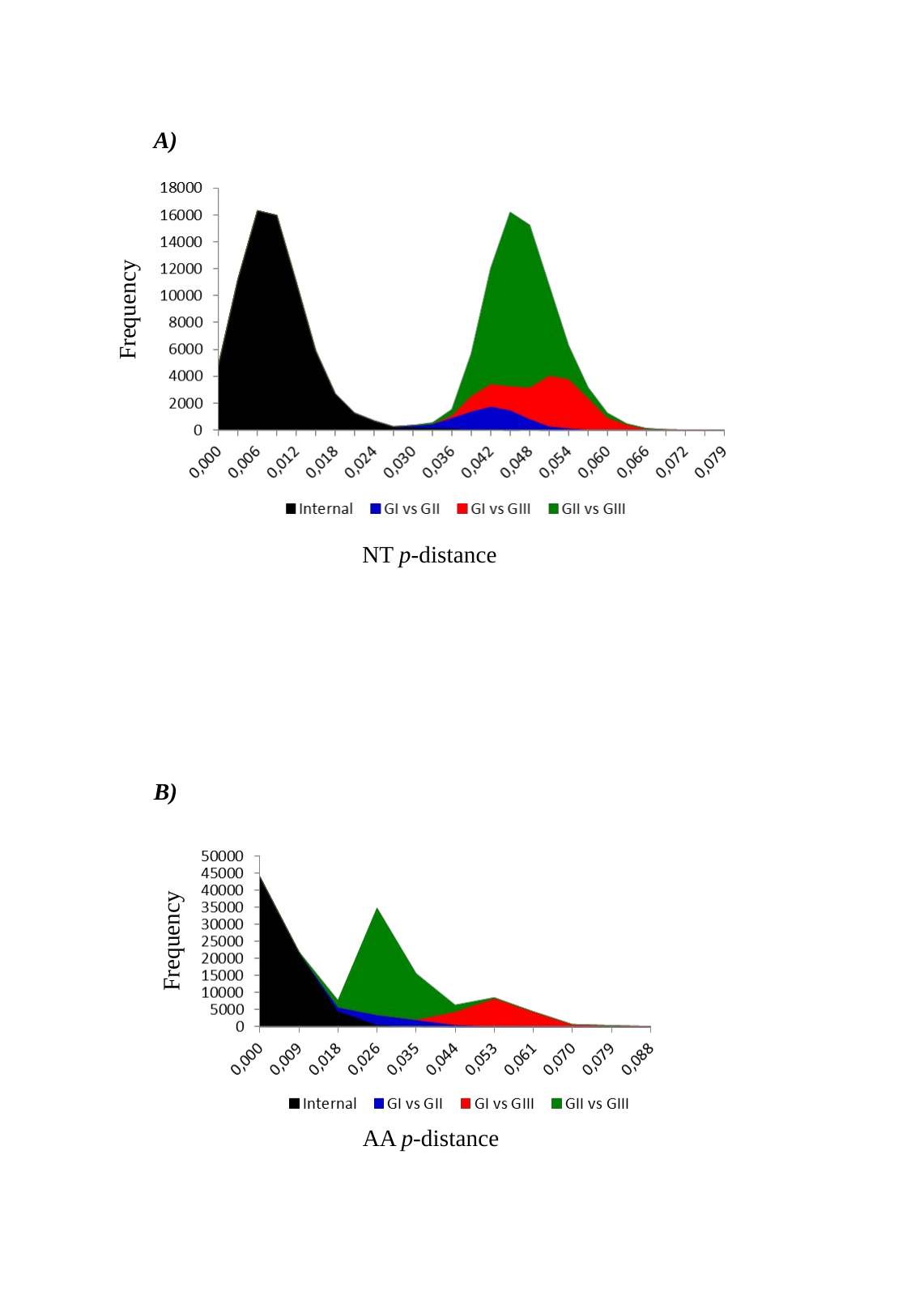

A)
Frequency
NT p-distance
B)
Frequency
AA p-distance

Supplement: Additional file 5 — Distribution of NT (A) and AA (B) p-distances between different genotypes based on the C protein gene of 537 DENV-3. NT: nucleotides; AA: amino acids. [file 1743-422X-9-124-S5.ppt]

## Slide 1
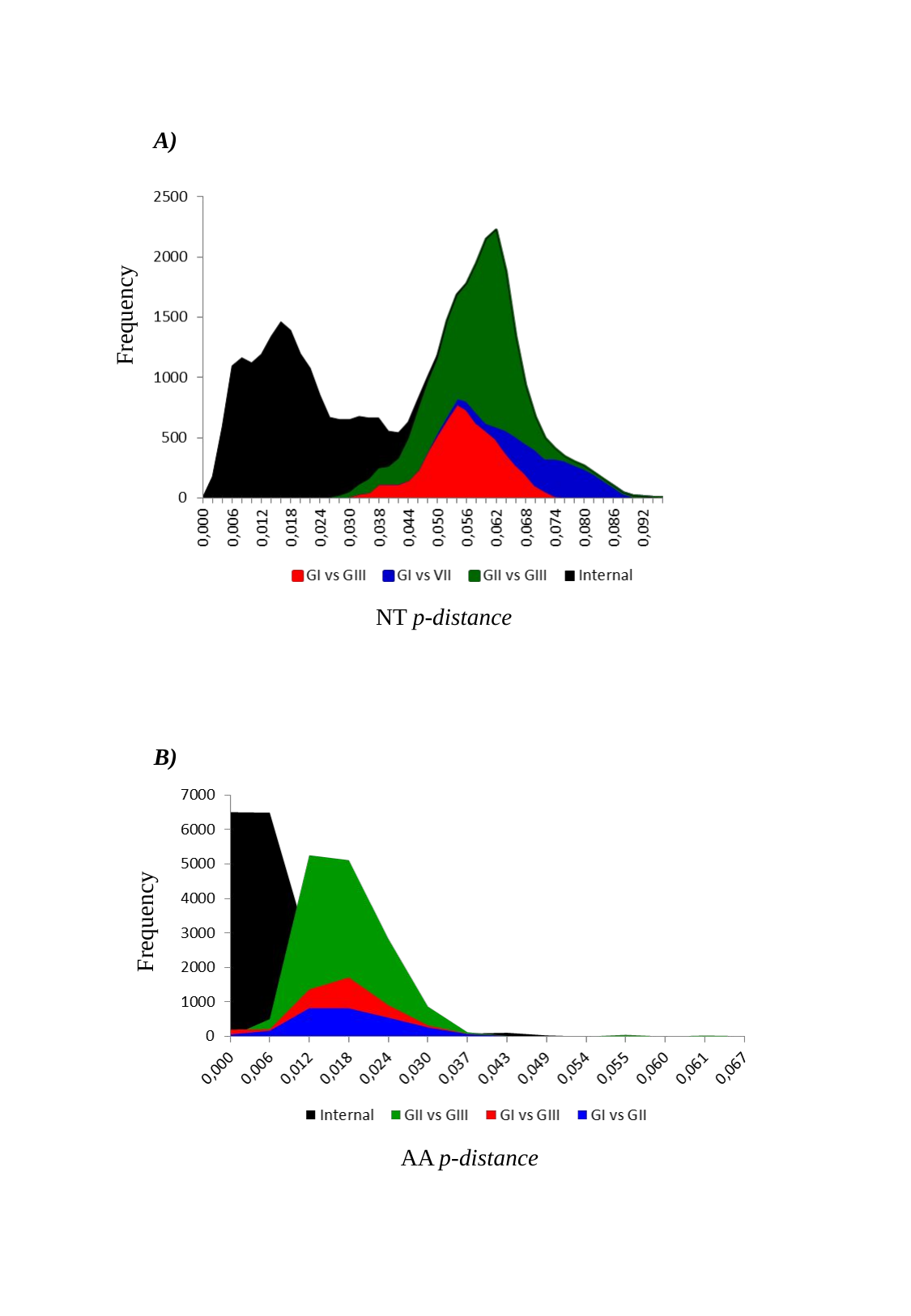

A)
Frequency
NT p-distance
B)
Frequency
AA p-distance

Supplement: Additional file 6 — Distribution of NT (A) and AA (B) p-distances between different genotypes based on the prM protein gene of 537 DENV-3. NT: nucleotides; AA: amino acids. [file 1743-422X-9-124-S6.ppt]

## Slide 1
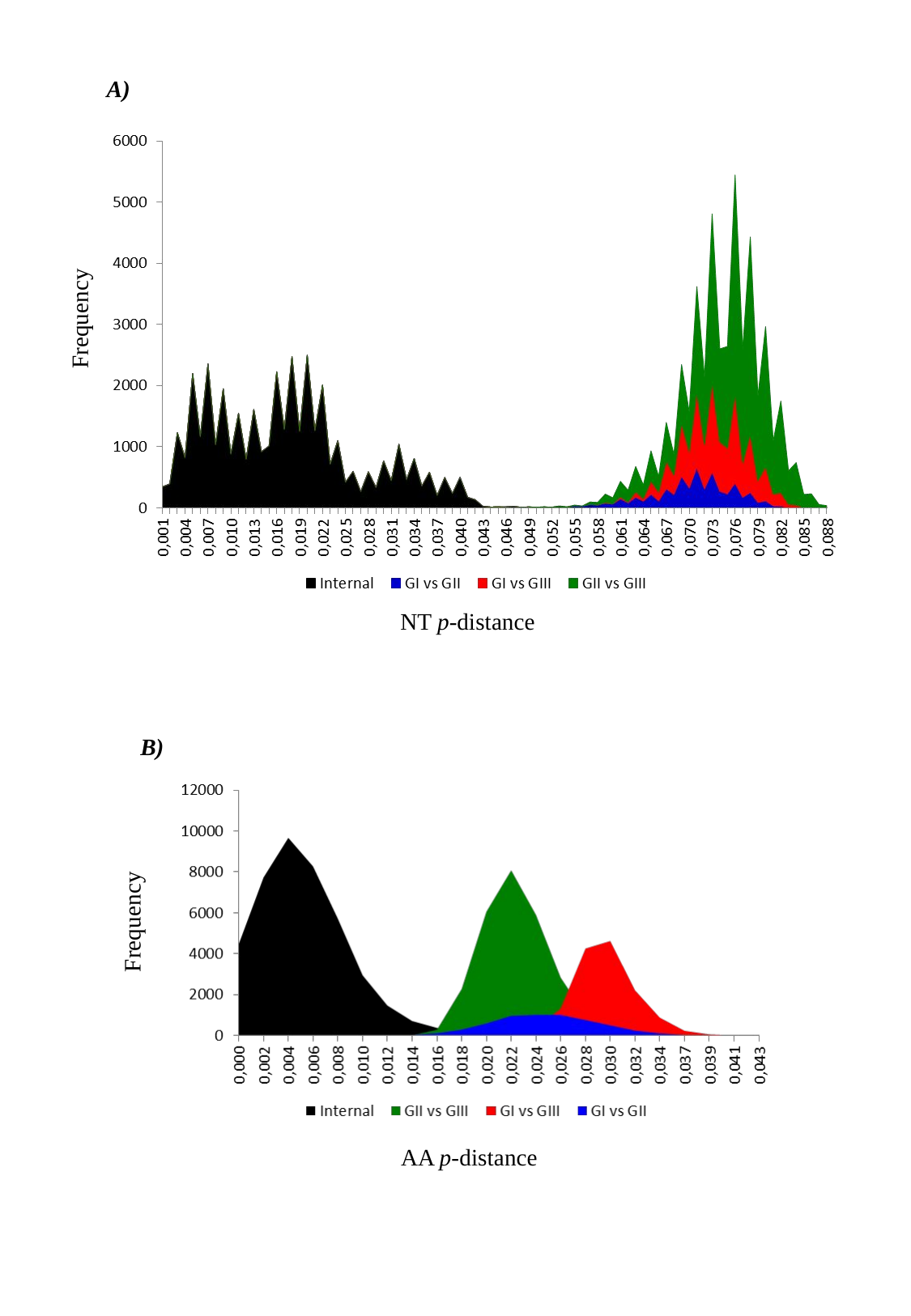

A)
Frequency
NT p-distance
B)
Frequency
AA p-distance

Supplement: Additional file 7 — Distribution of NT (A) and AA (B) p-distances between different genotypes based on the E protein gene of 424 DENV-3. NT: nucleotides; AA: amino acids. [file 1743-422X-9-124-S7.ppt]

## Slide 1
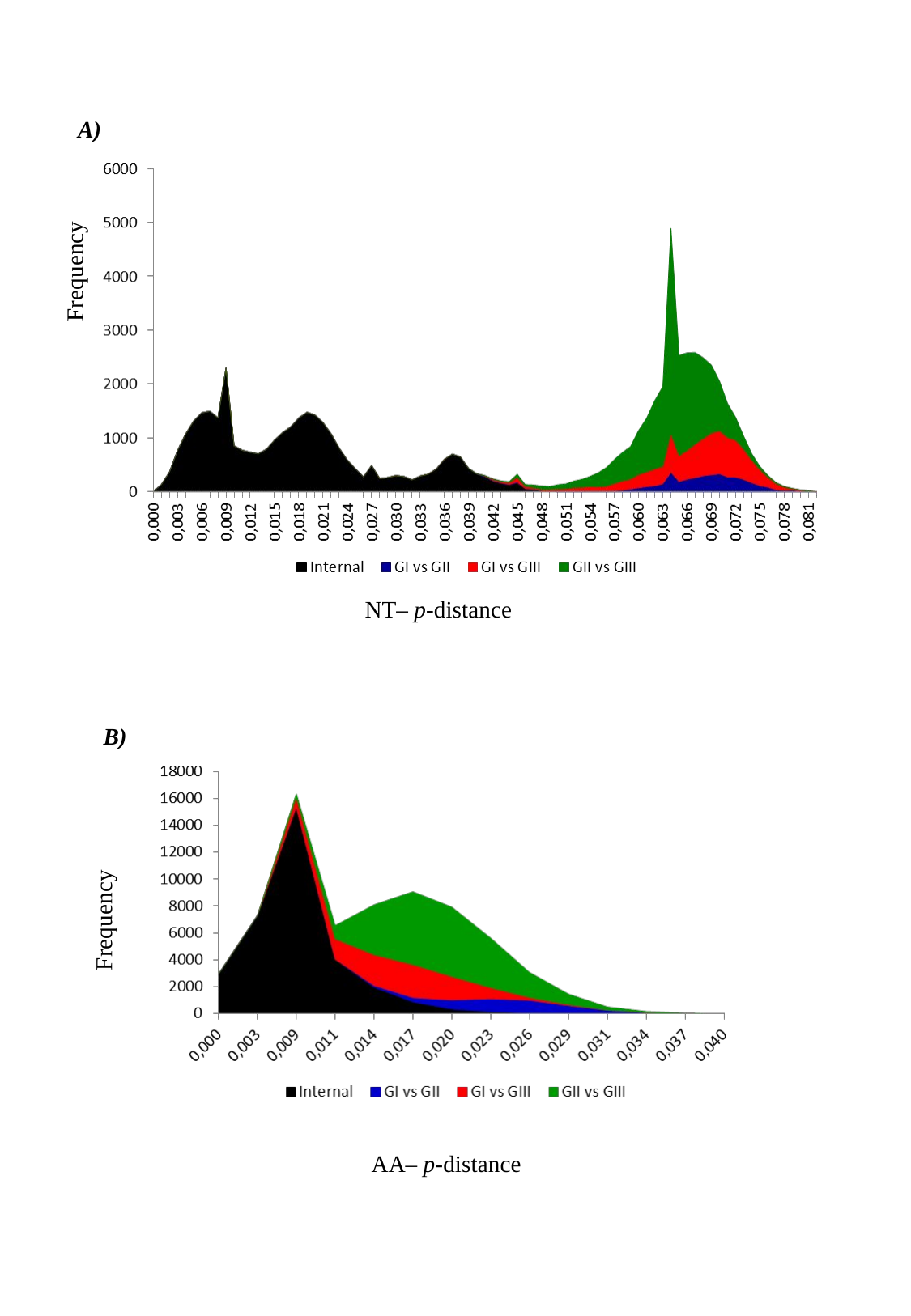

A)
Frequency
NT– p-distance
B)
Frequency
AA– p-distance

Supplement: Additional file 8 — Distribution of NT (A) and AA (B) p-distances between different genotypes based on the NS1 protein gene of 372 DENV-3. NT: nucleotides; AA: amino acids. [file 1743-422X-9-124-S8.ppt]

## Slide 1
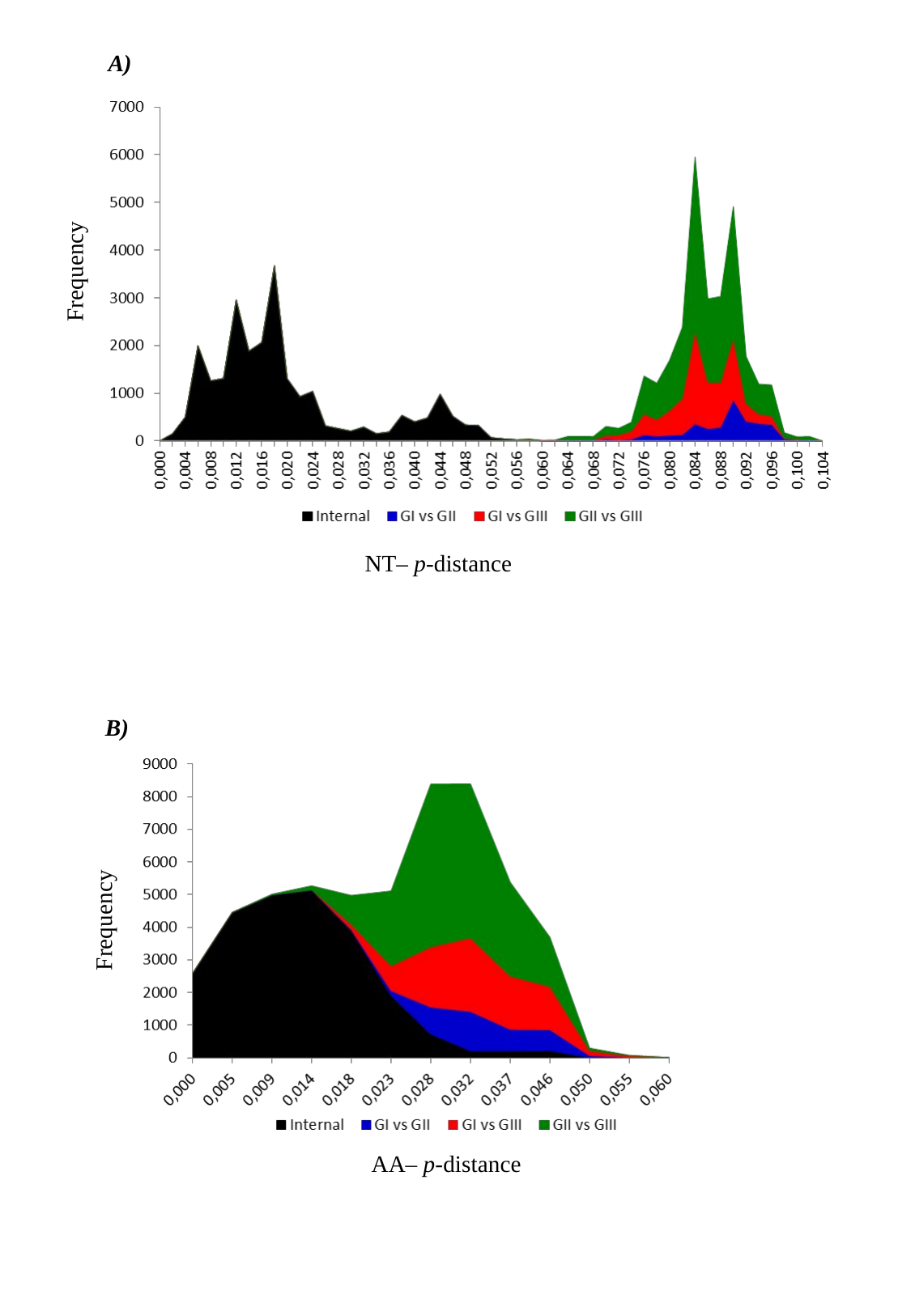

A)
Frequency
NT– p-distance
B)
Frequency
AA– p-distance

Supplement: Additional file 9 — Distribution of NT (A) and AA (B) p-distances between different genotypes based on the NS2A protein gene of 328 DENV-3. NT: nucleotides; AA: amino acids. [file 1743-422X-9-124-S9.ppt]

## Slide 1
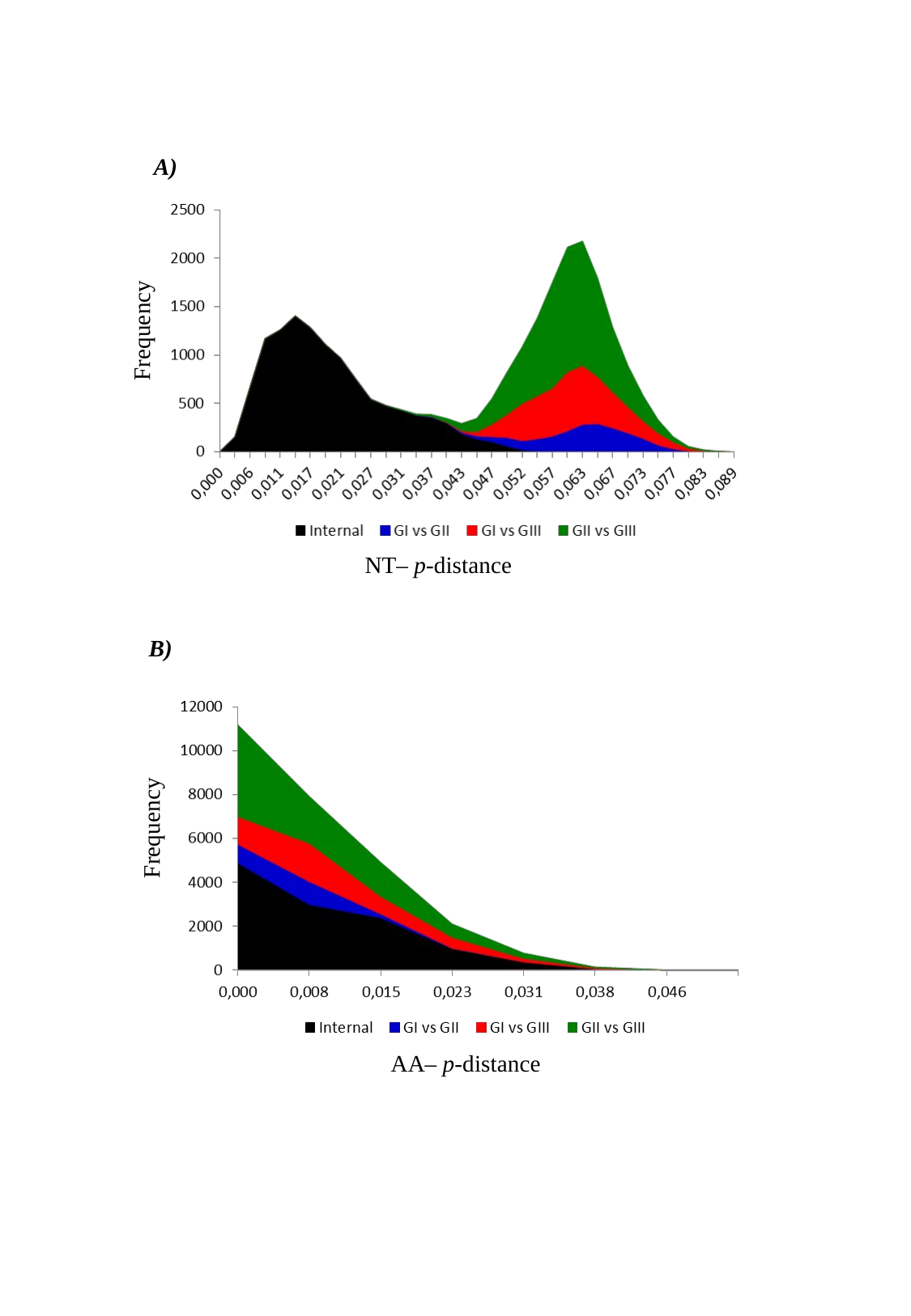

A)
Frequency
NT– p-distance
B)
Frequency
AA– p-distance

Supplement: Additional file 10 — Distribution of NT (A) and AA (B) p-distances between different genotypes based on the NS2B protein gene of 233 DENV-3. NT: nucleotides; AA: amino acids. [file 1743-422X-9-124-S10.ppt]

## Slide 1
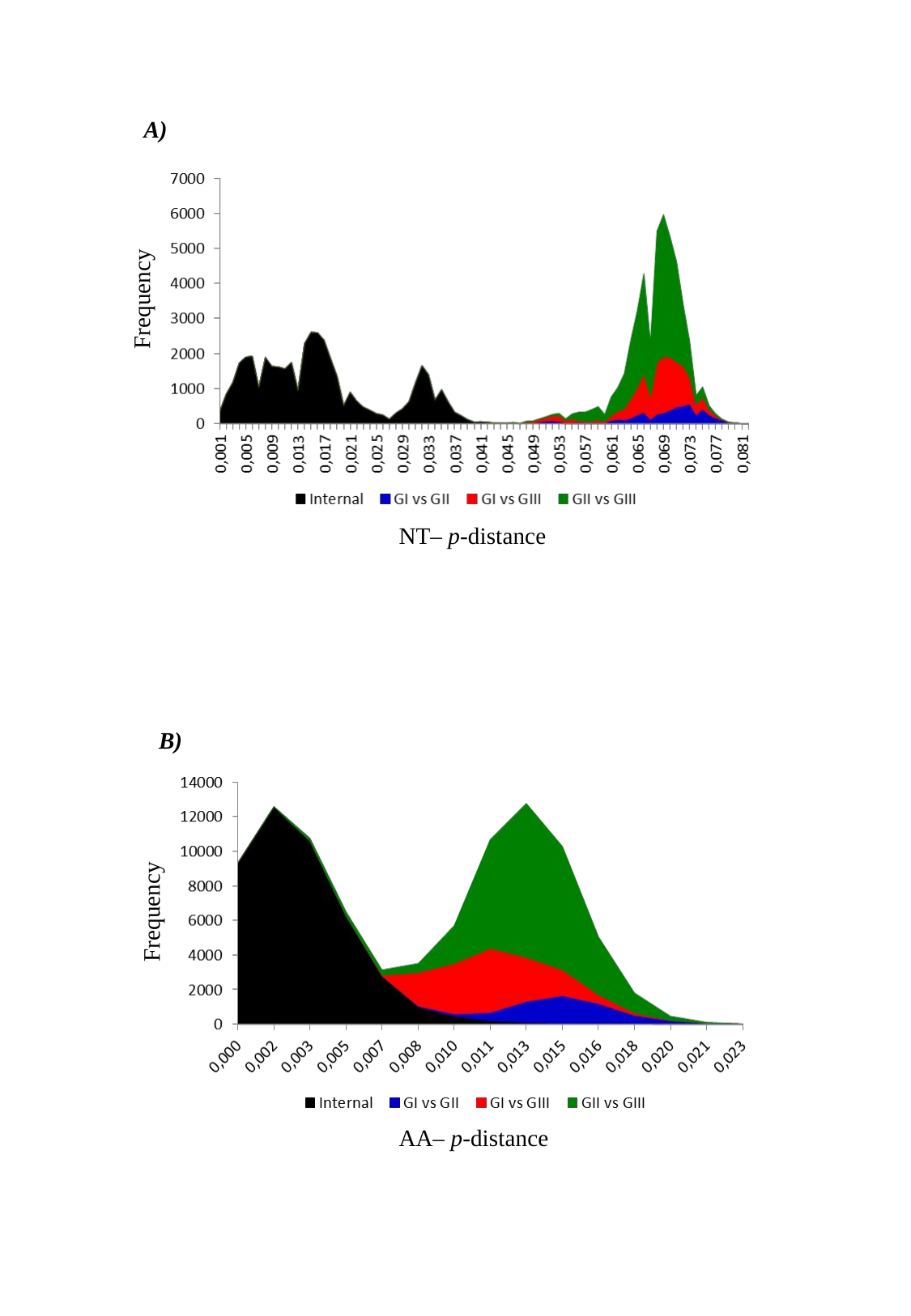

A)
Frequency
NT– p-distance
B)
Frequency
AA– p-distance

Supplement: Additional file 11 — Distribution of NT (A) and AA (B) p-distances between different genotypes based on the NS3 protein gene of 431 DENV-3. NT: nucleotides; AA: amino acids. [file 1743-422X-9-124-S11.ppt]

## Slide 1
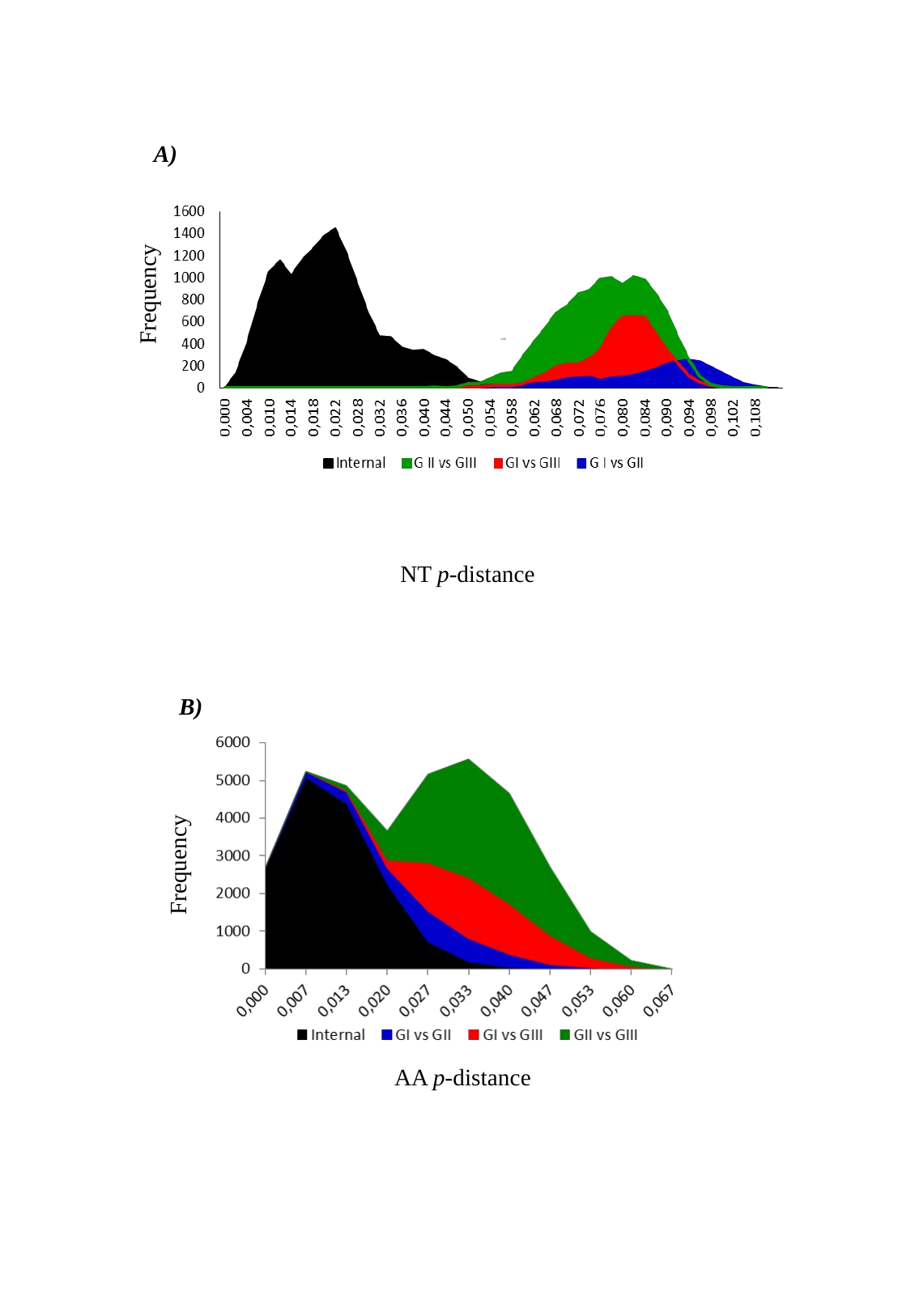

A)
Frequency
NT p-distance
B)
Frequency
AA p-distance

Supplement: Additional file 12 — Distribution of NT (A) and AA (B) p-distances between different genotypes based on the NS4A protein gene of 268 DENV-3. NT: nucleotides; AA: amino acids. [file 1743-422X-9-124-S12.ppt]

## Slide 1
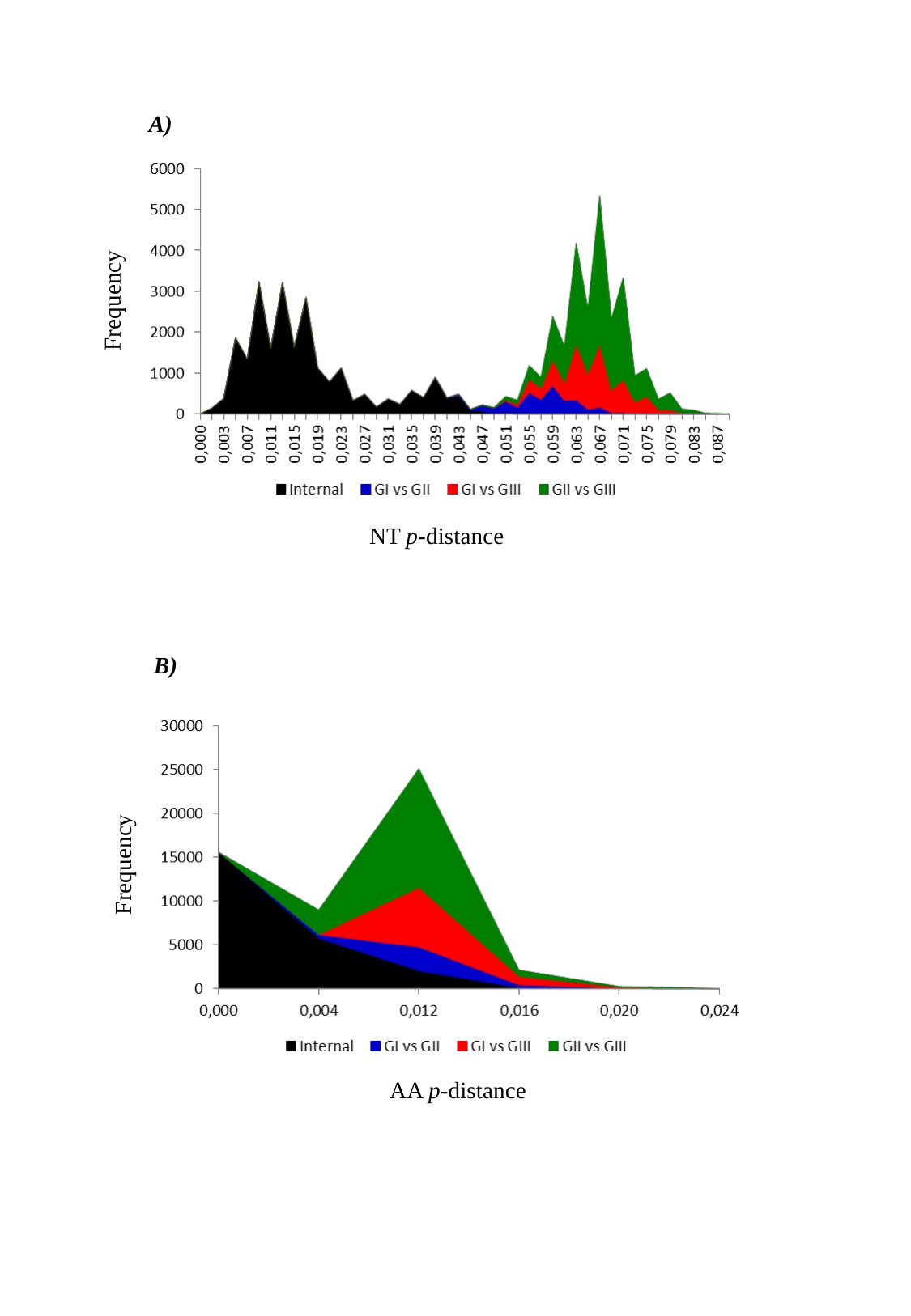

A)
Frequency
NT p-distance
B)
Frequency
AA p-distance

Supplement: Additional file 13 — Distribution of NT (A) and AA (B) p-distances between different genotypes based on the NS4B protein gene of 323 DENV-3. NT: nucleotides; AA: amino acids. [file 1743-422X-9-124-S13.ppt]

## Slide 1
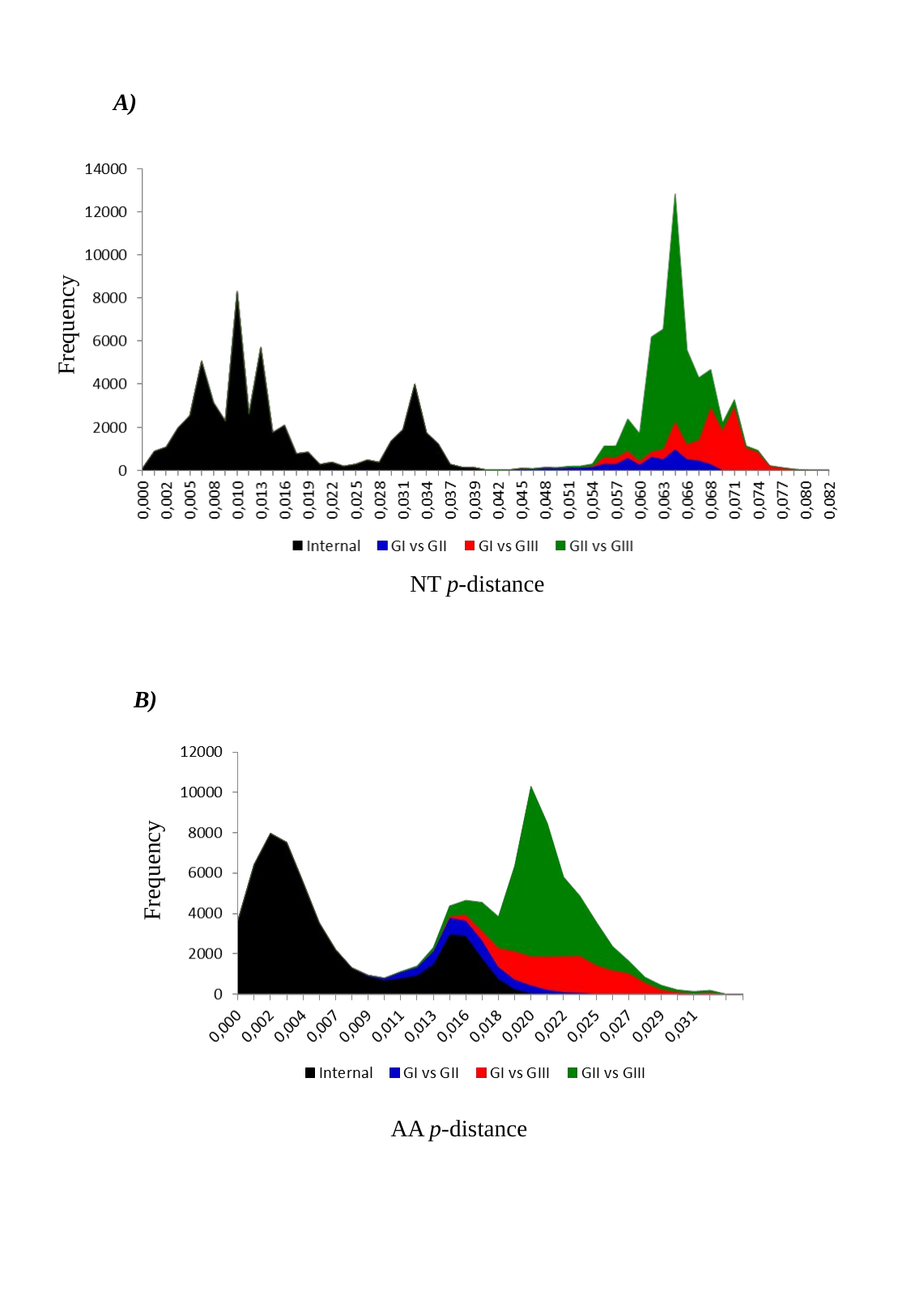

A)
Frequency
NT p-distance
Frequency
AA p-distance
B)

Supplement: Additional file 14 — Distribution of NT (A) and AA (B) p-distances between different genotypes based on the NS5 protein gene of 537 DENV-3. NT: nucleotides; AA: amino acids. [file 1743-422X-9-124-S14.ppt]

## Slide 1
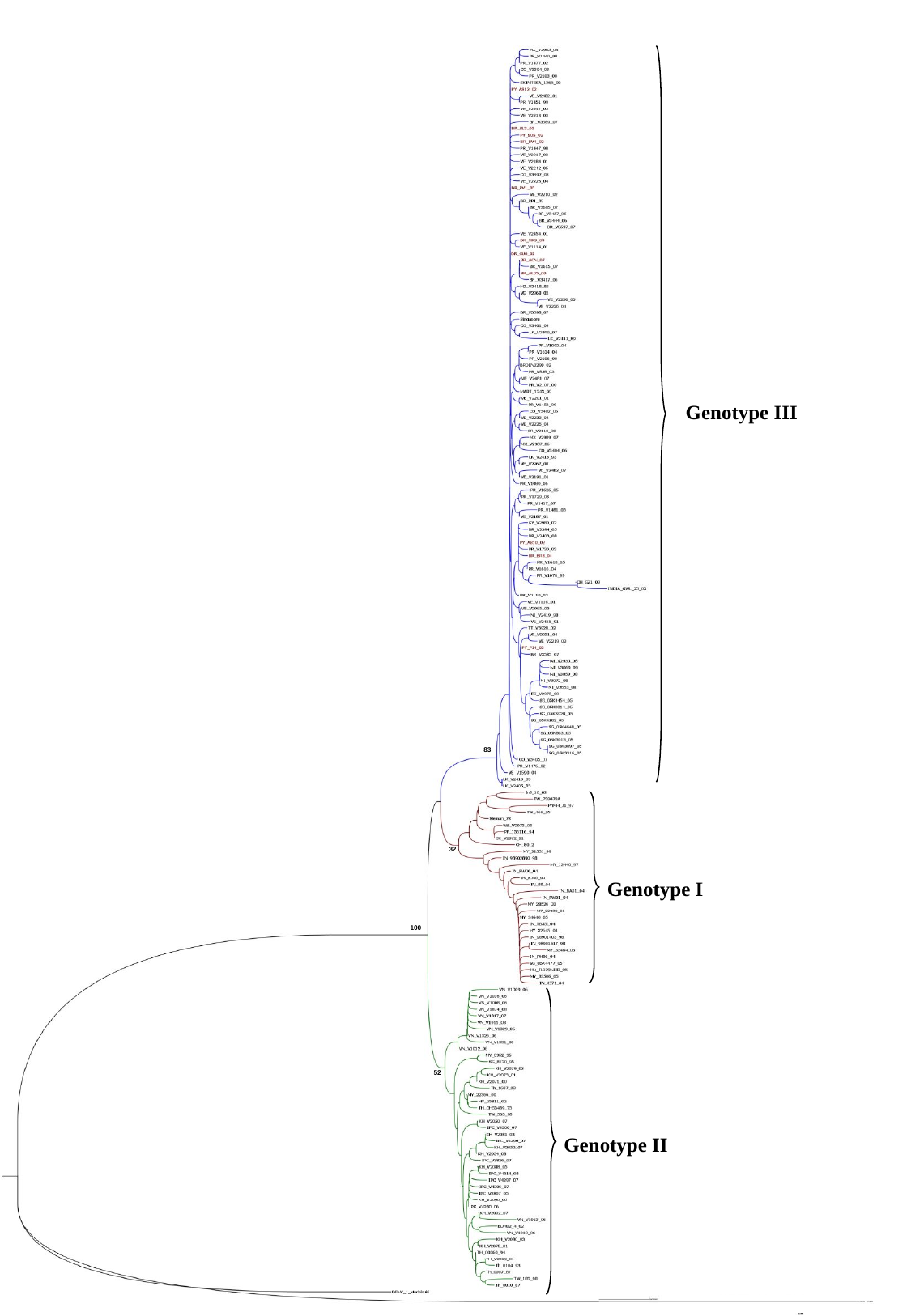

Genotype III
Genotype I
Genotype II
83
32
100
52

Supplement: Additional file 15 — Neighbor-joining phylogenetic trees based on C gene derived from 537 global samples of the DENV-3 inferred with MEGA 5 program. The bootstrap are indicated at important nodes. The best-fit model of nucleotide substitution for phylogenetic reconstruction used was TrN + G model with gamma-distributed rate variation (G = 1.2). Branch lengths are proportional to percentage of divergence. [file 1743-422X-9-124-S15.ppt]

## Slide 1
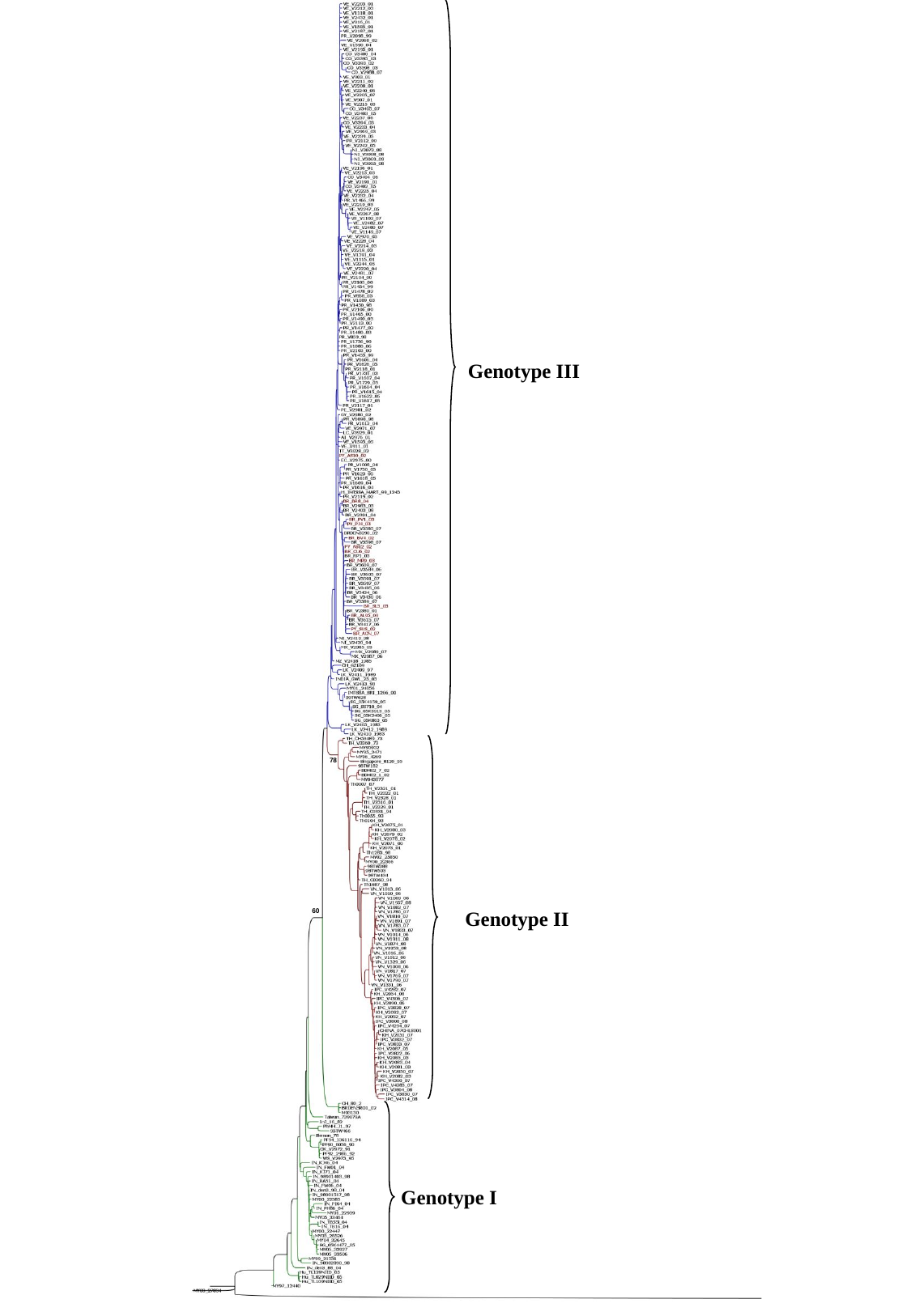

Genotype III
78
Genotype II
60
Genotype I

Supplement: Additional file 16 — Neighbor-joining phylogenetic trees based on prM gene derived from 283 global samples of the DENV-3 inferred with MEGA 5 program. The bootstrap are indicated at important nodes. The best-fit model of nucleotide substitution for phylogenetic reconstruction used was TrN + G model with gamma-distributed rate variation (G = 1.2). Branch lengths are proportional to percentage of divergence. [file 1743-422X-9-124-S16.ppt]

## Slide 1
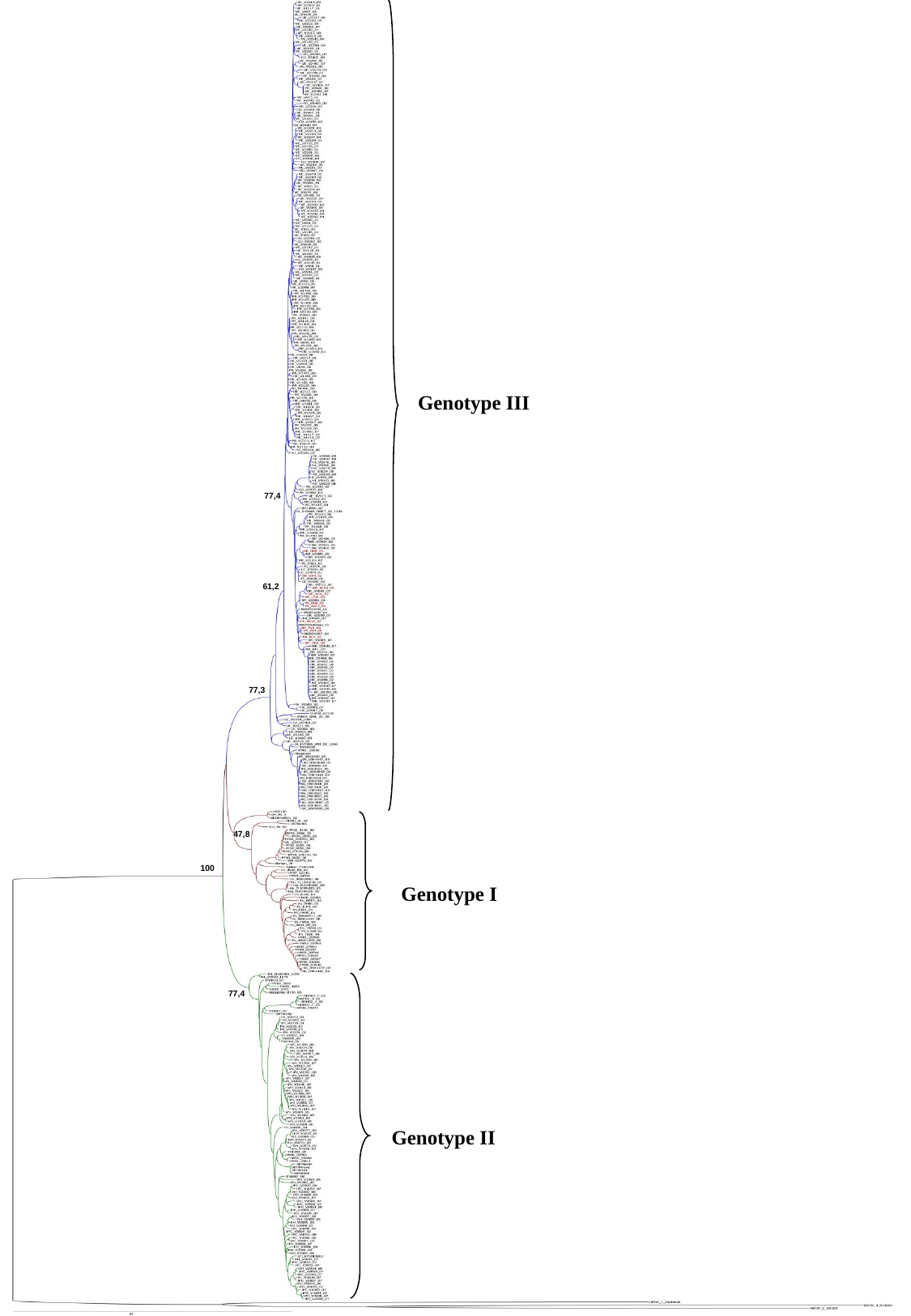

Genotype III
77,4
61,2
77,3
47,8
100
Genotype I
77,4
Genotype II

Supplement: Additional file 17 — Neighbor-joining phylogenetic trees based on E gene derived from 424 global samples of the DENV-3 inferred with MEGA 5 program. The bootstrap are indicated at important nodes. The best-fit model of nucleotide substitution for phylogenetic reconstruction used was TrN + G model with gamma-distributed rate variation (G = 1.2). Branch lengths are proportional to percentage of divergence. [file 1743-422X-9-124-S17.ppt]

## Slide 1
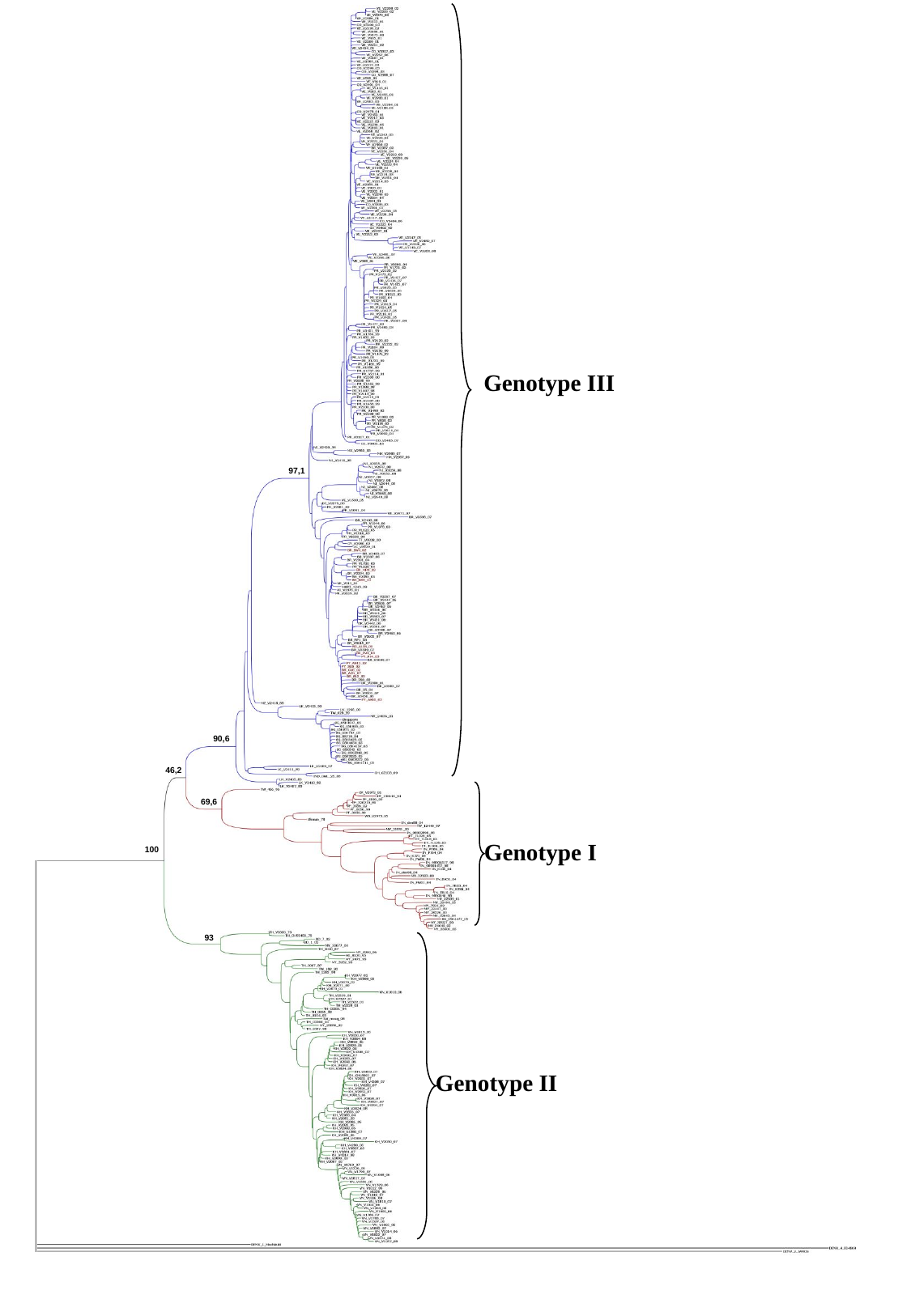

Genotype III
97,1
90,6
46,2
69,6
Genotype I
100
93
Genotype II

Supplement: Additional file 18 — Neighbor-joining phylogenetic trees based on NS1 gene derived from 372 global samples of the DENV-3 inferred with MEGA 5 program. The bootstrap are indicated at important nodes. The best-fit model of nucleotide substitution for phylogenetic reconstruction used was TrN + G model with gamma-distributed rate variation (G = 1.2). Branch lengths are proportional to percentage of divergence. [file 1743-422X-9-124-S18.ppt]

## Slide 1
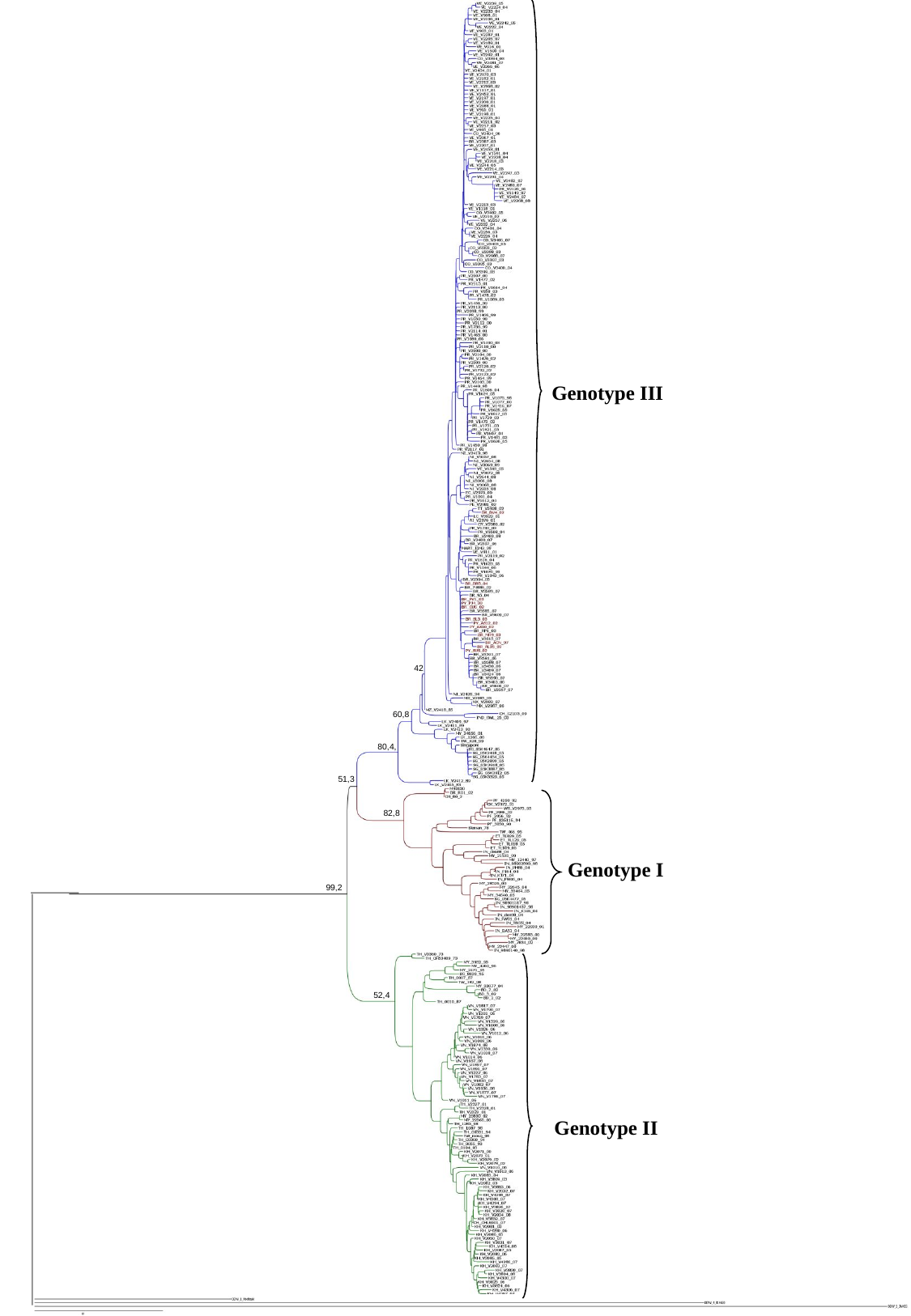

Genotype III
Genotype I
Genotype II
42
60,8
80,4,
51,3
82,8
99,2
52,4

Supplement: Additional file 19 — Neighbor-joining phylogenetic trees based on NS2A gene derived from 328 global samples of the DENV-3 inferred with MEGA 5 program. The bootstrap are indicated at important nodes. The best-fit model of nucleotide substitution for phylogenetic reconstruction used was TrN + G model with gamma-distributed rate variation (G = 1.2). Branch lengths are proportional to percentage of divergence. [file 1743-422X-9-124-S19.ppt]

## Slide 1
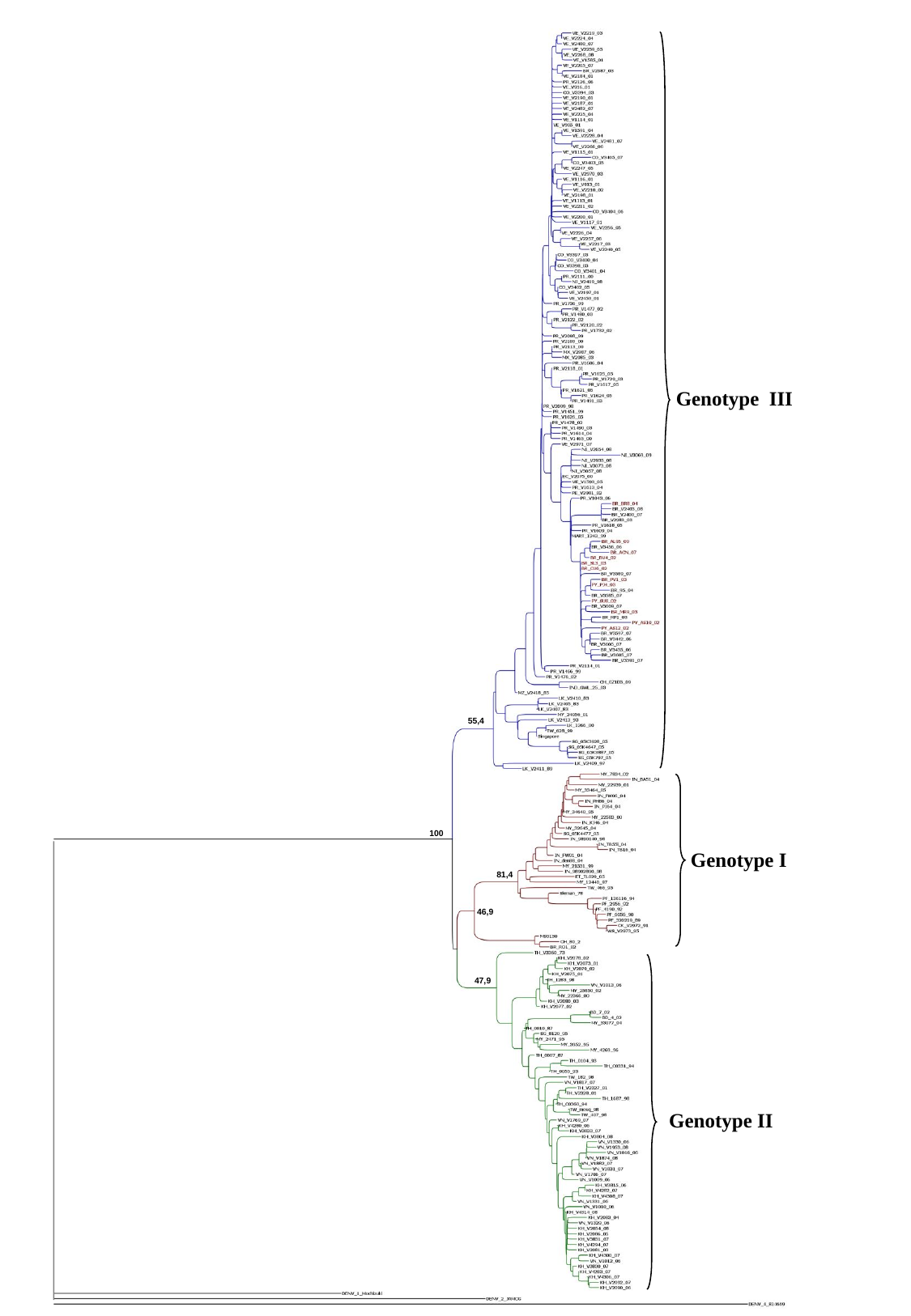

Genotype III
Genotype I
Genotype II
55,4
100
81,4
46,9
47,9

Supplement: Additional file 20 — Neighbor-joining phylogenetic trees based on NS2B gene derived from 233 global samples of the DENV-3 inferred with MEGA 5 program. The bootstrap are indicated at important nodes. The best-fit model of nucleotide substitution for phylogenetic reconstruction used was TrN + G model with gamma-distributed rate variation (G = 1.2). Branch lengths are proportional to percentage of divergence. [file 1743-422X-9-124-S20.ppt]

## Slide 1
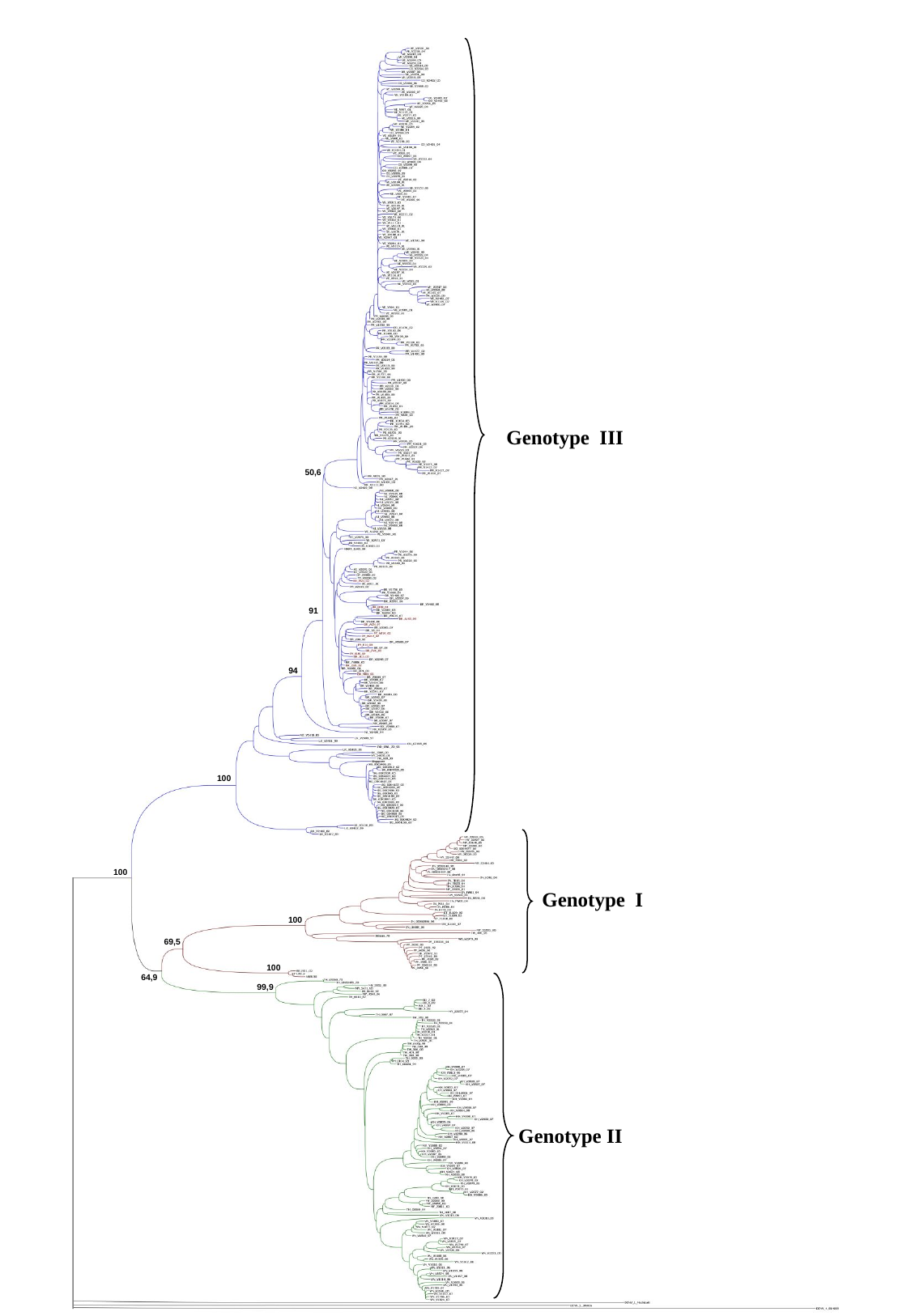

Genotype I
Genotype II
50,6
91
94
100
100
100
69,5
100
64,9
99,9
Genotype III

Supplement: Additional file 21 — Neighbor-joining phylogenetic trees based on NS3 gene derived from 431 global samples of the DENV-3 inferred with MEGA 5 program. The bootstrap are indicated at important nodes. The best-fit model of nucleotide substitution for phylogenetic reconstruction used was TrN + G model with gamma-distributed rate variation (G = 1.2). Branch lengths are proportional to percentage of divergence. [file 1743-422X-9-124-S21.ppt]

## Slide 1
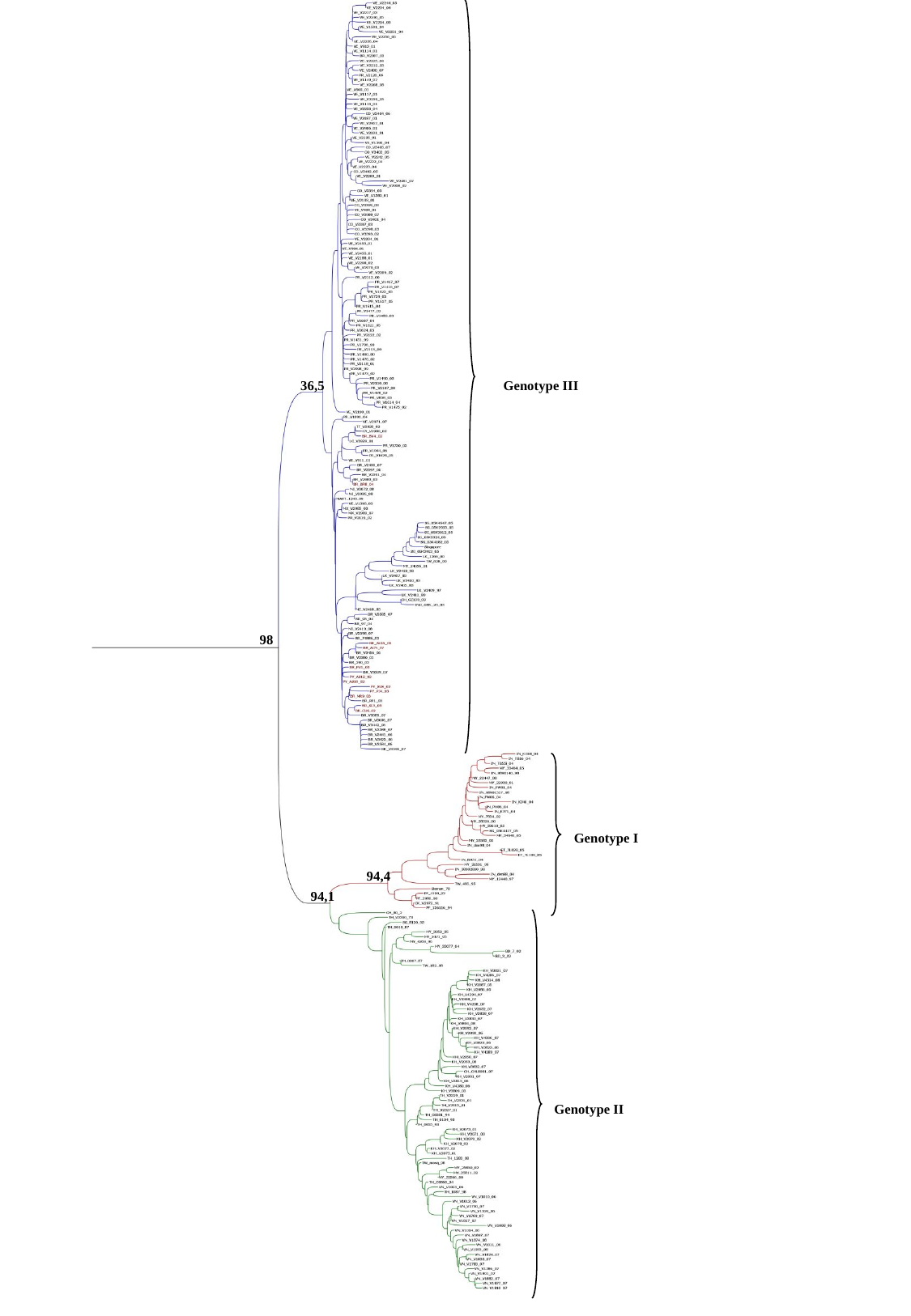

Genotype III
Genotype I
Genotype II
36,5
98
94,4
94,1

Supplement: Additional file 22 — Neighbor-joining phylogenetic trees based on NS4A gene derived from 268 global samples of the DENV-3 inferred with MEGA 5 program. The bootstrap are indicated at important nodes. The best-fit model of nucleotide substitution for phylogenetic reconstruction used was TrN + G model with gamma-distributed rate variation (G = 1.2). Branch lengths are proportional to percentage of divergence. [file 1743-422X-9-124-S22.ppt]

## Slide 1
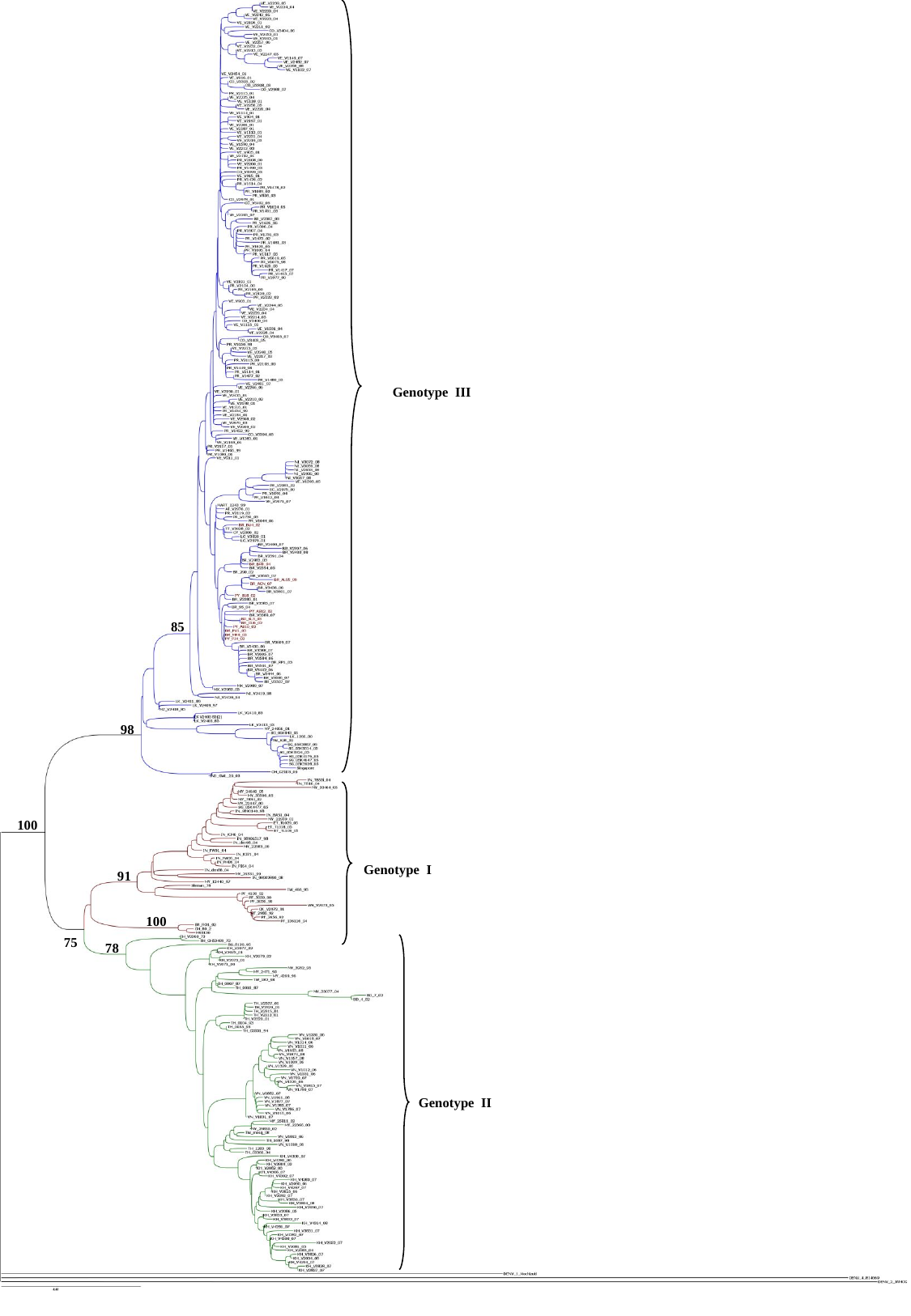

Genotype III
Genotype I
Genotype II
85
98
100
91
100
75
78

Supplement: Additional file 23 — Neighbor-joining phylogenetic trees based on NS4B gene derived from 323 global samples of the DENV-3 inferred with MEGA 5 program. The bootstrap are indicated at important nodes. The best-fit model of nucleotide substitution for phylogenetic reconstruction used was TrN + G model with gamma-distributed rate variation (G = 1.2). Branch lengths are proportional to percentage of divergence. [file 1743-422X-9-124-S23.ppt]

## Slide 1
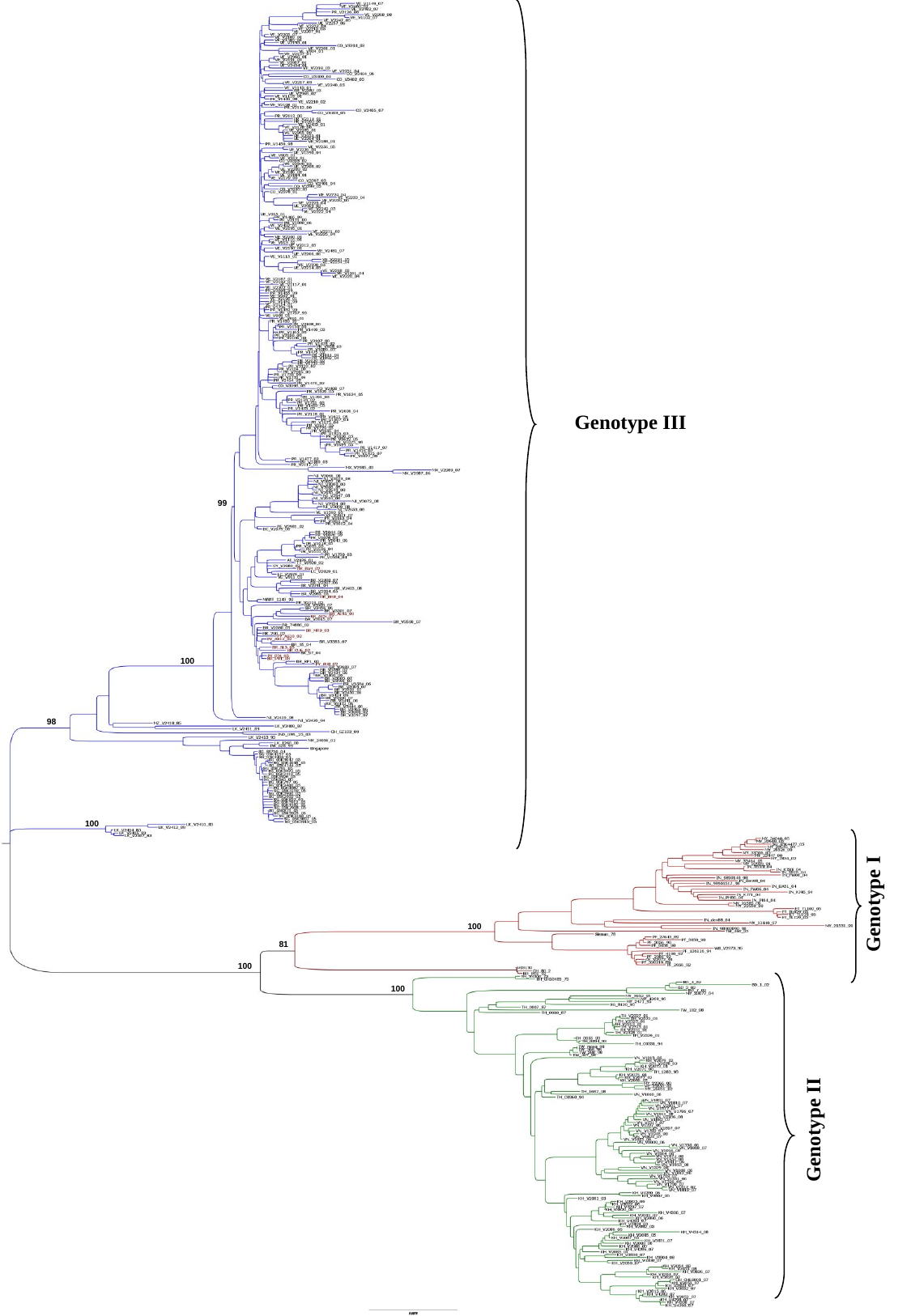

Genotype III
99
100
98
100
Genotype I
100
81
100
100
Genotype II

Supplement: Additional file 24 — Neighbor-joining phylogenetic trees based on NS5 gene derived from 464 global samples of the DENV-3 inferred with MEGA 5 program. The bootstrap are indicated at important nodes. The best-fit model of nucleotide substitution for phylogenetic reconstruction used was TrN + G model with gamma-distributed rate variation (G = 1.2). Branch lengths are proportional to percentage of divergence. [file 1743-422X-9-124-S24.ppt]
