# Supplementary material for: Phylogenetic relationship of dengue virus type 3 isolated in Brazil and Paraguay and global evolutionary divergence dynamics
Source: Virol J. 2012 Jun 20;9:124. doi: 10.1186/1743-422X-9-124 (PMC3494512; doi:10.1186/1743-422X-9-124)
Supplement: Additional file 2 — Motifs of amino acids for the genotype III. The file provides details on amino acid substitutions present within each genetic group of genotype III. [file 1743-422X-9-124-S2.doc]

| **Genotype III** | | | | | | | | |
| --- | --- | --- | --- | --- | --- | --- | --- | --- |
| **Positions** | **Lineages** | | |  | **Sub-lineages (Lineage III)** | | | |
|  | **I** | **II** | **III** |  | **A** | **B** | **C** | **D** |
| 86/prM | H | H | H/R |  | H | R | H | H |
| 124/E | S | P | P |  | P | P | P | P |
| 329/E | A | A | A/V |  | A | A | V | V |
| 128/NS1 | I | T | T |  | T | T | T | T |
| 217/NS1 | F | L | L |  | L | L | L | L |
| 256/NS1 | Y | Y | H/Y |  | H/Y | H | H | Y |
| 290/NS1 | N | S | N |  | N | N | N | N |
| 350/NS1 | A | V | V |  | V | V | V | V |
| 150/NS2A | V/I | V | I |  | I | I | I | I |
| 158/NS2A | M | I | I/M |  | M | M | I | I |
| 195/NS2A | T | T | A/T |  | A | A | A | T |
| 109/NS2B | I | I | I/V |  | V | V | I | I |
| 31/NS3 | L | F | F |  | F | F | F | F |
| 60/NS3 | H | H | Y |  | Y | Y | Y | Y |
| 185/NS3 | R | K | K |  | K | K | K | K |
| 39/NS4a | R | R | K/R |  | R | R | R | K |
| 100/NS4a | D | V | I |  | I | I | I | I |
| 50/NS5 | T | T | I/T |  | T | I | I | I |
| 230/NS5 | S | S | A |  | S | S | S | S |
| 253/NS5 | R | K | K |  | K | K | K | K |
| 270/NS5 | I | T | T |  | T | T | T | T |
| 365/NS5 | P | P | S |  | S | S | S | S |
| 371/NS5 | K | K | R |  | R | R | R | R |
| 374/NS5 | E | E | G/E |  | G | G/E | G | G |
| 389/NS5 | R | R | K |  | K | K | K | K |
| 429/NS5 | E | E | D |  | D | D | D | D |
| 553/NS5 | T | I | T |  | T | T | T | T |
| 619/NS5 | V | V | I |  | I | I | I | I |
| 629/NS5 | S | T | S |  | S | S | S | S |
| 639/NS5 | L | L | P |  | P | P | P | P |
| 820/NS5 | D | E | D |  | D | D | D | D |
| 895/NS5 | S | L | S |  | S | S | S | S |
