# Supplementary material for: Phylogenetic relationship of dengue virus type 3 isolated in Brazil and Paraguay and global evolutionary divergence dynamics
Source: Virol J. 2012 Jun 20;9:124. doi: 10.1186/1743-422X-9-124 (PMC3494512; doi:10.1186/1743-422X-9-124)
Supplement: Additional file 3 — Motifs of amino acids for the genotype I. The file provides details on amino acid substitutions present within each genetic group of genotype I. [file 1743-422X-9-124-S3.doc]

| **Genotype I** | | | | | | | | | |
| --- | --- | --- | --- | --- | --- | --- | --- | --- | --- |
| **Position** |  | **Lineages** |  | **Sub-lineages (Lineage II)** | |  | **Groups (Sub-lineage II)** | | |
|  | **I** | **II** |  | **I** | **II** |  | **A** | **B** | **C** |
| 82/C | K | R |  | R | R |  | R | R | R |
| 97/C | K | R |  | R | R |  | R | R | R |
| 55/prM | H | L |  | L | L |  | L | L | L |
| 128/prM | L | F |  | F | F |  | F | F | F |
| 68/E | I | V |  | V | V |  | V | V | V |
| 164/E | S | P |  | P | P |  | P | P | P |
| 169/E | A | V |  | V | V |  | V | V | V |
| 172/E | I | I |  | I | I/V |  | I | V | I |
| 231/E | R | K |  | K | K |  | K | K | K |
| 303/E | T | A |  | A | A |  | A | A | A |
| 355/E | V | V/I |  | I | V |  | V | V | V |
| 391/E | R | K |  | K | K |  | K | K | K |
| 479/E | A | V |  | V | V |  | V | V | V |
| 489/E | V | A |  | A | A |  | A | A | A |
| 93/NS1 | I | I/V |  | V | I |  | I | I | I |
| 94/NS1 | T | I |  | I | I |  | I | I | I |
| 128/NS1 | S | N |  | N | N |  | N | N | N |
| 173/NS1 | V | M |  | M | M |  | M | M | M |
| 256/NS1 | H | Y |  | Y | Y |  | Y | Y | Y |
| 287/NS1 | S | T |  | T | T |  | T | T | T |
| 350/NS1 | A | V |  | V | V |  | V | V | V |
| 5/NS2A | V | M |  | M | M |  | M | M | M |
| 36/NS2A | V | V/A |  | A | V |  | V | V | V |
| 112/NS2A | A | T |  | T | T |  | T | T | T |
| 115/NS2A | R | Q |  | Q | Q |  | Q | Q | Q |
| 174/NS2A | G | G/N |  | V | G |  | G | G | G |
| 21/NS2B | L | L/F |  | F | L |  | L | L | L |
| 71/NS3 | S | S/N |  | N | S |  | S | S | S |
| 84/NS3 | R | R/K |  | K | R |  | R | R | R |
| 158/NS3 | N | N/S |  | N | N/S |  | N | S | N |
| 283/NS3 | M | M/V |  | V | M |  | M | M | M |
| 476/NS3 | M | T |  | T | T |  | T | T | T |
| 482/NS3 | K | N |  | N | N |  | N | N | N |
| 4/NS4A | L | L/F |  | L | L/F |  | L | F | L |
| 89/NS4A | I | V |  | V | V |  | V | V | V |
| 108/NS4B | I | T |  | T | T |  | T | T | T |
| 190/NS4B | L | F |  | F | F |  | F | F | F |
| 281/NS5 | K | R |  | R | R |  | R | R | R |
| 638/NS5 | P | L |  | L | L |  | L | L | L |
| 642/NS5 | K | K/R |  | K | K/R |  | K | R | K |
| 643/NS5 | I | I/V |  | V | I |  | I | I | I |
| 663/NS5 | T | A |  | A | A |  | A | A | A |
