# Supplementary material for: Phylogenetic relationship of dengue virus type 3 isolated in Brazil and Paraguay and global evolutionary divergence dynamics
Source: Virol J. 2012 Jun 20;9:124. doi: 10.1186/1743-422X-9-124 (PMC3494512; doi:10.1186/1743-422X-9-124)
Supplement: Additional file 4 — Motifs of amino acids for the genotype II. The file provides details on amino acid substitutions present within each genetic group of genotype II. [file 1743-422X-9-124-S4.doc]

| **Genotype II** | | | | | | | | | | |
| --- | --- | --- | --- | --- | --- | --- | --- | --- | --- | --- |
| **Position** |  | **Lineages** | | |  | **Sub-lineages (Lineage IV)** | | | | |
|  | **I** | **II** | **III** | **IV** |  | **A** | **B** | **C** | **D** | **E** |
| 35/C | R | R | R | R/K |  | R | R | R | R | K |
| 103/C | L | F | L | L |  | L | L | L | L | F |
| 108/C | M | M | I | M |  | M | M | M | M | M |
| 15/prM | G | G | G | G/A |  | A | G | G | G | G |
| 55/prM | H | F | L | L |  | L | L | L | L | L |
| 156/prM | I | V | I | I |  | I | I | I | I | I |
| 159/prM | I | M | M | M |  | M | M | M | M | M |
| 166/prM | A | A | T | T |  | T | T | T | T | T |
| 37/E | N | N | N | N/S |  | N | S | N | N | N |
| 81/E | I | I | I | I/T |  | I | T | I | I | I |
| 132/E | Y | Y | Y | Y/H |  | H | Y | Y | Y | Y |
| 124/E | S | P | P | P |  | P | P | P | P | P |
| 140/E | I | T | I | I |  | I | I | I | I | I |
| 154/E | E | D | D | D |  | D | D | D | D | D |
| 160/E | A | V | V | V |  | V | V | V | V | V |
| 172/E | I | V | I | V/I |  | I | V | V | V | V |
| 447/E | S | G | S | S |  | S | S | S | S | S |
| 479/E | A | V | A | V |  | V | V | V | V | V |
| 489/E | A | T | A | A |  | A | A | A | A | A |
| 128/NS1 | T | T | T | T/I |  | T | T | T | T | I |
| 176/NS1 | V | V | V | V/M |  | V | V | V | V | M |
| 178/NS1 | L | T | S | S |  | S | S | S | S | S |
| 188/NS1 | V | I | I | I |  | I | I | I | I | I |
| 217/NS1 | L | F | F | F |  | F | F | F | F | F |
| 338/NS1 | I | I | V | I |  | I | I | I | I | I |
| 350/NS1 | A | V | V | V |  | V | V | V | V | V |
| 34/NS2A | A | A | A | A/V |  | A | A | A | A | V |
| 38/NS2A | L | F | F | F |  | F | F | F | F | F |
| 57/NS2A | L | F | L | L |  | L | L | L | L | L |
| 133/NS2A | A | A | T | T |  | T | T | T | T | T |
| 150/NS2A | V | I | V | V |  | V | V | V | V | V |
| 180/NS2A | V | V | V | L |  | L | L | L | L | L |
| 194/NS2A | M | V | M | M |  | M | M | M | M | M |
| 195/NS2A | A | A | T | T |  | T | T | T | T | T |
| 203/NS2A | P | S | P | P |  | P | P | P | P | P |
| 214/NS2A | T | T | A | T |  | T | T | T | T | T |
| 215/NS2A | L | L | L | P |  | P | P | P | P | P |
| 282/NS3 | I | I | I | I/V |  | I | I | V | I | I |
| 324/NS3 | D | E | E | E |  | E | E | E | E | E |
| 399/NS3 | K | K | K | R/K |  | R | R | R | R/K | R |
| 589/NS3 | K | R | R | R |  | R | R | R | R | R |
| 19/NS4A | H | H | H | H/Y |  | H | H | H | Y | H |
| 100/NS4A | V | V | V | V/I |  | V | I | V | V | V |
| 115/NS4B | A | V | V | V |  | V | V | V | V | V |
| 247/NS4B | K | K | K | R/K |  | K | R | R | R | R |
| 50/NS5 | I | T | T | T |  | T | T | T | T | T |
| 52/NS5 | R | H | H | H |  | H | H | H | H | H |
| 173/NS5 | K | K | K | K/R |  | K | K | R | K | K |
| 200/NS5 | H | Y | H/Y | Y/H |  | H/Y | Y | Y | Y | Y |
| 288/NS5 | N | S | S | S |  | S | S | S | S | S |
| 338/NS5 | I | T | T | T |  | T | T | T | T | T |
| 389/NS5 | K | K | K | K/R |  | R | K | K | K | K |
| 482/NS5 | Y | Y | Y | Y/F |  | Y | F | Y | Y | Y |
| 565/NS5 | N | N | N | N/S |  | N | N | N | N | S |
| 650/NS5 | K | E | K | K |  | K | K | K | K | K |
| 679/NS5 | L | F | L/F | L |  | L | L | L | L | L |
| 785/NS5 | V | A | V | V |  | V | V | V | V | V |
| 787/NS5 | V | V | V | V/I |  | V | V | I | I | V |
| 865/NS5 | T | T | I | T |  | T | T | T | T | T |
| 876/NS5 | N | N/D | D | D |  | D | D | D | D | D |
| 890/NS5 | K | R | R | R |  | R | R | R | R | R |
