# Supplementary material for: Phylogenetic relationship of dengue virus type 3 isolated in Brazil and Paraguay and global evolutionary divergence dynamics
Source: Virol J. 2012 Jun 20;9:124. doi: 10.1186/1743-422X-9-124 (PMC3494512; doi:10.1186/1743-422X-9-124)
Supplement: Additional file 25 — Primers used for amplification and sequencing. A) Primers used in the PCR of overlapping regions representing the entire genome of DENV3. B) Primers used for nucleotide sequencing of the genome. The file provides details on all the sequences of primers used for amplification and sequencing including in this study. [file 1743-422X-9-124-S25.doc]

**Adittional file 25A.** Primers used in the PCR of overlapping regions representing the entire genome of DENV3.

|  | **Primers** | **Sequence (5’-3’)** | **Position in the viral genome** |  |
| --- | --- | --- | --- | --- |
|  | RNC5’-S/F | AGTTGTTAGTCTACGTGGACCGA | 1-23 |  |
|  | ECD3/R | CCGCACACTCCATTCTCCCAA | 2580 – 2601 |  |
|  | NS1S/F | GGGGTGTGTCATAAACTGGAAA | 2435 - 2457 |  |
|  | NS1CD3/R | ACGCCCATCCCCATTCTGTC | 3665 – 3685 |  |
|  | D3_3385S/F | GGGAGAAGACGGTTGCTGGTATGGC | 3385 – 3409 |  |
|  | D3_5880C/R | GCCAACTCTCCCTCTCCTTTGCG | 5903 – 5880 |  |
|  | D3_5730S/F | GGGACTTCGTGGTGACAACTGACAT | 5730 – 5755 |  |
|  | D3_6734C/R | GGGGGGTTCTCTGCTTTTCTGGTTC | 6734 – 6758 |  |
|  | D3_6591S/F | CAGGTAAAGGGATTGGAAAGACT | 6591 – 6614 |  |
|  | D3_8198C/R | CAGTACATTTCGTGCGTGGAGTT | 8198 – 8176 |  |
|  | D3_8150S/F | CTACAAAGGAAACATGGAGGAAT | 8150 – 8173 |  |
|  | RNC3-C/R | AGAACCTGTTGATTCAACAGCACC | 10684-10707 |  |

**Additional file 25B.** Primers used for nucleotide sequencing of the genome.

| **Primers** | **Sequence (5’-3’)** | **Position in the viral genome** |
| --- | --- | --- |
| D3_430-452/F | ACTTGCTTTCCACTTGACTTCAC | 430-452 |
| ESD3/F | CCGCACACTCCATTCTCCCAA | 845-866 |
| ESD3P1/F | GAGGAGCAGGACCAGAACTACG | 1184-1206 |
| ECD3P1/R | ACACCACCCACTGATCCAAA | 2191-2211 |
| D3_3864-3888/R | GGCTCTTAAACTGATAACACAAT | 3865 |
| D3_4772-4496/R | CTCCTCCCCCTTTTGCCATTGTGC | 4773-4797 |
| D3_4535-4558/F | C CC AGC CCC CCA GAG ACA CAG AAA | 4535-4558 |
| D3_5308/F | GGGACTTCGTGGTGACAACTGACAT | 5308-5333 |
| D3_5378-5400/R | GCTATACTGGCTGGGTCTGTGAA | 5377-5400 |
| D3_7551-7574/F | GAACAGGAAAAAGAGGAACAGGC | 7551-7574 |
| D3_6781-6804/F | CGTGATAGGCATACTTACATTGG | 6781-6804 |
| D3_7672-7696/R | CCC TTC TTT GGC TTC TGT TCT ATC C | 7672-7697 |
| D3_9518-9540/R | CCATTCTTTTTAACCTCTCCAC | 9518-9540 |
| D3_8304-8327/F | GAAGACCCACCATTGAGAAAGAT | 8304-8327 |
| D3_8889 -8914/F | CTGGGCAAGTGTGGAAGCTGTGT | 8891-8914 |
| D3_9795-9818/F | CGGGATGGAGCCTTAGAGAAACC | 9795-9818 |
| RNC3D3P1/R | GCC TGA CTT CTT CTT TTA | 10360-10378 |
