# Supplementary material for: Phylogenetic relationship of dengue virus type 3 isolated in Brazil and Paraguay and global evolutionary divergence dynamics
Source: Virol J. 2012 Jun 20;9:124. doi: 10.1186/1743-422X-9-124 (PMC3494512; doi:10.1186/1743-422X-9-124)
Supplement: Additional file 27 — Identical virus. The virus that were excluded for each data set because they are identical. [file 1743-422X-9-124-S27.doc]

Identical sequences, when analyzed the complete ORFs*.* In bold are showed the strain representative.

**TH_V2327_01** (TH_V2324_01_TH_V2325_01); **TH_V2312_01** (TH_V2314_01); **98TW360** (98TW349); **98TW358** (98TW364); **IPC_V3831_07** (IPC_V3808_08); **SRI_00_1266** (NC_001475.2); **SG_05K4647DK1_05** (SG_05K3312DK1_05); **BR_V2983_03** (BR_V2388_03); **BR_V3451_06** (BR_V3429_06); **BR_V3464_06** (BR_V3427_06); **BR_V3588_07** (BR_V3470_07); **PR_V1091_04** (PR_V1090_98_PR_V1611_04); **CO_V3402_05** (CO_V2986_05); **CO_V3399_03** (CO_V2984_03); **CO_V2978_01** (CO_V3392_01); **VE_V1149_07** (VE_V2483_07_VE_V1150_07); **VE_V2268_08** (VE_V2267_08) **VE_V907_01** (VE_V906_01); **PR_V1604_04** (PR_V1079_06); **PR_V1050_98** (PR_V1448_98)

Identical sequences, when analyzed the C protein*.* In bold are showed the strain representative.

**KH_V2079_02** (KH_V2078_02); **KH_V2080_03** (KH_V2077_02); **H_IMTSSA_SRI_00_1266** (NC_001475.2); **SG_05K4477DK1_05** (SG_05K4440DK1_05); **IN_98901517DHFDV_3_98** (IN_98901437DSSDV_3_98); **IN_TB55i_04** (IN_KJ30i_04); **CHINA_80_2** (BRDEN3RO1_02_ M93130); **Th0104_93** (TH_C0331_94_Th0055_93); **D3MY00_22366** (Th1283_98); **D3MY02_25811** (D3MY02_25850); **TH_CH53489_1973** (TH_V3360_1973); **D3MY95_3952** (D3MY95_2471_D3MY96_4269); **CO_V3405_07** (VE_V915_01_ CO_V3403_05); **BR_V3457_06** (BR_V3463_06); **D3BR_RP1_03** (BR_V3591_07_BR_V3609_07); **BR_V3417_06** (BR_V3601_07_BR_V3456_06); **LK_V2410_83** (LK_V2407_83_LK_V2412_89); **LK_V2405_83** (LK_V2414_1985); **VE_V2481_07** (VE_V2266_06); **CO_V3402_05** (CO_V2986_05); **TT_V3928_02** (TT_V2982_02); **VE_V2242_05** (VE_V2222_04); **VE_V1114_01** (VE_V2185_01); **NI_V3073_08** (NI_V2649_08**); PR_V1451_99** (VE_V2195_01_VE_V2199_01); **VE_V2213_03** (VE_V2196_01_VE_V2207_01); VE_V1116_01 (VE_V913_01); **PR_V1614_04** (PR_V1475_02); **VE_V2267_08** (VE_V1102_07_VE_V2268_08); **IPC_V4306_07** (IPC_V4302_07); **VN_V1817_07** (VN_V1815_07); **VN_V1817_07** (VN_V1815_07); **PR_V1730_03** (PR_V1608_04); **TH_V2329_01** (TH_V2328_01_TH_V2312_01_TH_V2318_01_TH_V2327_01_ TH_V2326_01_TH_V2325_01_TH_V2324_01_ TH_V2320_01_TH_V2317_01_TH_V2316_01_TH_V2315_01_TH_V2314_01_TH_V2313_01_TH_V2321_01_TH_V2322_01_ TH_V2319_01_TH_V2323_01); **KH_V2082_03** (IPC_V3824_06_IPC_V4280_06_IPC_V4283_07_IPC_V3825_06_ _IPC_V3822_06_IPC_V3820_06_ IPC_V3809_03_IPC_V3819_06_IPC_V3830_07_IPC_V3804_08);

**KH_V2088_05** (KH_V2089_06_NI_V3073_08_NI_V2649_08_KH_V2085_05_ IPC_V4286_07_IPC_V3831_07_IPC_V3808_08_KH_V2087_05); **KH_V2081_03** (IPC_V3815_06_IPC_V4308_07_IPC_V4298_07_PC_V4284_07_IPC_V4282_07_); **BRDEN3290_02** (PR_V2108_00_PR_V1478_02_PR_V1490_03_PR_V1089_03_PR_V2100_00_PR_V2115_01_PR_V1465_00); **SG_05K4648DK1_05** (SG_05K4647DK1_05_SG_05K4144DK1_05_SG_05K3312DK1_05); **SG_05K863DK1_05** (SG_05K827DK1_05_SG_05K802DK1_05); **SG_05K4182DK1_05** (SG_05K4176DK1_05_SG_05K4168DK1_05_ SG_05K4159DK1_05_SG_05K4141DK1_05_SG_05K3923DK1_05_SG_05K3912DK1_05_SG_05K3900DK1_05_SG_05K3329DK1_05_SG_05K3325DK1_05_SG_05K3324DK1_05_SG_05K3305DK1_05_SG_05K2918DK1_05_SG_05K2899DK1_05_SG_05K2418DK1_05_SG_05K2406DK1_05_SG_05K871DK1_05_SG_05K868DK1_05_ SG_05K852DK1_05_SG_05K845DK1_05_ SG_05K805DK1_05_SG_05K797DK1_05_SG_05K791DK1_05_SG_05K3927DK1_05_SG_05K4157DK1_05_SG_05K3887DK1_05_SG_05K2933DK1_05_SG_05K2400DK1_05_SG_05K843DK1_05); **Hu_TL129NIID_05** (Hu_TL109NIID_05_Hu_TL029NIID_05_Hu_TL018NIID_05)

**D3MY05_34640** (D3MY05_33927_D3MY05_33610_IN_TB16_04_IN_den3_98_04_D3MY03_27834_D3MY00_22583_D3MY00_22460_D3MY00_22447_IN_PI64_04_ D3MY00_22550); **PF89_27643_89** (CK_V2972_91_ PF92_4190_92_ PF92_2986_92_PF92_2956_92_ PF90_3056_90_D3MY00_22460_D3MY00_22447_IN_PI64_04_ PF90_6056_90); **98TW503** (98TWmosq_98TW434_98TW414_98TW407_98TW390_98TW388_98TW368_98TW36498TW360_98TW358_98TW349); **BDH02_7_02** (BDH02_4_02_BDH02_3_02_D3MY04_33077_BDH02_1_02); **BR_V3444_06** (BR_V3606_07_BR_V3465_06_BR_V3446_06_ BR_V3442_06_BR_V3423_06);

**BR_V3605_07** (BR_V3593_07_ BR_V3590_07_BR_V3588_07_ BR_V3469_07_BR_V3464_06_BR_V3460_06_BR_V3451_06

BR_V3441_06_BR_V3434_06_BR_V3430_06_BR_V3429_06_ BR_V3427_06_BR_V3424_06_BR_V3431_06_BR_V3435_06

BR_V3584_06_BR_V3470_07); BR_V3598_07 (PR_V1732_02_PR_V2122_02_PR_V2120_02); **VE_V2965_00** (VE_V2453_01_ VE_V2178_00_ VE_V2204_01_ VE_V2203_01_VE_V2188_01_VE_V2175_00_VE_V2174_00

VE_V2190_01); **NI_V3072_08** (NI_V3068_08_NI_V3066_08_NI_V3057_08_ NI_V2644_08_ NI_V2934_08_ NI_V2647_08_ NI_V2654_08_NI_V3055_08); **AI_V2976_01** (LC_V2979_01_CO_V3398_03_LC_V3929_01_ BR_V2983_03_CO_V2988_07_ BR_V2400_07_ BR_V2397_06_BR_V2391_04_BR_V2388_03); **CO_V3395_03** (BRDEN397_04_BRDEN395_04_ BR74886_02__D3MY01_24056_99TW628_BR73354mosq_01_VE_V1149_07_PR_V1736_99_CO_V3400_04_CO_V3395_03_CO_V3393_02_VE_V2483_07_CO_V2978_01_CO_V3399_03_CO_V3392_01_VE_V905_01_PR_V859_98_BR_V2380_01_VE_V2970_03_VE_V2969_03_VE_V2967_01_VE_V2966_01_BR_V2977_01_CO_V2984_03_VE_V2202_01_VE_V2200_01_NI_V2420_1994_VE_V2484_07_VE_V2480_07_ BR_V2387_03_VE_V1113_01); **PR_V1090_98** (EC_V2975_00_ VE_V2971_07_ **PE_V2981_02** (PR_V1613_04_PR_V1612_04_PR_V1091_04__PR_V1611_04_VE_V1593_05); **VE_V2184_01** (VE_V2183_01_VE_V2181_01_VE_V2180_01_VE_V2179_00_VE_V2182_01); **PR_V1616_04** (PR_V1623_05_PR_V1610_04_PR_V1609_04_ PR_V1044_06_PR_V1078_03_PR_V1043_06);

**PR_V1417_07** (PR_V1416_07_PR_V1415_07_PR_V1077_00); **PR_V1729_03** (PR_V2118_01_ PR_V1606_04_PR_V1731_03_ PR_V1624_05_PR_V1622_05_PR_V1620_05_PR_V1619_05_PR_V1617_05_PR_V1604_04_PR_V1491_03_PR_V1079_06_ PR_V1075_98_PR_V1605_04__PR_V2123_02_PR_V1621_05_ PR_V1607_04_PR_V1473_02_PR_V1615_04_PR_V1625_05);

**PR_V1460_00** (PR_V1049_98_VE_V2208_02_VE_V2209_02_ VE_V2212_03_VE_V2214_03_VE_V2218_03_VE_V2220_04

PR_V1050_98_VE_V2193_01_VE_V2189_01_VE_V2205_07_ VE_V2198_01_PR_V1088_98_VE_V906_01_VE_V1117_01

VE_V1118_01_VE_V903_01_VE_V907_01_VE_V2234_04_ VE_V2257_06_VE_V908_01_VE_V1115_01_VE_V912_01

PR_V2098_99_VE_V2244_05_PR_V1452_99_VE_V2239_05_ VE_V1591_04_PR_V2099_98_PR_V2111_00_PR_V1448_98

PR_V1450_98_PR_V2126_06_VE_V2240_05_VE_V1585_01_ VE_V1150_07_VE_V2186_01_PR_V2105_00_PR_V1454_99

PR_V2104_00_PR_V1466_99_VE_V2228_04_VE_V2224_04_ VE_V2197_01_PR_V1737_99_PR_V1735_99_PR_V1733_99

VE_V916_01_VE_V2192_01_VE_V2232_04_PR_V1455_99_VE_V2211_02); **PR_V1477_02** (PR_V2114_01_ PR_V2113_00_PR_V2112_00_VE_V2215_03_PR_V1480_03); **VN_V1911_08** (VN_V1327_06_VN_V1017_07_ VN_V1015_06_VN_V1810_07_VN_V1831_07_DENV_3VN_V1936_08_VN_V1891_07_VN_V1877_07_VN_V1783_07_VN_V1882_07_ VN_V1946_08_VN_V1897_07_VN_V1824_07_VN_V1833_07); **VN_V1326_06** (VN_V1330_ 06_VN_V1018_07_ VN_V1014_06_VN_V1011_06_VN_V1957_08_VN_V1769_07_VN_V1790_07); **IPC_V4294_07** (IPC_V3833_07_IPC_V3832_07_IPC_V3828_07_IPC_V3827_07_DTID_ZJU04_KH_V2054_08_KH_V2086_050); **CH_CHLS001_07** (KH_V2083_04_KH_V2053_08_KH_V2051_07).

Identical sequences, when analyzed the prM protein*.* In bold are showed the strain representative.

**IPC_V3828_07** (IPC_V3827_07); **KH_V2082_03** (IPC_V3809_03); **IPC_V4306_07** (IPC_V4302_07); **KH_V2085_05** (IPC_V4280_06_IPC_V3807_05); **KH_V2075_01** (KH_V2077_02); **TH_V2321_01** (TH_V2319_01); BR_PV1_03 (BRDEN397_04); **H_IMTSSA_SRI_00_1266** (NC_001475.2); **Singapore** (99TW62); **SG_05K4477_05** (SG_05K4440_05); **MY_32645_04** (MY_34640_05_ MY_33610_05); MY**_22583_00** (MY_22550_00_ MY_22460_00); IN**_98901517_9**8 (IN_98901437_98); **IN_TB55i_04** (IN_KJ30i_04); **Hu_TL029NIID_05** (Hu_TL018NIID_05); **CH_80_2** (DTID_ZJU04); **Th0010_87** (Th0007_87 ); **BDH02_7_02** (BDH02_4_02_BDH02_3_02); **MY_25850_02** (MY02_25811); **VE_V907_01** (VE_V906_01); **VE_V2205_07** (VE_V2189_01); **VE_V2452_01** (VE_V1114_01_VE_V2199_01); **VE_V2244_05** (VE_V2234_04); **VE_V2970_03** (VE_V2209_02_VE_V2208_02); **BR_V2380_01** (BR_V2977_01); **VE_V2267_08** (PR_V2126_06_VE_V2268_08); **PR_V1466_99** (PR_V2111_00); **VE_V2481_07** (VE_V2266_06); **PR_V1616_04** (PR_V1610_04); **PR_V1604_04** (PR_V1079_06); **PR_V1731_03** (PR_V2123_02_PR_V1621_05); **VE_V2200_01** (VE_V2192_01); **VE_V2232_04** (VE_V2207_01); **BR_V3417_06** (BR_V3601_07_BR_V3456_06); **BR_V3584_06** (BR_V3593_07_BR_V3469_07); **BR_V2983_03** (BR_V2394_05_BR_V2388_03); **BR_V2403_08** (BR_V2400_07_BR_V2397_06); **LC_V3929_01** (LC_V2979_01); **TT_V3928_02** (TT_V2982_02); **LK_V2405_1983** (LK_V2414_1985_LK_V2407_1983); **CK_V2972_1991**_ **PF90_3050_90** (PF89_320219_89_ PF92_2956_92_PF_27643_89 PF92_4190_92_ PF90_3056_90); **VN_V1810_07** (VN_V1877_07); **KH_V2054_08** (KH_V2053_08); KH_V2052_07(IPC_V4308_07_IPC_V4298_07_IPC_V4284_07_IPC_V3815_06_IPC_V4290_07);**VN_V1817_07** (VN_V1815_07_VN_V1326_06);**PR_V2105_00**(PR_V1732_02_PR_V1460_00_PR_V2122_02_PR_V2120_02_PR_V1476_02);**VE_V1149_07**(VE_V2483_07_VE_V2484_07_VE_V1150_07);**IPC_V3808_08** (IPC_V3824_06_KH_V2086_ 05_KH_V2089_06_KH_V2088_05_IPC_V4286_07_IPC_V3831_07_IPC_V4297_07_IPC_V3825_06_ IPC_V3820_06_IPC_V3819_06_IPC_V3826_07); **VN_V1783_07** (VN_V1946_08_VN_V1897_07_VN_V1824_07_ VN_V1017_07_VN_V1327_06_VN_V1015_06); **VN_V1874_08** (VN_V1831_07_VN_V1936_08_VN_V1018_07_ VN_V1330_06); **VE_V2256_05** (VE_V2226_04_VE_V2217_03_VE_V1113_01); **PR_V859_98** (PR_V2114_01_PR_V1447_98_PR_V1451_99_PR_V1449_98_PR_V1448_98_PR_V1050_98_PR_V1049_98_PR_V2099_98); **NI_V3073_08**(NI_V2649_08_NI_V3072_08_NI_V3066_08_NI_V3059_08_NI_V3057_08_NI_V2644_08_NI_V2934_08_NI_V2935_08_NI_V2647_08_NI_V2654_08_NI_V2653_08);**BR_V3424_06**(BR_V3606_07_BR_V3590_07_BR_V3588_07_BR_V3470_07_BR_V3465_06_BR_V3464_06_BR_V3460_06_BR_V3451_06_BR_V3446_06_BR_V3442_06_BR_V3441_06_BR_V3434_06_BR_V3429_06_BR_V3427_06_BR_V3423_06_BR_V3444_06_BR_V3431_06_BR_V3457_06_BR_V3463_06); **PR_V1609_04** (PR_V1044_06_PR_V1078_03_PR_V1043_06_PR_V1076_99); **PR_V1090_98** (PR_V1612_04_PR_V1091_04_PR_V1611_04);PR_V1622_05(PR_V1620_05_PR_V1075_98_PR_V1605_04_PR_V1619_05_PR_V1417_07_PR_V1416_07_PR_V1415_07_PR_V1077_00_PR_V1625_05);**PR_V2118_01**(PR_V1481_03_PR_V1473_02_PR_V1624_05_PR_V1491_03);PR_V1465_00_PR_V1475_02_PR_V2100_00_PR_V2115_01_PR_V2108_00_PR_V2107_00_PR_V1092_04_PR_V2110_00_PR_V1614_04_PR_V1453_99); **PR_V1454_99** (PR_V1735_99_PR_V1733_99); **PR_V2098_99** (VE_V905_01_VE_V2202_01_BR_V2387_03_VE_V2965_00_VE_V2455_01_VE_V2453_01_VE_V908_01_ VE_V1117_01_VE_V912_01_VE_V915_01_VE_V2201_01_VE_V2181_01_VE_V2180_01_VE_V2184_01_ VE_V2183_01_VE_V2185_01_PR_V2098_99_VE_V2198_01_VE_V2179_00_VE_V2186_01_VE_V2210_02_VE_V2197_01_ VE_V2182_01_VE_V1116_01_VE_V913_01_VE_V2188_01_VE_V2175_00_VE_V2174_00_VE_V2204_01_VE_V2178_00_VE_V904_01_VE_V2190_01_VE_V2231_04);**TH_V2329_01**(TH_V2312_01_TH_V2318_01_TH_V2327_01_TH_V2326_01_TH_V2325_01_TH_V2324_01_TH_V2323_01_TH_V2320_01_ TH_V2317_01_TH_V2315_01_TH_V2314_01_TH_V2313_01); **SG_05K4159_05** (SG_05K3900_05_SG_05K3887_05_SG_05K3329_05); SG_SS710_04 (SG_05K4648_05_SG_05K4647_05_SG_05K4454_05_SG_05K4182_05_SG_05K4176_05_SG_05K4168_05_SG_05K4157_05_ SG_05K4144_05_SG_05K4141_05_SG_05K3928_05_SG_05K3927_05_SG_05K3923_05_SG_05K3912_05_SG_05K3897_05_ SG_05K3325_05_SG_05K3324_05_SG_05K3312_05_SG_05K3305_05_SG_05K2933_05_SG_05K2918_05_SG_05K2400_05_ SG_05K871_05_SG_05K868_05_SG_05K852_05_SG_05K845_05_SG_05K843_05_SG_05K827_05_SG_05K805_05_ SG_05K802_05_SG_05K797_05_SG_05K791_05_SG_05K3316_05_SG_05K2899_05_SG_05K2418_05_SG_05K3314_05); **98TW503** (98TWmosq_98TW414_98TW407_98TW390_98TW368_98TW364_98TW360_98TW358_98TW349); **CO_V3402_05** (CO_V2986_05_VE_V2967_01_VE_V2966_01_VE_V2454_01); **CO_V3393_02** (CO_V2978_01_CO_V3401_04_CO_V3399_03_CO_V3392_01_CO_V2984_03_CO_V3397_03_VE_V2193_01); PY_AS12_02_ **BR_RP1_03** (BRDEN395_04_BR74886_02_ BR73354mosq_01)

Identical sequences, when analyzed the E protein*.* In bold are showed the strain representative.

**MY00_22583** (MY00_22550_ MY00_22460); **TH_V2312_01**(TH_V2314_01_ TH_V2313_01); **IPC_V4298_07** (IPC_V4290_07); **KH_V2054_08** (KH_V2053_08); **IPC_V3828_07**(IPC_V3808_08); **IPC_V3809_03** (KH_V2082_03); **IPC_V4306_07** (IPC_V4302_07); **VN_V1817_07** (VN_V1815_07); **VN_V1330_06** (VN_V1018_07); **BR_V3465_06** (BR_V3446_06_BR_V3444_06); **BR_V3451_06**(BR_V3429_06); **BR_V3588_07**(BR_V3470_07_BR_V3434_06); **BR_V3441_06** (BR_V3424_06_BR_V3431_06); **BR_V3601_07** (BR_V3456_06_BR_V3417_06); **BR_V2380_01** (BR_V2977_01); **PR_V1618_05** (PR_V1609_04); PR_V1044_06(PR_V1078_03); **PR_V1616_04**(PR_V1610_04); **BR_V2983_03**(BR_V2388_03); **TT_V3928_02** (TT_V2982_02); **MART_99_1243** (NC_001475.2); **SG_05K4647_05** (SG_05K4144_05_SG_05K3312_05); **SG_05K3897_05** (SG_05K3316_05); SG**_05K4159_05** (SG_05K3900_05_SG_05K3329_05); **SG_05K4141_05** (SG_05K3912_05); **LK_V2405_83**(LK_V2414_85); **CO_V3399_03**(CO_V2984_03); **VE_V1591_04** (VE_V2218_03_VE_V2228_04); **VE_V2454_01** (VE_V2966_01); **CO_V3402_05** (CO_V2986_05); **VE_V2267_08** (VE_V1102_07_VE_V2268_08); **VE_V907_01** (VE_V906_01); **VE_V2195_01** (VE_V1113_01); **VE_V2199_01** (VE_V2452_01**); CO_V2978_01** (CO_V3392_01); **VE_V912_01** (VE_V905_01); **PR_V1733_99** (PR_V1454_99); **PR_V2110_00** (PR_V2108_00_PR_V1453_99); **PR_V1465_00** (PR_V2106_00); **PR_V1415_07**(PR_V1077_00); **NI_V2649_08** (NI_V3069_2009_NI_V2654_08); **NI_V2935_08** (NI_V2647_08); **NI_V3072_08** (NI_V2644_08_NI_V2934_08); **PR_V1091_04** (PR_V1090_98_PR_V1611_04); **TH_V2318_01** (TH_V2327_01_TH_V2325_01_TH_V2324_01_TH_V2323_01_TH_V2321_01_TH_V2320_01_TH_V2317_01_ TH_V2319_01_TH_V2316_01_TH_V2322_01); **98TW434** (98TW390_98TW388_98TW503_ 98TW364_98TW360_98TW358_98TW349);**IPC_V3825_06** (IPC_V3822_06_IPC_V3820_06_IPC_V3819_06_ IPC_V3824_06); **VN_V1327_06** (VN_V1017_07_VN_V1783_07_VN_V1897_07); **BR_V3584_06** (BR_V3464_06_BR_V3457_06_ BR_V3442_06_BR_V3427_06); **SG_05K3324_05** (SG_05K827_05_SG_05K805_05_ SG_05K791_05_ SG_05K863_05); **SG_05K4454_05** (SG_05K4182_05_ SG_05K4176_05_ SG_05K4168_05_SG_05K3928_05_ SG_05K3314_05_ SG_05K2400_05_ SG_05K871_05_ SG_05K868_05_SG_05K852_05_SG_05K845_05_SG_05K843_05_SG_05K797_05_SG_SS710_04_ SG_05K4157_05_SG_05K3305_05); **CO_V3400_04** (VE_V2197_01_VE_V2175_00_VE_V2965_00_ VE_V2174_00_ VE_V2178_00); **VE_V2184_01** (VE_V2183_01_ VE_V2181_01_VE_V2180_01_VE_V2182_01); **VE_V2483_07** (VE_V2484_07_VE_V1149_07_ VE_V2480_07_ VE_V1150_07); **PR_V1050_98** (PR_V1448_98_ PR_V1447_98_PR_V1451_99_PR_V1088_98_ PR_V1737_99_PR_V1455_99_PR_V1736_99); **PR_V1473_02** (PR_V1731_03_PR_V2118_01_PR_V2123_02)

Identical sequences, when analyzed the NS1 protein*.* In bold are showed the strain representative.

**KH_V2077_02** (KH_V2075_01); **KH_V2079_02** (KH_V2078_02)_ **MY_25850_02** (MY_25811_02_MY_22366_00); **KH_V2087_05** (KH_V2082_03); **KH_V2054_08** (KH_V2053_08); **VN_V1817_07** (VN_V1815_07); **VN_V1783_07** (VN_V1897_07_VN_V1946_08); **BD_7_02** (BD_3_02_BD_4_02); **SG_05K3325_05** (SG_05K3928_05); **TT_V3928_02** (TT_V2982_02); **LC_V3929_01** (LC_V2979_01); **PR_V1044_06** (PR_V1076_99_PR_V1043_06); **PR_V1609_04** (PR_V1616_04_PR_V1610_04); **BR_V3456_06** (BR_V3417_06); **BR_V3588_07** (BR_V3434_06); **NI_V3057_08** (NI_V2654_08_NI_V3069_09); **NI_V3072_08** (NI_V2934_08_NI_V3055_08); **PR_V1091_04** (PR_V1613_04_PR_V1612_04); **VE_V905_01** (VE_V912_01); **VE_V1149_07** (VE_V2480_07_VE_V2484_07); **VE_V2268_08** (VE_V1102_07); **VE_V2213_03** (VE_V2207_01); **VE_V2257_06** (VE_V2232_04); VE_V2234_04 **(VE_V2220_04)**; VE_V2189_01 (VE_V1115_01); **PR_V2120_02** (PR_V1732_02); **PR_V1460_00** (PR_V1454_99_PR_V1735_99); **PR_V1625_05** (PR_V1620_05_PR_V1075_98); **PR_V1416_07** (PR_V1077_00); **PR_V1605_04** (PR_V1604_04); **PR_V1466_99** (PR_V2111_00); **LK_V2405_83** (LK_V2414_85); **TH_V2327_01** (TH_V2320_01_TH_V2318_01_TH_V2319_01_TH_V2326_01_TH_V2317_01_ TH_V2321_01_TH_V2323_01_TH_V2313_01_TH_V2312_01_TH_V2315_01_TH_V2316_01); **TW_mosq_98** (TW_414_98_TW_434_98_TW_390_98_TW_358_98_TW_360_98_ TW_368_98_TW_503_98_TW_388_98_TW_407_98); **SG_05K4647_05** (SG_05K4144_05_SG_05K4648_05_SG_05K3324_05_SG_05K791_05_SG_05K802_05_ SG_05K2899_05_SG_05K827_05_SG_05K863_05_SG_05K2418_05); **SG_05K871_05** (SG_05K3897_05_SG_05K3316_05_SG_05K4168_05_SG_05K4176_05_SG_05K3927_05_ SG_05K3913_05_SG_05K3887_05_SG_05K3329_05_SG_05K4159_05_SG_05K3900_05); **SG_05K843_05** (SG_05K3305_05_SG_05K868_05_SG_05K2933_05_SG_05K845_05_SG_05K4182_05); **VN_V1882_07** (VN_V1877_07_VN_V1831_07_VN_V1891_07_VN_V1911_08); **KH_V4308_07** (KH_V4298_07_KH_V4290_07_KH_V4284_07); **VN_V1786_07** (VN_V1824_07_VN_V1017_07_VN_V1015_06); **BR_V3442_06** (BR_V3423_06_BR_V3590_07_BR_V3424_06_BR_V3457_06_BR_V3464_06_BR_V3463_06_BR_V3431_06_BR_V3584_06_BR_V3469_07_BR_V3430_06_ BR_V3435_06); **VE_V2454_01** (CO_V3394_03_CO_V3393_02_VE_V2966_01_VE_V2191_01_VE_V2967_01_VE_V2452_01_VE_V2199_01_VE_V2195_01_VE_V907_01_VE_V2205_07_VE_V2192_01_VE_V1114_01_VE_V2185_01_ VE_V1118_01_VE_V2198_01_VE_V2190_01_VE_V2178_00_VE_V2174_00); **VE_V2186_01** (VE_V2184_01_VE_V2183_01_VE_V2180_01_VE_V2182_01_VE_V2181_01_VE_V2179_00); **PR_V1729_03** (PR_V1491_03_PR_V1481_03_PR_V1626_05); **PR_V2100_00** (PR_V2110_00_PR_V1465_00_PR_V2106_00); **PR_V2098_99** (PR_V2099_98_PR_V1450_98_PR_V1050_98_PR_V1049_98_PR_V1449_98_PR_V2112_00_PR_V859_98).

Identical sequences, when analyzed the NS2A protein*.* In bold are showed the strain representative.

**SG_05K4477_05** (SG_05K4440_05); **MY_34640_05** (MY_33610_05_MY_33927_05_MY_33506_05); **IN_TB55i_04** (IN_KJ30i_04_IN_TB16_04); **MY_22460_00** (MY_22550_00); **CK_V2972_91(**PF_320219_89 _PF_3056_90_PF_27643_89_PF_6056_90); **TH_0104_93** (KH_V2077_02_KH_V2080_03_KH_V2075_01); **MY_25850_02** (MY_25811_02); **KH_V2085_05** (KH_V2088_05); **KH_V4306_07** (KH_V4302_07); **KH_V4280_06** (KH_V3807_05); **KH_V4298_07** (KH_V4290_07); **KH_V4308_07** (KH_V4284_07_KH_V4282_07); **CH_CHLS001_07(**KH_V3833_07 _KH_V2051_07); **KH_V3828_07** (KH_V3827_07); **KH_V2054_08** (KH_V2053_08); **VN_V1817_07** (VN_V1815_07); **VN_V1882_07** (VN_V1831_07_VN_V1810_07_VN_V1911_08_VN_V1017_07); **VN_V1783_07** (VN_V1946_08_VN_V1824_07_VN_V1015_06); **VN_V1011_06** (VN_V1953_08); **BD_3_02** (BD_4_02); **SG_05K3912_05** (SG_05K4141_05); **TT_V3928_02** (TT_V2982_02); **LC_V3929_01** (LC_V2979_01); **PR_V1044_06** (PR_V1078_03_PR_V1618_05_PR_V1609_04_PR_V1610_04); **MART_1243_99** (BR_V2391_04); **BR_V3615_07** (BR_V3456_06_BR_V3417_06_BR_V3601_07); **BR_V3606_07** (BR_V3444_06); **BR_V3465_06**(BR_V3446_06_BR_V3442_06_BR_V3423_06); **BR_V3588_07** (BR_V3460_06_BR_V3434_06); **BR_V3584_06** (BR_V3593_07_BR_V3605_07); **NI_V2644_08**(NI_V2934_08_NI_V3055_08); **NI_V2935_08** (NI_V2647_08); **NI_V3066_08** (NI_V2649_08_NI_V3073_08_NI_V3059_08_NI_V2653_08); **PR_V1091_04** (PR_V1613_04_VE_V2971_07); **VE_V905_01** (VE_V912_01); **CO_V3395_03** (CO_V2978_01); **VE_V2268_08 (**VE_V1102_07); **VE_V2452_01** (VE_V2199_01_VE_V2195_01); **VE_V2200_01** (VE_V2192_01); **VE_V2244_05** (VE_V2234_04_VE_V2220_04); **VE_V2222_04** (VE_V2223_04_VE_V2969_03); **VE_V2207_01** (VE_V2204_01); **VE_V2188_01** (VE_V2203_01); **VE_V2970_03** (VE_V2208_02_VE_V2209_02); **PR_V2105_00** (PR_V1460_00_PR_V1733_99_PR_V1735_99); **PR_V1729_03**(PR_V1615_04); **PR_V1731_03** (PR_V2123_02), **PR_V1624_05** (PR_V1491_03); **PR_V1473_02** (PR_V2118_01); **PR_V1614_04** (PR_V1092_04_PR_V1475_02); **PR_V1050_98** (PR_V1049_98); **PR_V1466_99**(PR_V2111_00); **LK_V2410_83** (LK_V2412_89); **LK_V2405_83** (LK_V2414_85_LK_V2407_83); **TH_V2327_01** (TH_V2320_01_TH_V2318_01_TH_V2319_01_TH_V2326_01­_ TH_V2321_01_TH_V2322_01_TH_V2323_01_TH_V2313_01_TH_V2312_01_ TH_V2317_01, TH_V2316_01, TH_V2315_01); **TW_mosq_98_TW** (414_98_TW_434_98_ TW_390_98_ TW_358_98_ TW_360_98_ TW_368_98_TW_503_98_ TW_388_98_TW_407_98); **KH_V3825_06** (KH_V3822_06_KH_V3820_06_KH_V3819_06_KH_V4283_07_ KH_V2090_06); **SG_05K3887_05** (SG_05K3329_05_SG_05K4159_05_SG_05K4157_05_SG_05K3900_05_SG_05K3314_05_SG_05K797_05_SG_05K852_05_SG_05K2406_05_SG_05K2400_05); **SG_05K4647_05** (SG_05K4144_05_SG_05K4648_05_SG_05K3324_05_SG_05K791_05_SG_05K802_05_SG_05K827_05_SG_05K863_05_SG_05K805_05_SG_05K3923_05_SG_05K871_05_SG_05K843_05_SG_05K3897_05_SG_05K3316_05_SG_05K4168_05_SG_05K4176_05_SG_05K3927_05_SG_05K3913_05_SG_05K3928_05_SG _05K3305_05_SG_05K868_05_SG_05K2933_05_SG_05K845_05_ SG_05K4182_05_ SG_SS710_04); **BR_V3424_06** (BR_V3441_06_BR_V3457_06_BR_V3451_06_BR_V3464_06_ BR_V3435_06_BR_V3463_06_BR_V3431_06); **VE_V2454_01** (VE_V2966_01_VE_V2191_01_VE_V907_01_VE_V2215_03_VE_V1113_01_VE_V1115_01_VE_V1114_01_VE_V2185_01_VE_V2219_03_VE_V904_01_VE_V2201_01_VE_V2198_01_VE_V1116_01_VE_V913_01_VE_V2455_01_VE_V2965_00_VE_V2178_00_VE_V2174_00_VE_V2175_00_VE_V2186_01_VE_V2184_01_VE_V2183_01_VE_V2180_01_VE_V2181_01_VE_V2179_00_VE_V1585_01_VE_V2193_01, VE_V2240_05); **PR_V1625_05** (PR_V1620_05_PR_V1622_05_PR_V1417_07_PR_V1415_07_PR_V1619_05_PR_V1605_04_PR_V1604_04); **PR_V2098_99** (PR_V2099_98_PR_V1455_99_PR_V2100_00_PR_V2106_00_ PR_V1453_99_PR_V1452_99_PR_V1737_99_PR_V1088_98_PR_V1447_98_PR_V1080_06_PR_V859_98).

Identical sequences, when analyzed the NS2B protein*.* In bold are showed the strain representative.

**SG_05K4477_05** (SG_05K4440_05); **IN_TB55i_04** (IN_KJ30i_04); **MY_22583_00** (MY_22460_00_MY_22550_00); **IN_PH86_04** (IN_KJ71_04); **ET_TL029_05** (ET_TL018_05_ET_TL129_05_ET_TL109_05); **PF_4190_92** (PF_2986_92_PF_3056_90_ PF_27643_89_PF_3050_90); **TH_V2328_01** (TH_V2329_01); **KH_V2078_02 (**KH_V2071_00); **MY_25850_02** (MY_25811_02) ;KH_V3830_07(KH_V2050_07; **KH_V4280_06** (KH_V3807_05); **KH_V4308_07** (KH_V4298_07_KH_V4290_07_KH_V4284_07); **KH_V4282_07** (KH_V2052_07); **KH_V2054_08** (KH_V2053_08); **VN_V1817_07** (VN_V1815_07_VN_V1790_07); **VN_V1330_06** (VN_V1018_07); **VN_V1874_08** (VN_V1957_08_ VN_V1014_06_VN_V1011_06**); BD_7_02**(BD_3_02_BD_1_02); **TH_V3360_73** (TH_CH53489_73**); BR_V2983_03** (BR_V2394_05_BR_V2397_06_ BR_V2391_04); BR_V3456_06_BR_V3417_06_BR_V3601_07); **NI_V2935_08** (NI_V2647_08); **EC_V2975_00** (PR_V1091_04_PR_V1612_04); **CO_V3397_03** (CO_V3395_03_CO_V3399_03_ CO_V2978_01_CO_V3398_03_ CO_V3393_02); **VE_V2217_03** (VE_V2215_03); **VE_V2200_01** (VE_V2192_01); **VE_V2224_04** (VE_V2233_04); **CO_V3402_05** (VE_V2178_00_VE_V2174_00_VE_V2175_00);**VE_V2970_03** (VE_V2208_02_ VE_V2209_02); **PR_V1614_04 (**PR_V1092_04_PR_V1475_02);(PR_V1490_03)**PR_V2108_00**; (PR_V1736_99_ PR_V1452_99_PR_V1737_99); **PR_V2113_00** (MX_V2989_07); **LK_V2410_83** (LK_V2412_89); **LK_V2405_83** (LK_V2414_85); **MY_34640_05** (MY_33610_05_MY_33927_05_MY_33506_05_MY_28526_00_ MY_22447_00_ IN_98901517_98 _IN_98901437_98_IN_den98_04); **TH_V2327_01** (TH_V2320_01_TH_V2318_01_TH_V2319_01_ TH_V2326_01_TH_V2317_01_ TH_V2321_01_ TH_V2322_01_TH_V2323_01_TH_V2313_01); **TH_V2312_01** (_TH_V2315_01_TH_V2316_01); **TW_mosq_98** (TW_414_98_TW_434_98_TW_390_98_TW_358_98_TW_360_98_TW_368_98_TW_503_98_TW_388_98); **KH_V4306_07** (KH_V4302_07_KH_V3825_06_KH_V3822_06_KH_V3820_06_KH_V4297_07_KH_V3819_06_KH_V3824_06); **KH_V4314_08** (KH_V4286_07_KH_V2087_05_KH_V2089_06_KH_V2085_05_KH_V2088_05_KH_V3809_03_KH_V2082_03_KH_V3832_07_CH_CHLS001_07); **KH_V2051_07** (KH_V3826_07_KH_V3828_07_KH_V3827_07_VN_V1326_06_VN_V1008_06); **VN_V1882_07** (VN_V1877_07_VN_V1810_07_VN_V1891_07_VN_V1911_08_VN_V1936_08_VN_V1783_07_VN_V1897_07_VN_V1946_08_VN_V1824_07_VN_V1833_07); **VN_V1327_06** (VN_V1017_07_VN_V1015_06); **SG_05K3887_05** (SG_05K3329_05_SG_05K4159_05_SG_05K4157_05_SG_05K3900_05_SG_05K3314_05); **MART_1243_99** (TT_V3928_02_TT_V2982_02_GY_V2980_02_LC_V3929_01_LC_V2979_01_AI_V2976_01_PR_V1730_03_PR_V1608_04_PR_V1044_06);

73

**PR_V1078_03** (PR_V1076_99_PR_V1623_05_PR_V1616_04_PR_V1610_04_VE_V911_01);

**SG_05K4647_05** (SG_05K4144_05_SG_05K4648_05_SG_05K3324_05_SG_05K791_05_SG_05K802_05_SG_05K2899_05_SG_05K827_05_SG_05K863_05_SG_05K805_05);

**SG_05K2418_05** (SG_05K3923_05_SG_05K871_05_SG_05K843_05_SG_05K3897_05_SG_05K3316_05_SG_05K4168_05_SG_05K4176_05_SG_05K3927_05_SG_05K3913_05); **SG_05K4454_05** (SG_05K3912_05_SG_05K3325_05_SG_05K4141_05_SG_05K852_05_SG_05K2406_05_SG_05K2400_05_SG_05K3305_05_SG_05K868_05); **SG_05K2933_05** (SG_05K2918_05_SG_05K845_05_SG_05K4182_05_SG_SS710_04); **BR_V3606_07** (BR_V3444_06_BR_V3465_06_BR_V3446_06_BR_V3423_06_BR_V3590_07_**BR_V3424_06**_BR_V3441_06_BR_V3457_06_BR_V3451_06); **BR_V3464_06** (BR_V3463_06_BR_V3431_06); **PR_V2119_02** (BR_V2380_01_BR_V2977_01_BR_V3584_06_BR_V3469_07_BR_V3430_06_BR_V3593_07_BR_V3588_07_BR_V3460_06); **NI_V3057_08** (NI_V3066_08_NI_V3068_08_NI_V2649_08_NI_V3072_08_NI_V2644_08_NI_V2934_08_NI_V3055_08_NI_V3059_08_NI_V2653_08); **VE_V2184_01** (VE_V2183_01_VE_V2180_01_VE_V2182_01_VE_V2181_01); **VE_V905_01 (**VE_V912_01_VE_V2454_01_VE_V2966_01_VE_V2191_01_VE_V2967_01_VE_V2212_03_VE_V1149_07_VE_V2484_07_VE_V1102_07_VE_V2213_03_VE_V2207_01); **VE_V2232_04** (VE_V915_01_VE_V2452_01_VE_V2199_01_VE_V2195_01_VE_V907_01_VE_V2189_01_VE_V2218_03_VE_V2214_03_VE_V2244_05_VE_V2234_04_VE_V2220_04); **VE_V1590_04**(VE_V2185_01_VE_V2231_04_VE_V2202_01_VE_V908_01_VE_V2196_01_VE_V2242_05_VE_V2222_04_VE_V2223_04_VE_V2969_03_VE_V904_01); **VE_V2201_01** (VE_V1118_01_VE_V2968_02_VE_V2455_01_VE_V2965_00_VE_V2204_01_VE_V2188_01_VE_V2203_01_VE_V2186_01_VE_V2179_00_VE_V2193_01_VE_V903_01); **PR_V1625_05** (PR_V1620_05_PR_V1622_05_PR_V1075_98_PR_V1417_07_PR_V1416_07_PR_V1415_07_PR_V1077_00_PR_V1619_05_PR_V1605_04_PR_V1604_04_PR_V1615_04);

**PR_V1478_02** (PR_V1089_03_PR_V858_03_PR_V2100_00_PR_V2110_00_PR_V2106_00_PR_V2115_01_PR_V2107_00_PR_V1453_99); **PR_V2099_98** (PR_V2104_00_PR_V2105_00_PR_V1460_00_PR_V1733_99_PR_V1454_99_PR_V1735_99_PR_V1455_99_PR_V1088_98_PR_V1450_98_PR_V1447_98); **PR_V1050_98** (PR_V1049_98_PR_V1449_98_PR_V2112_00_PR_V1080_06_PR_V859_98_PR_V2117_01_NI_V2420_94); **PR_V1621_05** (PR_V1607_04_PR_V1731_03_PR_V2123_02_PR_V1481_03_PR_V1473_02).

Identical sequences, when analyzed the NS3 protein*.* In bold are showed the strain representative.

**SG_05K4477_05** (SG_05K4440_05); **MY_22583_00** (MY_22460_00_MY_22550_00);**ET_TL018_05** (ET_TL109_05);**TH_V2313_01**(TH_V2312_01);**KH_V4306_07** (KH_V4302_07);**KH_V3809_03** (KH_V2082_03); **KH_V4298_07** (KH_V4290_07); **KH_V4308_07** (KH_V4284_07); **VN_V1877_07**(VN_V1831_07); **VN_V1330_06**(VN_V1018_07); **SG_05K3912_05** (SG_05K4141_05); **TT_V3928_02**(TT_V2982_02); **LC_V3929_01** (LC_V2979_01); **PR_V1044_06**(PR_V1078_03); **PR_V1043_06** (PR_V1618_05); **PR_V1616_04** (PR_V1610_04); **BR_V3456_06** (BR_V3417_06_BR_V3601_07); **BR_V2380_01** (BR_V2977_01);

**BR_V3606_07** (BR_V3444_06); **BR_V3465_06** (BR_V3446_06); **BR_V3457_06** (BR_V3463_06); **BR_V3584_06** (BR_V3469_07); **NI_V2934_08** (NI_V3055_08); **PR_V1091_04** (PR_V1612_04); **VE_V905_01** (VE_**V912**_01);**VE_V2480_07** (VE_V2484_07); **VE_V2213_03**(VE_V2207_01); **VE_V2200_01** (VE_V2192_01); **PR_V1733_99** (PR_V1454_99); **PR_V1415_07** (PR_V1077_00); **PR_V1605_04** (PR_V1604_04); **PR_V2108_00** (PR_V2106_00); **PR_V1736_99** (PR_V1452_99_PR_V1088_98); **LK_V2405_83** (LK_V2414_85); **VE_V2970_03** (VE_V2208_02); **TW_414_98** (TW_434_98_TW_358_98_TW_368_98_TW_388_98_TW_407_98); **KH_V3825_06** ( KH_V3822_06_KH_V3820_06_KH_V3819_06_KH_V3824_06); **VN_V1783**_**07** (VN_V1897_07_VN_V1833_07_VN_V1327_06_VN_V1015_06); **SG_05K3923_05** (SG_05K871_05_SG_05K843_05_SG_05K3316_05_SG_05K4176_05**); SG_05K3887_05** (SG_05K3329_05_SG_05K797_05); **SG_05K3897_05** (SG_05K3927_05_SG_05K3900_05_SG_05K852_05_SG_05K2918_05); **SG**_**05K4647**_**05** (SG_05K4648_05_SG_05K791_05_SG_05K802_05_SG_05K2899_05); **SG_05K827_05** (SG_05K863_05_SG_05K805_05_SG_05K2418_05_SG_SS710_04); **BR_V3442_06** (BR_V3423_06_BR_V3424_06_BR_V3441_06_BR_V3451_06**); BR_V3464_06** (BR_V3431_06); **VE_V2184_01** (VE_V2183_01_VE_V2180_01_VE_V2182_01_VE_V2181_01); **VE_V2967_01** (VE_V2452_01_VE_V2199_01_VE_V2195_01_VE_V1114_01); **VE_V2185_01** (VE_V2453_01_VE_V2178_00_VE_V2175_00_VE_V903_01); **PR_V1625_05** (PR_V1620_05_PR_V1622_05_PR_V1619_05); **PR_V1447_98** (PR_V1050_98_PR_V1049_98_PR_V1451_99_PR_V1449_98); **PR_V2112_00** (PR_V1080_06).

74

Identical sequences, when analyzed the NS4A protein*.* In bold are showed the strain representative.

**SG_05K4477_05**(SG_05K4440_05);**MY_33610_05**(MY_33927_05_MY_33506_05_MY_32645_04);IN_98901517_98(IN_98901437_98);**MY_22583_00**(MY_22460_00_MY_22550_00);**IN_PH86_04**(IN_PI64_04);**ET_TL029_05**(ET_TL018_05_ET_TL129_05);**CH_80_2**(M93130_BR_RO1_02);**TH_V2319**_**01**(TH_V2322_01);**KH_V2077_02**(KH_V2080_03);**KH_V2087_05**(KH_V2089_06_KH_V2085_05_KH_V2088_05);**KH_V4306_07**(KH_V4302_07);**H_V3804_08**(KH_V4300_07);**KH_V4280_06**(KH_V3807_05);**KH_V3809_03**(KH_V2082_03);**KH_V4298_07**(KH_V4290_07);**KH_V3828_07**(KH_V3827_07);**KH_V4294_07**(KH_V3826_07_KH_V2054_08);**VN_V1817_07**(VN_V1815_07_VN_V1326_06_VN_V1331_06);**VN_V1882_07**(VN_V1831_07_VN_V1911_08_VN_V1936_08);**SG_05K4647_05**_SG_05K4144_05_SG_05K4648_05);**TT_V3928_02**(TT_V2982_02);**LC_V3929_01**(LC_V2979_01_AI_V2976_01);PR_V1730_03(PR_V1608_04);**BR_V2983_03**(BR_V2394_05_BR_V2403_08);**BR_V3456_06**(BR_V3417_06_BR_V3601_07);**BR_V2380_01**(BR_V2977_01);**BR_V3441_06**(BR_V3431_06);BR_V3584_06(BR_V3469_07);**NI_V3072_08**(NI_V2644_08_NI_V2934_08_NI_V3055_08);PR_V1091_04(PR_V1613_04_PR_V1612_04);**CO_V3397_03**(CO_V3395_03_CO_V2978_01_CO_V3400_04);**VE_V2480**_**07**(VE_V2484_07);**VE_V2268_08**(VE_V1102_07);**VE_V1149_07**(VE_V2482_07);**VE_V1591_04**(VE_V2228_04_VE_V2218_03);**VE_V2217_03**(VE_V2215_03_VE_V2220_04);**VE_V1114_01**(VE_V2185_01_VE_V2219_03_VE_V2202_01);**VE_V2223_04**(VE_V2969_03);

**VE_V2481_07**(VE_V2266_06);**VE_V2193_01**(VE_V903_01); **PR_V1478_02**(PR_V1089_03);**PR_V1614_04**(PR_V1092_04);**PR_V1736_99**(PR_V1452_99_PR_V1737_99);**PR_V1473_02**(PR_V2114_01);**MX_V2989_07**(MX_V2987_06);**MART_1243_99**(PE_V2981_02_EC_V2975_00_NI_V2420_94);LK**_V2410_83(**LK_V2412_89);**LK_V2405_83**(LK_V2414_85);**CK_V2972_91** (PF_320219_89, PF_3056_90_PF_27643_89_PF_6056_90_PF_2956_92_PF_3050_90_WS_V2973_95);TH_V2327_01(TH_V2320_01_TH_V2318_01_TH_V2326_01_TH_V2317_01_TH_V2323_01_TH_V2313_01_TH_V2312_01);**TH_V2316_01**(TH_V2328_01_TH_V2329_01_TW_mosq_98_TW_414_98_TW_434_98_TW_390_98_TW_358_98);TW_360_98(TW_368_98_TW_503_98_TW_388_98_TW_407_98);**KH_V3822**_**06**(KH_V3820_06_KH_V4297_07_KH_V3819_06_KH_V3824_06_KH_V4308_07_KH_V4284_07_KH_V4282_07_KH_V3833_07_KH_V2083_04_KH_V2081_03);

**VN_V1783_07**(VN_V1946_08_VN_V1327_06_VN_V1017_07_VN_V1015_06_VN_V1016_06_VN_V1330_06_VN_V1018_07_VN_V1957_08_VN_V1009_06);**SG_05K3912_05**(SG_05K3325_05_SG_05K4141_05_SG_05K3928_05_SG_05K3324_05_SG_05K791_05_SG_05K802_05_SG_05K2899_05_SG_05K827_05, SG_05K863_05_SG_05K805_05_SG_05K2418_05_ SG_05K3923_05_SG_05K871_05_ SG_05K843_05_SG_05K3897_05_SG_05K3316_05_SG_05K4168_05_SG_05K4176_05_SG_05K3927_05_SG_05K4454_05_SG_05K3887_05_SG_05K3329_05_SG_05K4159_05_SG_05K4157_05, SG_05K3900_05_SG_05K3314_05_SG_05K797_05_SG_05K852_05, SG_05K2406_05_SG_05K2400_05_ SG_05K3305_05_SG_05K868_05**); SG_05K2918_05** (SG_05K845_05_ SG_SS710_04);**PR_V1044_06**(PR_V1078_03_PR_V1076_99_ PR_V1043_06_PR_V1618_05_PR_V1609_04_PR_V1616_04_PR_V1610_04);**BR_V3442_06**(BR_V3590_07_BR_V3424_06_BR_V3457_06_BR_V3451_06_BR_V3464_06_BR_V3435_06_BR_V3463_06_BR_V3430_06_BR_V3593_07_BR_V3460_06_BR_V3434_06_BR_V3605_07); **NI_V3057_08**(NI_V2654_08_NI_V3069_09_NI_V3066_08_NI_V3068_08_NI_V2649_08_NI_V3073_08_NI_V2935_08_NI_V2647_08_NI_V3059_08_NI_V2653_08);

75

**VE_V905_01**(VE_V912_01_VE_V2454_01_VE_V2247_05_VE_V2213_03_VE_V2207_01_VE_V2257_06_VE_V2232_04_VE_V2452_01_VE_V2199_01_VE_V2211_02_VE_V907_01_VE_V2205_07_VE_V2189_01_VE_V2200_01_VE_V2192_01_VE_V1115_01_VE_V2196_01_VE_V2224_04_VE_V916_01_VE_V904_01_VE_V2201_01_VE_V2197_01_VE_V1118_01_VE_V2210_02);**VE_V2198_01(**VE_V1116_01_VE_V913_01_VE_V2965_00_VE_V2178_00_VE_V2174_00_VE_V2175_00_VE_V2186_01_VE_V2184_01_VE_V2183_01_VE_V2180_01_VE_V2182_01_VE_V2181_01_VE_V2179_00);

**PR_V1625_05**(PR_V1620_05_PR_V1622_05_PR_V1075_98_PR_V1415_07_PR_V1077_00_PR_V1619_05_PR_V1605_04_PR_V1604_04_PR_V2098_99_PR_V2099_98_PR_V2120_02_PR_V1732_02_PR_V2104_00_PR_V2105_00_PR_V1733_99_PR_V1454_99_PR_V1735_99_PR_V1731_03_PR_V2123_02_PR_V1491_03_PR_V1481_03_PR_V1626_05_PR_V1606_04_PR_V1455_99_PR_V1453_99_PR_V1088_98_PR_V1450_98_PR_V1447_98_PR_V1050_98_PR_V1049_98_PR_V1451_99_PR_V1449_98_PR_V2103_00_PR_V1466_99_PR_V2111_00_PR_V1080_06_PR_V859_98_PR_V2117_01);

Identical sequences, when analyzed the NS4B protein*.* In bold are showed the strain representative.

**SG_05K4477_05** (SG_05K4440_05);**MY_34640_05** (MY_33610_05_MY_33927_05_MY_32645_04_ MY_28526_00); **IN_TB55i_04** (IN_KJ30i_04);**IN_98901517_98** (IN_98901437_98);**MY_22583_00** (MY_22460_00_MY_22550_00);**ET_TL029_05** (ET_TL129_05);**PF_2986_92** (PF_320219_89_PF_27643_89_PF_6056_90);**TH_V2327_01** (TH_V2326_01);**TH_V2313_01** (TH_V2312_01);**KH_V2077_02** (KH_V2080_03);KH_V2079_02 (KH_V2078_02);**KH_V4286_07** (KH_V2087_05_KH_V2089_06_KH_V2085_05_KH_V2088_05);**KH_V3825_06** (KH_V3822_06_KH_V3820_06_KH_V3819_06_KH_V3824_06);**KH_V4280_06** (KH_V3807_05);**KH_V2054_08** (KH_V2053_08);**VN_V1817_07** (VN_V1790_07);**VN_V1329_06** (VN_V1008_06);**VN_V1831_07** (VN_V1936_08);**VN_V1783_07** (VN_V1897_07);**BD_7_02** (BD_3_02);**BD_4_02** (BD_1_02);TT_V3928_02 (TT_V2982_02);PR_V1730_03 (PR_V1608_04);**BR_V3456_06** (BR_V3417_06);**BR_V2380_01** (BR_V2977_01);BR_V3444_06 (BR_V3465_06_BR_V3446_06);**BR_V3584_06** (BR_V3469_07_BR_V3593_07);BR_V3588_07 (BR_V3460_06_BR_V3434_06); **NI_V2654_08** (NI_V3068_08_NI_V2649_08_NI_V3073_08); **NI_V72_08** (NI_V2644_08_NI_V2934_08_NI_V3055_08); **NI_V2935_08** (NI_V2647_08);PR_V1091_04 (PR_V1613_04); **VE_V905_01** (VE_V912_01);VE_V1149_07 (VE_V2480_07_VE_V2484_07_PR_V2126_06);VE_V2213_03 (VE_V2207_01);VE_V2452_01 (VE_V2199_01);**VE_V2205_07** (VE_V2189_01);VE_V2228_04 (VE_V2218_03);VE_V2242_05 (VE_V2222_04_VE_V2969_03);**VE_V1116_01** (VE_V913_01);VE_V2970_03 (VE_V2208_02);**VE_V2184_01** (VE_V2183_01);PR_V2120_02 (PR_V1732_02);PR_V1625_05 (PR_V1620_05_PR_V1622_05_PR_V1416_07);R_V1731_03 (PR_V2123_02);PR_V1605_04 (PR_V1604_04_PR_V1729_03_PR_V1615_04_PR_V2118_01);PR_V1614_04 (PR_V1092_04_PR_V1475_02);**VE_V2192_01** (PR_V2110_00);PR_V1050_98 (PR_V1049_98);**PR_V1449_98** (PR_V2112_00);PR_V1466_99 (PR_V2111_00);PR_V1080_06 (PR_V859_98);**MX_V2989_07** (MX_V2987_06);LK_V2410_83 (LK_V2412_89);LK_V2405_83 (LK_V2414_85_LK_V2407_83TH_V2320_01_TH_V2318_01_TH_V2319_01_TH_V2317_01); **TH_V2321**_**01 (**TH_V2322_01_TH_V2323_01_TH_V2316_01_TH_V2328_01); **TW_mosq_98** (TW_414_98_ TW_434_98_TW_390_98_TW_358_98TW_360_98_TW_368_98_TW_503_98_ TW_388_98_TW_407_98);**KH_V4308_07** (KH_V4298_07_KH_V4290_07_KH_V4284_07_ KH_V3815_06); **CH_CHLS001_07** KH_V2051_07); **VN_V1882_07** (VN_V1810_07_VN_V1891_07_VN_V1946_08); **VN_V1824_07**_VN_V1833_07_VN_V1327_06_ VN_V1017_07_VN_V1015_06);**SG_05K4647_05** (SG_05K4144_05_SG_05K4648_05_SG_05K802_05_SG_05K827_05_SG_05K863_05); **SG_05K3887_05** (SG_05K3329_05_SG_05K4159_05_SG_05K4157_05_SG_05K3900_05 _SG_05K797_05_SG_05K852_05_SG_05K2406_05_SG_05K2400_05); **SG_05K843_05** (SG_05K3305_05_SG_05K868_05_SG_05K2933_05_SG_05K2918_05_SG_05K845_05_SG_05K4182_05);SG_05K3324_05 (SG_05K791_05_SG_05K2899_05_SG_05K805_05_ SG_05K2418_05_ SG_05K3923_05_ SG_05K871_05_SG_05K3897_05_SG_05K3316_05_SG_05K4168_05_ SG_05K3927_05_SG_05K3913_05_SG_05K4454_05_SG_05K3912_05_SG_05K3325_05_SG_05K4141_05_SG_SS710_04);**PR_V1044_06** (PR_V1078_03_PR_V1076_99_PR_V1043_06_PR_V1618_05_ PR_V1623_05_PR_V1609_04_PR_V1616_04_PR_V1610_04);**BR_V3442_06** (BR_V3423_06_BR_V3590_07_BR_V3424_06_BR_V3441_06_BR_V3457_06_BR_V3451_06_BR_V3464_06_BR_V3435_06_BR_V3463_06_BR_V3431_06);**VE_V2455_01** (VE_V2453_01_VE_V2965_00_VE_V2204_01_VE_V2188_01_VE_V2203_01_VE_V2178_00_VE_V2174_00_VE_V2175_00);**VE_V2454_01** (CO_V3397_03_CO_V3401_04_CO_V3395_03_ VE_V2966_01_ VE_V2967_01_VE_V1117_01_VE_V907_01_VE_V2185_01_VE_V2202_01_ VE_V908_01_PR_V2100_00_PR_V1465_00_PR_V2106_00_PR_V2107_00); **VE_V2190_01** (VE_V2186_01_ VE_V2180_01_VE_V2182_01_VE_V2181_01_VE_V2179_00_PR_V2098_99_ PR_V2099_98_PR_V1460_00_PR_V1733_99_PR_V1735_99_PR_V1455_99_PR_V1736_99_PR_V1452_99_PR_V1737_99_PR_V1088_98_PR_V1450_98_PR_V1447_98_PR_V1451_99);

76

Identical sequences, when analyzed the NS5 protein*.* In bold are showed the strain representative.

**SG_05K4477_05** (SG_05K4440_05); **MY_33610_05** (MY_33927_05);**IN_98901517_98** (IN_98901437_98); **MY_22583_00** (MY_22460_00);**TH_V2313_01** (TH_V2312_01);**KH_V4306_07** (KH_V4302_07);**KH_V3822_06** (KH_V3819_06); **KH_V3820_06** (KH_V3824_06);**KH_V4298_07** (KH_V4284_07); **KH_V3828_07** (KH_V3827_07);**VN_V1824_07** (VN_V1833_07);**SG_05K3897_05** (SG_05K3316_05); **SG_05K3923_05** (SG_05K4176_05_SG_05K3927_05);**SG_05K3912_05** (SG_05K3325_05_SG_05K4141_05);**SG_05K3887_05** (SG_05K3329_05);**TT_V3928_02** (TT_V2982_02);**PR_V1623_05** (PR_V1609_04);**BR_V3456_06** (BR_V3417_06);**BR_V2380_01** (BR_V2977_01);BR_V3465_06 (BR_V3446_06);**BR_V3424_06** (BR_V3457_06_BR_V3463_06_BR_V3431_06);**NI_V3057_08** (NI_V2654_08);**NI_V2644_08** (NI_V3055_08);**VE_V2480_07** (VE_V2484_07);VE_V2452_01 (VE_V2199_01);**VE_V2217_03** (VE_V2215_03);**VE_V2178_00** (VE_V2174_00_VE_V2175_00);**PR_V2104_00** (PR_V1460_00);**PR_V1605_04** (PR_V1604_04);**PR_V2100_00** (PR_V2115_01);**TH_V2327_01** (TH_V2320_01_TH_V2318_01_TH_V2319_01_TH_V2326_01_TH_V2317_01_TH_V2321_01);**TW_mosq_98** (9TW_414_98_TW_434_98_TW_390_98_TW_358_98_TW_368_98_TW_503_980); **SG_05K791_05** (SG_05K802_05_SG_05K2899_05_SG_05K827_05_SG_05K863_05 _SG_05K805_05); **SG_05K843_05** (SG_05K4454_05_SG_05K3314_05_SG_05K3305_05_ SG_05K868_05_SG_05K845_05); **BR_V3442_06** (BR_V3423_06_BR_V3451_06_ BR_V3464_06_BR_V3435_06); **VE_V2184_01** (VE_V2183_01_VE_V2180_01_ VE_V2182_01_VE_V2181_01); **VE_V915_01** (VE_V2185_01_VE_V903_01_PR_V2099_98_ PR_V1088_98_ PR_V1447_98_PR_V1050_98_PR_V1049_98_PR_V859_98);
